# Nuclear Magnetic Resonance Fingerprinting and Principal Component Analysis Strategies Lead to Anti-Tuberculosis Natural Product Discovery from Actinomycetes

**DOI:** 10.3390/antibiotics14010108

**Published:** 2025-01-20

**Authors:** Jianying Han, Xueting Liu, Lixin Zhang, Ronald J. Quinn, Miaomiao Liu

**Affiliations:** 1 Institute for Biomedicine and Glycomics, Griffith University, Brisbane, QLD 4111, Australia; r.quinn@griffith.edu.au; 2 Institute for Molecular Bioscience, The University of Queensland, St Lucia, QLD 4072, Australia; 3 State Key Laboratory of Bioreactor Engineering, East China University of Science and Technology, Shanghai 200237, China; liuxueting@ecust.edu.cn (X.L.); lxzhang@ecust.edu.cn (L.Z.)

**Keywords:** tuberculosis, actinomycetes, borrelidin, elaiophylin, polycyclic tetramate macrolactams, bafilomycin

## Abstract

Background: The increasing prevalence of drug-resistant tuberculosis (TB) underscores the urgent need for novel antimicrobial agents. Methods: This study integrates cultivation optimization, nuclear magnetic resonance (NMR) fingerprinting, and principal component analysis (PCA) to explore microbial secondary metabolites as potential anti-TB agents. Results: Using the combined approach, 11 bioactive compounds were isolated and identified, all exhibiting anti-*Mycobacterium bovis* BCG activity. Notable findings include borrelidin, a potent threonyl-tRNA synthetase inhibitor with broad biological activities, and L-O-Lac-L-Val-D-O-Hiv-D-Val, a peptide isolated for the first time from a plant endophyte, demonstrating broad-spectrum antimicrobial activity. Additionally, elaiophylin and polycyclic tetramate macrolactams (PTMs) displayed significant bactericidal effects, with elaiophylin achieving complete BCG inhibition at 72 h and PTMs marking their first reported anti-TB activity. The study also identified bafilomycins as potent scaffolds for anti-TB drug development, showcasing rapid bactericidal activity at low MIC values. Conclusions: These findings emphasize the value of microbial metabolites as a reservoir of bioactive compounds and provide new avenues for developing next-generation anti-TB therapies.

## 1. Introduction

Tuberculosis (TB) has reclaimed its position as the world’s leading cause of death from a single infectious agent after being surpassed by coronavirus disease (COVID-19) for three years [1]. While only a small percentage (5–15%) of infected individuals develop clinical TB, there were approximately 10.8 million new cases and 1.25 million deaths in 2023 [1]. *Mycobacterium tuberculosis* (*Mtb*) is therefore responsible for more human mortality than any other single microbial species. A number of efficacious anti-tubercular agents were discovered in the late 1940s and 1950s, with rifampicin introduced in the 1960s [2]. Since that time, two compounds, bedaquiline and delamanid, have been approved by the FDA (Food and Drug Administration) for use against MDR-TB [3]. However, the emergence of multi-drug-resistant strains of *Mtb* makes it necessary for the discovery of new drugs.

Natural products have been an effective starting points for anti-TB drug discovery [4]. Seven out of the current eleven nature-derived TB drugs were either isolated from microorganisms or chemically modified from active microbial natural products. A review summarizing anti-TB natural products with defined target proteins identified that more than half (57%) of the compounds were isolated from bacteria, particularly *Streptomyces* [5]. Furthermore, bacteria-derived compounds exhibited stronger anti-TB activity (<0.1 μM, 23.3%; 0.1–1 μM, 36.7%) compared to plant- and sponge-derived compounds (<0.1 μM, 0%; 0.1–1 μM, 0%), highlighting the significance of actinomycetes in producing potent anti-TB compounds [5].

Microorganisms collected from unique environments, such as from the sea, plants, and deserts, appear to have adapted to their extreme environments, thus gaining their diverse metabolic and genetic capabilities [6]. The harsh chemical and physical conditions of the sea have favored the production of a great variety of novel molecules in marine organisms that are unique in terms of diversity, both structural and functional [7,8]. A literature search targeting natural products isolated from marine microbes with reported anti-TB activity resulted in 60 compounds, 16 of which were found to have anti-TB activity, with MICs less than 10 μM, suggesting that marine microbes are a promising source for anti-TB drug exploration [9]. Endophytes, which form symbiotic relationships with plants, were found to play a crucial role in producing bioactive compounds by directly releasing metabolites to combat phytopathogens and indirectly enhancing host defense or growth [10]. Desert-derived actinomycetes, with their specialized metabolisms and adaptability to extreme conditions, are also proven as promising sources of bioactive compounds for drug discovery [11].

One challenge in microbial natural product discovery is that many secondary metabolites are not produced under standard laboratory culture conditions. For example, research into marine microbes in particular is hampered by the level of knowledge concerning the basic biology and culture techniques for marine-derived organisms, and many more applications of methods designed for the isolation, culture, and chemical investigation of these organisms are needed [12]. OSMAC (one strain-many compounds) is a strategy to maximize the productivity of a single microbe through turning on silent or cryptic biosynthetic genes by varying accessible cultivation parameters, such as media composition, pH, temperature, oxygen supply, quality and quantity of light, and bioreactor platform, or the addition of precursors or enzyme inhibitors [13]. Some examples of active microbial natural products with the use of the OSMAC strategy are documented in Table 1.

Another challenge in bioassay-guided isolation is that it is generally long and laborious, with activity often lost or significantly reduced during compound isolation. We have previously introduced strategies to overcome these limitations and to effectively isolate active components from crude extracts. For example, by fractionating crude extracts using HPLC, compounds with favorable drug-like properties can be concentrated, while reducing complexity in the fractions compared to the original crude extracts, making the bioactivity results of the fractions more reliable for further purification [21]. We have also incorporated NMR analysis alongside bioactivity assays of the generated fractions, where the ^1^H NMR spectra of each fraction provide a distinct fingerprint of its components [22]. By isolating compounds associated with all detectable NMR signals within the fraction’s fingerprint, it is highly probable that the active component is isolated.

To streamline data analysis, we also developed an approach for analyzing large NMR fingerprint datasets (>4000 ^1^H NMR spectra) to highlight unique secondary metabolites within the entire dataset [23]. This approach facilitates the identification of distinct secondary metabolites and, when combined with the OSMAC strategy, helps optimize culture conditions for specific strains. Moreover, integrating bioactivity data with NMR fingerprints allows for linking bioactivity to specific NMR signals, providing insights into the components potentially responsible for bioactivity.

In this study, we applied HPLC fractionation, ^1^H NMR fingerprinting, PCA data analysis, and bioactivity screening to five strains to discover bioactive compounds. This approach resulted in the isolation of 11 secondary metabolites, all of which exhibited strong anti-mycobacterial activity, making them promising candidates for future drug discovery.

## 2. Results

### 2.1. Evaluation of Anti-Mycobacterial Activity of 654 Actinomycete Cultures in Six Media

This study involved the cultivation of 654 actinomycete strains, comprising 329 land strains collected from the Taklimakan Desert, 125 marine strains from the South China Sea, and 200 endophytic strains isolated from various traditional Chinese medicines (TCMs) [24]. Each land and marine strain was cultured in three distinct media: a nutrient-deficient medium (NM2), a standard laboratory medium (AM2), and a nutrient-rich medium (MPG). The endophyte strains were additionally cultured in three more modified media (M001, M12, and M21), totaling six media types. Methanol and ethyl acetate extracts were combined from each culture, yielding 2562 crude extracts.

**Figure 1:**
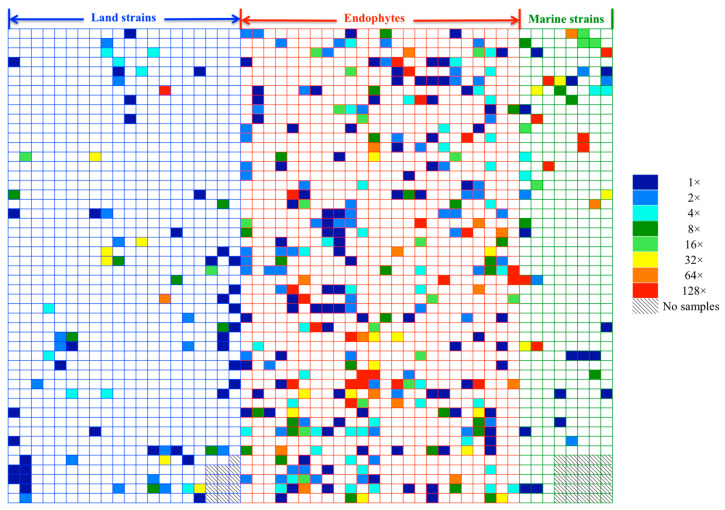
Heatmap of the anti-BCG screening on crude extracts: 2562 samples were prepared from 654 strains isolated from land strains (blue blanks), endophytes (red blanks), and marine microbes (green blanks), color coded according to the legend (blue lowest and red highest activity).

**Figure 2:**
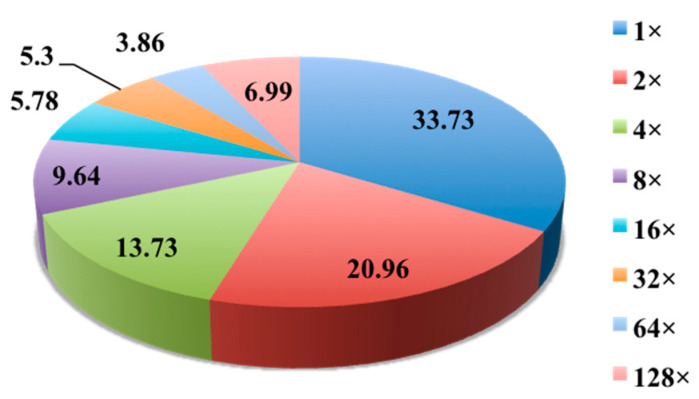
Pie chart of anti-BCG activities of 415 active crude extracts.

To identify anti-mycobacterial natural products, all crude extracts were screened against *Mycobacterium bovis* BCG using a previously described cell-based assay, with activity data presented in a heatmap (Figure 1). In total, 415 of the 2562 extracts (16.2%) exhibited positive results in the single-dose anti-BCG screening assay (Figure 2). Active extracts were further examined in a serial dilution assay. Approximately one-third (33.73%) of these extracts showed efficacy against over 90% of BCG bacilli at the highest concentration (1×). However, the efficacy decreased with higher dilutions; only 5.3% and 3.86% of active extracts retained activity at dilution factors of 32 and 64, respectively. Remarkably, nearly 7% of the active extracts were effective at a 128-fold dilution, indicating strong potential for further chemical investigation. The high activity observed at this dilution suggests that certain strains exhibit significant anti-BCG activity when cultured under various media conditions.

**Figure 3:**
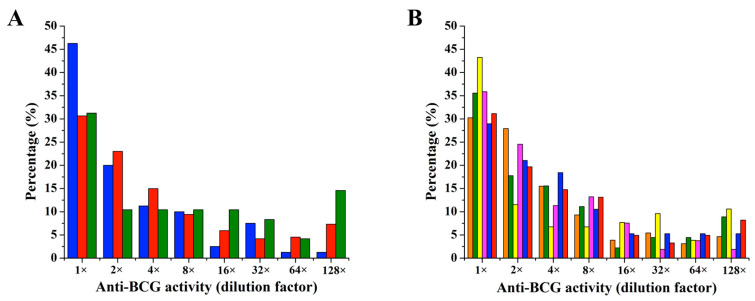
Histograms of the percentage of anti-BCG activities of 2562 microbial crude extracts. (**A**) Biological sources: land strains (blue), endophytes (red), and marine strains (olive). (**B**) Different media: AM2 (orange), NM2 (olive), MPG (yellow), M001 (magenta), M12 (blue), and M21 (red).

The proportion of active extracts among land, endophytic, and marine strains was 8.1%, 23.9%, and 12.8%, respectively. Endophytic and marine actinomycetes were generally more likely than land strains to produce active metabolites. About half of the active land extracts were only effective at the highest concentration tested, with only ~2.5% showing activity at dilution factors of 64 (1.25%) or 128 (1.25%) (Figure 3A). Despite the substantial number of endophytic extracts displaying anti-BCG activity, only about 20% retained activity at dilutions greater than 8. It has been suggested that endophytes may have adapted to their unique environments over evolutionary timescales, potentially incorporating plant DNA into their genomes [25]. The endophytes examined in this study were derived from various TCM plants, possibly explaining their biosynthesis of phytochemicals characteristic of their host plants. This may also account for the high activity rates of endophyte extracts in the anti-BCG assay, though only a modest number of extracts displayed strong activity, aligning with the generally moderate therapeutic properties of TCMs. In contrast, approximately 15% of active marine extracts exhibited anti-BCG activity at a 128-fold dilution, the second-highest proportion observed, underscoring the potential of marine actinomycetes for TB drug discovery.

The impact of the six culture media on the production of anti-BCG metabolites by the 654 actinomycetes is summarized in Figure 3B. Among active samples, media M9, M001, and AM2 demonstrated the greatest potential for inducing active metabolite synthesis, with positive rates of 30.5% (in 200 endophyte samples), 26.5% (in 200 endophyte samples), and 19.7% (in 654 samples), respectively. The media MPG, M21, and M12 were most effective at retaining activity at dilution factors greater than 8, with proportions of 31.7%, 21.3%, and 21.1%, respectively. Consistent with previous findings, MPG and M21 were also identified as the most productive media among the ten tested fermentation media [26].

A total of five strains, including three marine strains, MS110104, MS110167, and MS110105; one endophyte strain, ES120127; and one land strain, LS120167, with retained activity at 128 dilution factors, were selected for larger scale investigations and are discussed below.

### 2.2. Chemical and Biological Investigation of Five Selected Strains Cultured in 32 Conditions

#### 2.2.1. Platform for Discovery of Unique Bioactive Secondary Metabolites

**Figure 4:**
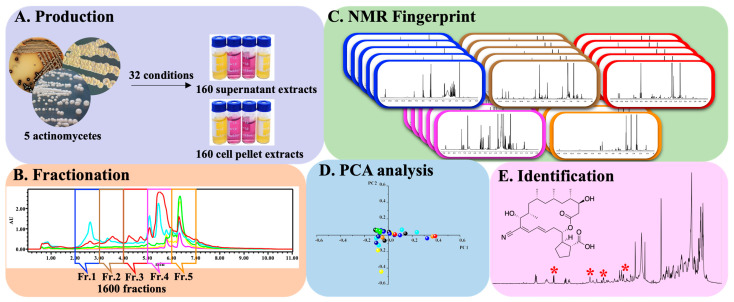
Overview of the strategy. (**A**) Production: each strain was cultivated under 32 conditions, resulting in 320 crude extracts (160 supernatant extracts and 160 cell pellet extracts) per strain. (**B**) Fractionation: each extract was fractionated by HPLC to generate 5 fractions, resulting in 1600 fractions per strain. (**C**) NMR fingerprint: each fraction was analyzed by ^1^H NMR to generate a fingerprint. (**D**) PCA analysis: NMR fingerprints for each strain were analyzed by PCA to identify outliers. (**E**) Identification: unique NMR signals (red asterisk) from the identified outlier fractions led to the identification of the responsible secondary metabolites.

To maximize the active compound production capability of the five selected strains, they were cultured under a total of 32 distinct conditions following the published strategy [23]. These 32 conditions included 10 culture media with two culture periods (7 days and 14 days, totaling 20 conditions), 10 stress conditions (3 heat shocks, 3 ethanol shocks, 3 pH shocks, and one standard control), and the addition of inducer molecules (two inducers: L-homoserine lactone and N-carbobenzoxy-L-homoserine lactone). The extraction process, following published procedures to maximize the yield of secondary metabolites, involved centrifugation followed by extracting supernatants with *n*-butanol and cell pellets with acetone [27]. This yielded 320 crude extracts, consisting of 160 supernatant extracts and 160 cell pellet extracts from the five microbes for further examination (Figure 4A).

While crude extracts are cost-effective and require minimal preparation time, they often face limitations, such as the potential for minor metabolites to remain undetected due to interference from complex mixture components. Recognizing the need to isolate the most bioactive compounds, we determined that fractionation of natural product crude extracts could provide an optimal solution. Fractionation reduces compound complexity, allowing further analyses, such as NMR fingerprinting, to capture the true bioactive compounds within the crude extracts/fractions. It is now over 10 years since we published the paper on the development of natural product fraction libraries [21]. We introduced a fractionation procedure for each crude extract by using reversed-phase solvent conditions (MeOH/H_2_O/0.1% TFA) and a Phenomenex C_18_ Monolithic HPLC column (4.6 mm × 100 mm). In the published method, 11 fractions were collected per extract within the region of the chromatogram corresponding to log P < 5 [21]. This fractionation provides a second log P filter, allowing any remaining high log P components to be excluded, delivering lead- and drug-like molecules separated into mixtures of a small number of compounds to facilitate the rapid identification of active molecules. To simplify the protocol, we then decided to collect five fractions per extract within the same chromatogram region (Figure 4B).

Based on a simple visual examination of chromatograms, the pre-fractionation method shows several qualitative benefits. Early eluting polar components are removed from the samples. The overall complexity of the samples to be tested in HTS is reduced with fewer compounds per well. Suitable resolution was achieved, and the major components that may interfere with the activity of minor components are separated into individual fractions. We used this modified chromatography method for the obtained 320 crude extracts, which resulted in a fraction library of 1600 fractions, with 320 fractions for each strain. We then obtained NMR fingerprints (^1^H NMR) on each fraction to resolve the constituents (Figure 4C).

Analysis of metabolites in each fraction is key to targeting the unique compounds only produced by the microbes under specific culture conditions. Principal component analysis (PCA) was conducted on the 320 NMR fingerprints from each strain to pinpoint the most unique fractions (Figure 4D). It revealed clusters of fractions with similar metabolic profiles, as well as distinct outliers that suggested unique chemical compositions. By focusing on these outliers in the score plot and their corresponding signals in the loading plot, we identified key NMR signals that differentiated these fractions from the rest. This correlation allowed us to pinpoint specific metabolites with high potential for bioactivity, thereby narrowing down the pool of compounds for detailed isolation and structural elucidation (Figure 4E).

#### 2.2.2. PCA Analysis to Highlight the Metabolites Corresponding to the Bioactivity

**Figure 5:**
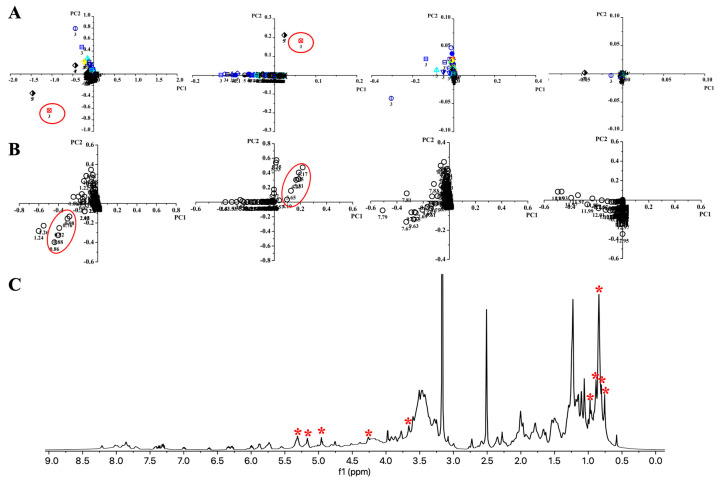
PCA results of 320 fractions from MS110167. Anti-BCG activity is indicated by color coding according to the legend in Figure 1 (blue for lowest activity, red for highest activity). (**A**) From left to right, score plots of NMR chemical shifts in the regions 0–2.4 ppm, 3.5–6 ppm, 6–10 ppm, and 10–15 ppm. Each type of symbol represents fractions derived from an OSMAC condition (see symbol details in the Supplementary Figure S1). Fraction numbers are indicated below each symbol, with the supernatant fractions labeled as 1 to 5 and the cell pellet fractions labeled as 1′ to 5′. The fraction highlighted with a red circle is the selected fraction: fraction 3, derived from the supernatant extract cultured at pH 5.5. (**B**) Loading plots of NMR chemical shifts for the regions 0–2.4 ppm, 3.5–6 ppm, 6–10 ppm, and 10–15 ppm. Bucket values in the loading plots are shown as numbers below each circle (in ppm). Bucket values responsible for the outlier in (**A**) are highlighted in red cycle. (**C**) ^1^H NMR spectrum (acquired in DMSO-*d*_6_) of the selected outlier (fraction 3 from the supernatant extract cultured at pH 5.5). Unique NMR signals identified by PCA loading plots are marked with red asterisks.

Multivariate statistical analysis was performed on a dataset consisting of NMR fingerprints from 320 fractions for each strain. Each fraction’s NMR fingerprint (0–15 ppm) was processed in AMIX to generate 696 buckets (bucket width: 0.02 ppm), representing 696 variables. By analyzing the resulting 320 × 696 matrix for each strain, PCA score plots were used to distinguish between fractions from different culture conditions (Figure 5A). The metabolites responsible for these distinctions are indicated in the loading plots (Figure 5B). When analyzing the entire NMR fingerprint region (0–15 ppm) together, only a few outliers were identified. Complete analysis of all buckets was limited because the principal component values were predominantly influenced by strong signals in the high-field region, making it challenging to differentiate between low-intensity buckets in the downfield region. To improve differentiation, the ^1^H NMR spectra of the fractions were assessed in separate chemical shift regions (0–2.4 ppm, 3.5–6 ppm, 6–10 ppm, and 10–15 ppm) for PCA analysis.

Figure 5 presents the PCA results for marine strain MS110167. Four score plots highlight outliers among the 320 fractions, each with unique NMR signals (Figure 5A), while corresponding loading plots display the NMR signals responsible for these outliers (Figure 5B). In total, 14 outliers were identified across the four score plots for MS110167, with 4 of these appearing in multiple NMR regions. Fraction 3, derived from the supernatant extract cultured at pH 5.5 (pH shock), was identified as a significant outlier in the 0–2.4 ppm and 3.5–6 ppm regions and also demonstrated strong bioactivity against BCG (dilution factor 128×). The associated loading plots showed the NMR signals responsible for this outlier, with chemical shifts at 0.76–0.98 ppm (region 1) and at 3.65, 4.25, and 4.95–5.31 ppm (region 2), which were clearly observed in the corresponding NMR fingerprint for this fraction (Figure 5C). A few other outliers with unique NMR signals, such as fraction 5 from the cell pellet extract cultured in medium 9 and fraction 3 from the supernatant extract cultured in medium 4, were also noted. However, these fractions showed no or low activity against BCG (dilution factor 1×) and were not selected for further investigation.

**Figure 6:**
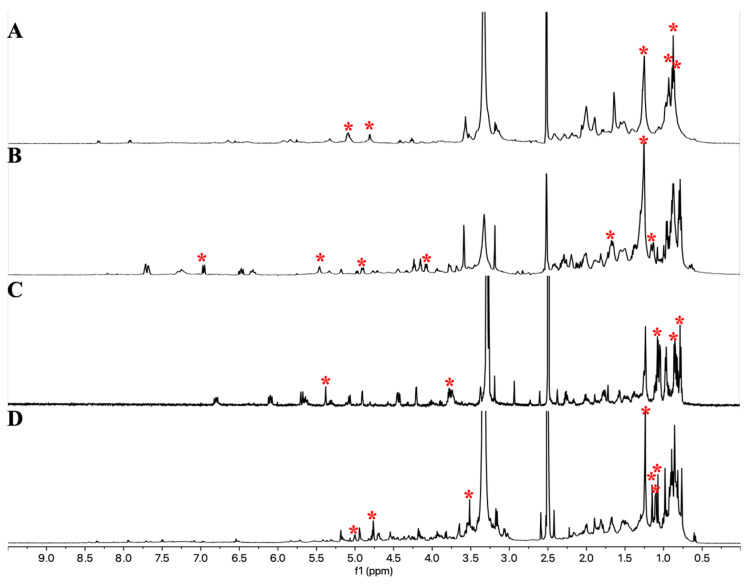
^1^H NMR spectra (acquired in DMSO-*d*_6_) of the selected outliers from ES120127 (**A**), LS120167 (**B**), MS110105 (**C**), and MS110104 (**D**). Unique NMR signals identified by PCA loading plots are marked with red asterisks.

Similarly, PCA analysis was conducted on the NMR fingerprints of the other four selected strains, and one fraction from each was chosen for metabolite isolation (Supplementary Figures S2–S5). The selection was based on PCA outlier analysis combined with the bioactivity of the fractions. As a result, the following fractions were selected: fraction 3 from the supernatant extract cultured in medium 4 for endophyte strain ES120127, fraction 4 from the supernatant extract cultured in medium 6 for land strain LS120167, fraction 5 from the supernatant extract cultured at pH 5.5 for marine strain MS110105, and fraction 3 from the supernatant extract cultured with the inducer molecule N-carbobenzoxy-L-homoserine lactone for marine strain MS110104. The unique NMR signals for these fractions, as identified through PCA analysis (Supplementary Figures S2–S5), are displayed in Figure 6.

#### 2.2.3. Purification and Identification of Newly-Generated Secondary Metabolites from the Selected Four Microbes

**Figure 7:**
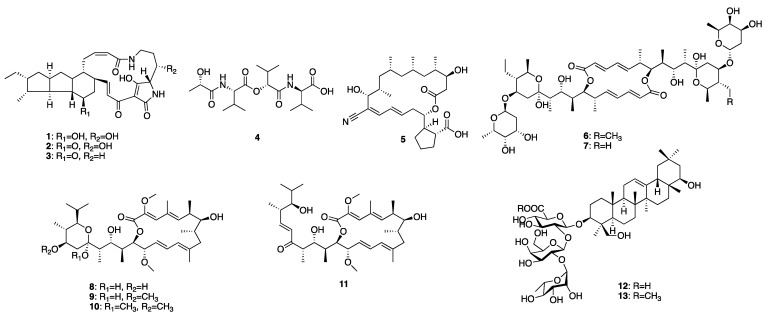
Chemical structures of isolated compounds **1**–**13**.

A 4 L fermentation of each strain was conducted under the corresponding conditions. The same extraction and fractionation procedures were followed to generate the target fractions: fraction 3 for MS110167, ES120127, and MS110105; fraction 4 for LS120167; and fraction 5 for MS110105. Guided by the NMR chemical shifts identified previously, a total of 13 secondary metabolites were isolated from these fractions using a series of chromatographic techniques and identified through combined NMR spectroscopy and mass spectrometry analysis (Figure 7).

Isolation from MS110167 yielded three polycyclic tetramate macrolactams (PTMs): dihydromaltophilin (**1**) [28,29,30,31], xanthobaccin A (**2**) [32,33,34], and xanthobaccin C (**3**) [35]. From ES120127, a peptide, L-O-Lac-L-Val-D-O-Hiv-D-Val (**4**) [36], was identified. Isolation from LS120167 resulted in the identification of a polyketide macrolide, borrelidin (**5**) [37,38,39]. From MS110105, two macrodiolides, elaiophylin (**6**) [40,41] and efomycin G (**7**) [42], were isolated. Finally, isolation from MS110104 yielded four macrolides: bafilomycin A1 (**8**) [43,44], bafilomycin G (**9**) [45], bafilomycin H (**10**) [45], and bafilomycin D (**11**) [46,47]. The NMR chemical shifts identified in each fraction through PCA analysis matched the corresponding purified compounds, except for the up-field signals at 0.76–0.98 ppm in the MS110167 fraction. These signals were attributed to two saponins, soyasaponin I (**12**) and soyasaponin I methyl ester (**13**), also purified from this fraction. As these saponins originated from the soy-based culture medium, where they are major constituents, they were excluded from further investigation.

#### 2.2.4. Antimicrobial Activity Evaluation

Compounds **1**–**11** were tested in in vitro screening against *Mycobacterium*, including *M. bovis* BCG, *Mtb* mc^2^ 6220 biosafety level 2 (BSL2) strain, as well as the wild type strain *Mtb* H37Rv (Table 2). All tested compounds exhibited inhibitory activity against BCG, which served as the model of activity for the crude extracts and fractions. This demonstrates the effectiveness of the HPLC-NMR-PCA platform in identifying highly active components from a large dataset.

Eight compounds, dihydromaltophilin (**1**), L-O-Lac-L-Val-D-O-Hiv-D-Val (**4**), borrelidin (**5**), elaiophylin (**6**), efomycin G (**7**), bafilomycin A1 (**8**), bafilomycin G (**9**), and bafilomycin H (**10**), exhibited MICs against BCG growth below 10 μg/mL, with the most potent compound being borrelidin (**5**) and elaiophylin (**6**), with MIC at 0.78 and 1.56 μg/mL, respectively. All eleven compounds showing BCG activity were further tested for the inhibition of the *Mtb* biosafety level 2 (BSL2) strain mc^2^ 6220. Unfortunately, all PTMs **1**–**3** and two bafilomycins, **10** and **11**, lost their ability to inhibit growth, while the other compounds retained various levels of activity. Notably, elaiophylin (**6**), one of the most potent BCG inhibitors, retained activity against mc^2^ 6220, with the MIC doubled at 3.125 μg/mL. Surprisingly, bafilomycin A1 (**8**) demonstrated higher potency against mc^2^ 6220, with an MIC of 3.89 μg/mL compared to its BCG MIC of 6.25 μg/mL, suggesting a unique mechanism of action. However, neither compound showed significant inhibition against the wild-type strain *Mtb* H37Rv.

Among the PTMs, dihydromaltophilin (**1**) exhibited the strongest anti-BCG activity, with an MIC value of 1.56 μg/mL. The replacement of the 14-OH group with a carbonyl group in xanthobaccin A (**2**) reduced activity, indicating the critical role of the 14-OH group for anti-BCG efficacy. The lack of activity in compound **3** at concentrations up to 100 μg/mL further confirmed the essential role of both hydroxyl groups in inhibiting BCG growth.

Compared to compounds **8** to **10**, compound **11** showed weaker anti-BCG activity and an MIC value twice that of the others (12.5 μg/mL), suggesting that the presence of the pyranose moiety may be important for the inhibitory process.

Activity evaluation of compounds **1**–**11** against three Gram-positive bacteria (*Staphylococcus aureus* (SA), methicillin-resistant *S. aureus* (MRSA), *Bacillus subtilis* (BS)) and one Gram-negative bacterium (*Pseudomonas aeruginosa* (PA)) was also carried out (Table 2). All PTMs **1**–**3** showed no significant inhibition against any of the tested strains, suggesting the high specificity of them for BCG. Four bafilomycins, **8**–**11**, also demonstrated limited activity in the tested models, with only weak inhibition against SA with MIC values of 50 μg/mL. The peptide L-O-Lac-L-Val-D-O-Hiv-D-Val (**4**) showed comparable activity against SA and BS, as it did against BCG, with MICs of 3.125 μg/mL for each. Its activity was four times weaker against the drug-resistant MRSA strain, with an MIC of 12.5 μg/mL. Elaiophylin (**6**) emerged as the most potent compound across the tested microbial pathogens, with MICs of 0.78 μM and 1.56 μg/mL against SA and BS, respectively. Although its activity was slightly reduced against the drug-resistant MRSA strain, it retained an MIC of 1.56 μg/mL, highlighting its potential as a robust antimicrobial agent.

**Figure 8:**
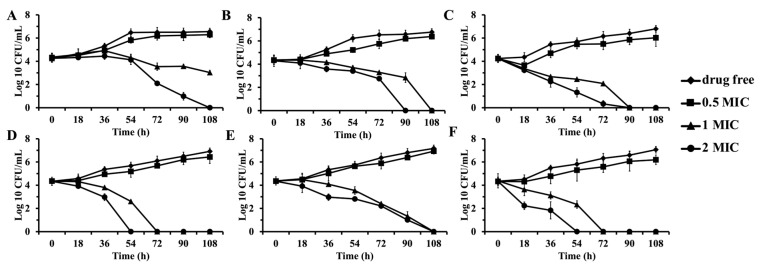
Time-kill curves of five selected compounds and positive control against BCG. (**A**) dihydromaltophilin (**1**), (**B**) L-O-Lac-L-Val-D-O-Hiv-D-Val (**4**), (**C**) borrelidin (**5**), (**D**) elaiophylin (**6**), (**E**) bafilomycin A1 (**8**), and (**F**) isoniazid.

Time-kill experiments were conducted to evaluate the activity of the five most potent compounds, dihydromaltophilin (**1**), L-O-Lac-L-Val-D-O-Hiv-D-Val (**4**), borrelidin (**5**), elaiophylin (**6**), and bafilomycin A1 (**8**), against BCG (Figure 8). Three concentrations of each compound (0.5 × MIC, 1 × MIC, and 2 × MIC) were tested alongside a DMSO control over seven time points from 0 to 108 h, with measurements taken every 18 h. At a 0.5 × MIC dose, all compounds, including the positive control isoniazid, showed only slight inhibition of mycobacterial growth compared to the drug-free control. At a 1 × MIC dose, dihydromaltophilin (**1**) was the only compound unable to achieve full inhibition (100% bacterial kill) by the 108 h mark (Figure 8A). In contrast, bafilomycin A1 (**8**) and O-Lac-L-Val-D-O-Hiv-D-Val (**4**) achieved full inhibition at 108 h (Figure 8B and Figure 8E, respectively), and borrelidin (**5**) achieved full inhibition at 90 h incubation (Figure 8C). Notably, elaiophylin (**6**) demonstrated the fastest bactericidal activity, achieving full inhibition at 72 h (Figure 8D), comparable to the positive control isoniazid (Figure 8F). At a 2 × MIC dose, all tested compounds fully inhibited bacterial growth, confirming their potent bactericidal effects and indicating their exceptional bactericidal potency and rapid action against BCG.

## 3. Discussion

Variation of cultivation condition is a simple and effective strategy for microbial biosynthetic gene cluster activation. The combination of fractionation, NMR fingerprinting, PCA data analysis, and bioactivity evaluation approaches allowed the rapid isolation of compounds **1**–**11**, all of which showed anti-BCG activity, indicating the success of the combined platform. Building on this success, the platform can be further enhanced to unlock even greater possibilities. First, incorporating dereplication into the workflow could significantly improve its ability to identify potentially novel structures. For instance, integrating global natural products social molecular networking (GNPS) [48] analysis with MS/MS data could enable rapid dereplication, making the platform more effective in uncovering new and bioactive natural products in future studies. Second, automating or semi-automating the workflow would greatly improve its speed and scalability, thereby increasing its efficiency and applicability.

The most potent compound, borrelidin (**5**), was first isolated from *S. rochei* and since then has drawn significant attention for its diverse biological activities [49]. Borrelidin is best known for its ability to inhibit threonyl-tRNA synthetase, an essential enzyme for protein synthesis, which makes it an effective agent against various bacterial pathogens [50]. Beyond its antibacterial activity, borrelidin has demonstrated cytotoxicity against cancer cells [51], antimalarial properties [52], anti-angiogenic properties [53], and potential antiviral effects [54], highlighting its versatility as a bioactive molecule. In the context of tuberculosis, borrelidin’s potent anti-mycobacterial activity has been increasingly recognized, showing promise as a candidate for addressing drug-resistant *Mtb* strains [55]. Its ability to achieve bactericidal effects at relatively low concentrations suggests a strong potential for therapeutic development. However, its clinical application can be limited by toxicity concerns, emphasizing the need for further optimization and investigation into its mechanism of action, efficacy, and safety profile against TB pathogens.

This study has identified two potent and broad-spectrum antimicrobial compounds: the peptide L-O-Lac-L-Val-D-O-Hiv-D-Val (**4**) and the polyketide elaiophylin (**6**). Both compounds demonstrated strong and comparable inhibitory activity against a range of microbial pathogens, including BCG, SA, MRSA, and BS. L-O-Lac-L-Val-D-O-Hiv-D-Val (**4**) was originally isolated from a marine-derived *S. bacillaris* [36], but interestingly, this study identified it from an endophyte associated with a TCM, *Cirsium shansiense,* highlighting the evolutionary and ecological links between marine and plant-associated microbial communities. The identification of L-O-Lac-L-Val-D-O-Hiv-D-Val (**4**) in this study also marks the first report of its antimicrobial activity. This novel discovery expands the potential applications of this peptide and reinforces the value of exploring underexploited microbial niches, such as endophytes from medicinal plants, for drug discovery.

Elaiophylin (**6**), a polyketide macrolide, is a member of the elaiophylin family known for its diverse biological activities and antimicrobial potential [56].In this study, elaiophylin demonstrated exceptional antimicrobial potency, achieving rapid bactericidal effects against multiple pathogens. Its ability to achieve full inhibition of BCG growth at 72 h at 1× MIC, and as early as 54 h at 2× MIC, underscores its effectiveness against both slow- and fast-growing bacteria, placing it among the most promising compounds identified in this investigation. Elaiophylin’s potency against drug-resistant bacteria like MRSA highlights its relevance in the search for new antibiotics to combat multidrug-resistant infections. Mechanistic studies of elaiophylin suggest it may act as an effective inhibitor of bacterial efflux pumps, a highly promising target for overcoming bacterial resistance [57]. Efflux pump inhibitors, when used in combination with antibiotics, enhance the efficacy of these drugs by blocking active efflux mechanisms, making resistant bacteria more susceptible [58]. Beyond antimicrobial activity, members of the elaiophylin family have also been noted for their anti-cancer [59], antimalarial [60], and immunomodulatory properties [61], suggesting that elaiophylin may hold therapeutic potential beyond its bactericidal effects. One challenge associated with elaiophylin and derivatives is their reported cytotoxicity, which necessitates further studies to assess their safety profile and therapeutic index.

Polycyclic tetramate macrolactams (PTMs) are a widely distributed class of compounds, consisting of a macrocyclic lactam and a tetramic acid ring. Many PTMs have been reported to display anti-fungal activity [32,34,62,63,64,65], especially for dihydromaltophilin (**1**), which has been widely studied as a biocontrol agent for the fungal infestations of agricultural crops [66]. Additionally, PTMs have been reported to have various bioactivities, such as anticancer, antibacterial, and antiprotozoal [31,67,68]. This study marks the first report of anti-mycobacterial activity for PTM compounds, with MIC values ranging from 3.125 to 100 μg/mL against *M. bovis* BCG for dihydromaltophilin (**1**), xanthobaccin A (**2**), and xanthobaccin C (**3**). As compounds **1**–**3** share the same ring system, the differences in their anti-BCG activities might be ascribed to the substitutions at C-14 and C-25, which would provide prospective directions for bioactivity improvement of the PTM analogs. The new discovery of anti-TB activity of PTMs would provide a new path for the practical application of PTMs in TB drug discovery.

Bafilomycins are a family of macrolide antibiotics produced by various *Streptomyces* species, known for their distinctive 16-membered lactone ring structures and broad-spectrum biological activities, such as antitumor [47,69,70,71], antibacterial [46], antifungal [46,72], antiparasitic [73], and immunosuppressant activities [74]. Among the bafilomycins, bafilomycin A1 is the most extensively studied, renowned for its role as a specific inhibitor of vacuolar H+-ATPase (V-ATPase), which disrupts intracellular pH regulation and impacts essential cellular processes in bacteria, fungi, and cancer cells [75]. Bedaquiline is frequently used at high concentrations to block the fusion between autophagosomes and lysosomes and is an inhibitor of lysosomal degradation [76]. Autophagy plays a critical role in the host immune response against *Mtb*, and therefore the development of autophagy-based therapies represents a promising strategy for anti-TB drug discovery [77,78]. This unique mechanism makes bafilomycins particularly valuable as tools for research and potential therapeutic applications. In this study, bafilomycin derivatives, including bafilomycin A1 (**8**), bafilomycin G (**9**), bafilomycin H (**10**), and bafilomycin D (**11**), demonstrated potent activity against BCG, achieving rapid bactericidal effects at relatively low MIC values. This study highlights the potential of bafilomycins as scaffolds for next-generation anti-TB agents and emphasizes the importance of further exploration into their mechanisms of action and potential synergistic effects in combination therapies.

## 4. Materials and Methods

### 4.1. General Procedures

NMR spectra were recorded in DMSO-*d*_6_ (*δ*_H_ 2.50 and *δ*_C_ 39.5) at 25 °C on a Bruker Avance HDX 800 MHz spectrometer equipped with a TCI cryoprobe (Karlsruhe, Germany), or on a Varian INOVA 600 MHz spectrometer equipped with a triple-resonance cold probe (Billerica, MA, USA). Fractionation of the crude extracts was performed using a Phenomenex C18 Monolithic column (5 μm, 4.6 × 100 mm, Torrance, CA, USA) on a HPLC system, including a Waters 600 pump (Milford, MA, USA) fitted with a 996 photodiode array detector and Gilson FC204 fraction collector (Middleton, WI, USA). Compound purification was conducted using two Thermo Hypersil Gold C18 columns (5 μm, 21.2 × 250 mm and 5 μm, 10 × 250 mm) on a Thermo Ultimate 3000 (Waltham, MA, USA) with a PDA detector. High-resolution mass spectra (HRMS) were recorded on a Bruker maXis II ETD ESI-qTOF (Karlsruhe, Germany). Optical rotations were recorded on a JASCO P-1020 polarimeter (10 cm cell, Tokyo, Japan). All solvents used for extraction, chromatography, [α]_D_, and MS were HPLC grade, and H_2_O was Millipore Milli-Q (Rahway, NJ, USA) PF filtered.

### 4.2. Strain Identification

Three marine strains, MS110104, MS110167, and MS110105, were isolated from a sediment sample collected at a depth of 60 m from the South China Sea, China. The endophyte ES120127 was isolated from TCM *Cirsium shansiense*, which was originally collected from Yunnan Province, China. The land strain, LS120167, was isolated from a desert sample collected from the Taklimakan Desert.

The identification of each strain was performed based on the morphological and 16S ribosomal DNA (rDNA) analyses. Multiple sequence alignments with 16S sequences of related species were carried out using CLUSTAL W [79]. A phylogenetic tree was constructed using the neighbor-joining method [68], as implemented in MEGA 5.0 [80]. Bootstrap values were generated by resampling 1000 replicates. The nucleotide sequences of the 16S rRNA gene have been deposited in GenBank (MS110167: MW485629; MS110104: MW485630; MS110105: PQ626780, ES120127: PP320412, LS120167: PQ626778). The voucher specimen has been deposited at Dr. Zhang’s Laboratory, East China University of Science and Technology (strain nos. MS110104, MS110167, MS110105, ES120127, and LS120167).

### 4.3. Fermentation and Extraction

Small-scaled fermentation for generating the 2562 crude extracts was conducted by culturing the strains on ISP2 agar plates at 28 °C for 7 days. A 250 mL Erlenmeyer flask pre-culture of each strain, containing 40 mL of ISP2 liquid medium, was inoculated with pieces of well-grown agar cultures of the strain at 28 °C (220 rpm) for 48 h. The pre-culture was used to inoculate in a 250 mL Erlenmeyer flask, each with 5 mL of pre-culture and 40 mL ISP2 liquid medium, and incubated at 28 °C (220 rpm) for 3 days to obtain seed cultures for fermentation. Each of the seed cultures was aseptically transferred to 250 mL Erlenmeyer flasks containing 40 mL of different fermentation media (each land and marine strain was cultured in three media, NM2, AM2, and MPG, while the endophyte strains were cultured in six media: NM2, AM2, M001, M12, and M21), and was harvested for 7 days incubation at 28 °C 220 rpm. The broth of each strain was then transferred to a 50 mL tube and incubated at 28 °C 220 rpm for another 2 h with the addition of 5 g HP20 resin. Each broth was centrifuged at 8000 rpm for 5 min to remove the supernatant. The remaining mycelial cake with resin was freeze-dried for 2 days to remove residual moisture, followed by extraction with 10 mL of methanol, with 2 h of shaking and centrifugation at 8000 rpm for 5 min to collect the supernatant. The collected supernatant was evenly distributed into three 96-well deep-well plates. From two of these plates, 100 µL aliquots were transferred into two 96-well plates for activity testing. The remaining crude extracts were dried under nitrogen and stored at −20 °C.

Small-scale fermentation for the five selected strains was cultivated on an ISP2 agar plate at 28 °C for 7 days. A 250 mL Erlenmeyer flask containing 40 mL of ISP2 liquid medium was inoculated with each strain and incubated at 28 °C (220 rpm) for 48 h. Aliquots (2 mL) of the pre-culture were used to inoculate 4 × 250 mL Erlenmeyer flasks, each containing 40 mL of ISP2 liquid medium, and the flasks were incubated at 28 °C (220 rpm) for 3 days. Aliquots (2 mL) of the seed cultures were transferred to 2 × 250 mL Erlenmeyer flasks, each containing 40 mL of media (various media for media various conditions, AM2 for stress conditions, Table 3); inducer molecules (L-homoserine lactone hydrochloride and N-carbobenzoxy-L-homoserine lactone) were sterilized by a 0.22 micron membrane (Sigma-Aldrich, Saint Louis, MO, USA) and added to each culture flask (inducer condition) at a concentration of 200 nM. All flasks were incubated at 28 °C (or specific temperatures for heat shock conditions), 220 rpm for 7 days (or 14 days for half of the media variation conditions). The broths were combined and centrifuged using an Eppendorf centrifuge 5430R (Hamburg, Germany) to yield a supernatant and a cell pellet of each condition. Supernatant samples were dissolved in 750 mL water and extracted three times with 750 mL n-butanol to give supernatant crude extracts. Pellet samples were extracted three times with 500 mL acetone to give cell pellet crude extracts.

Larger-scale fermentation for the five selected strains followed the exact same procedure as described above, with an increase in the number of flasks to 100 for each strain, each containing 40 mL of culture, resulting in a total of 4 L of culture per strain.

### 4.4. Media

ISP-2 (1 L): 4 g yeast extract, 10 g malt extract, 4 g dextrose, 20 g agar, pH 7.2

AM2 (1 L): soluble starch 5 g, glucose 20 g, soybean meal 10 g, peptone 2 g, yeast extract 2 g, NaCl 4 g, K_2_HPO_4_ 0.5 g, MgSO_4_·7H_2_O 0.5 g, CaCO_3_ 2 g, pH 7.3

NM2 (1 L): glucose 1 g, lactose 10 g, glycerol 20 mL, soy peptone 5 g, NH_4_NO_3_ 1.5 g, yeast extract 1 g, trace element 0.2 mL/L, pH 6.0

MPG (1 L): glucose 10 g, millet powder 20 g, soybean meal 20 g, Mops 20 g, pH 7.0

M001 (1 L): starch 20 g, peptone 4 g, yeast extract 8 g, CaCO_3_ 1 g; pH 7.2 was adjusted prior to sterilization.

M12 (1 L): peptone 5 g, yeast extract 1 g, sodium citrate 0.2 g, NaCl 19.45 g, MnCl_2_ 5.9 g, MgSO_4_ 3.24 g, CaCl_2_ 1.8 g, KCl 0.55 g, NaHCO_3_ 0.5 g, KBr 0.08 g, SrCl_2_ 0.034 g, H_3_BO_3_ 0.022 g, Na_2_SiO_3_·9H_2_O 0.004 g, NaF 0.002 g, NH_4_NO_3_ 0.0016 g, Na_2_PO_4_ 0.008 g, pH 7.2

M21 (1 L): glucose 5 g, lactose 40 g, soybean meal 30 g, peptone 5 g, K_2_HPO_4_ 0.5 g, MgSO_4_ 0.5 g, KCl 0.3 g, PH 7.0

Medium 1: (1 L): 20.0 g of D-mannitol, 20.0 g of D-glucose, 5 g of yeast extract, 10.0 g of peptone, 0.5 g of KH_2_PO_4_, 0.3 g of MgSO_4_, 1 g of corn syrup

Medium 2 (1 L): 3 g of sucrose, 0.3 g of NaNO_3_, 0.1 g of K_2_HPO_4_, 0.05 g of KCl, and 0.001 g of FeSO_4_, 0.4 g MgCl_2_

Medium 3 (1 L): glucose 60 g, yeast extract 2 g, (NH_4_)_2_SO_4_ 2 g, MgSO_4_·7H_2_O 0.1 g, K_2_HPO_4_ 0.5 g, NaCl 2 g, FeSO_4_·7H_2_O 0.05 g, ZnSO_4_·7H_2_O 0.05 g, MnSO_4_·4H_2_O 0.05 g, CaCO_3_ 5 g, pH 7.0

Medium 4 (1 L): 24 g soluble starch, 30 g meat extract, 5 g tryptose, 5 g yeast extract, 1 g glucose and 2 g calcium carbonate, pH 7.4

Medium 5 (1 L): soluble starch 20.0 g; glucose 10.0 g; peptone 5.0 g; yeast extract 5.0 g; NaCl 4.0 g; K_2_HPO_4_ 0.5 g; MgSO_4_·7H_2_O, 0.5 g; CaCO_3_ 2.0 g

Medium 6 (1 L): 24 g starch, 1 g glucose, 3 g peptone, 3 g meat extract, 5 g yeast extract, 4 g CaCO_3_, trace metals 5 mL, pH 7.0

Medium 7 (1 L): 10 g glucose, 40 g dextrin, 25 g Bactosoytone, 1 g yeast extract, 3 g CaCO_3_, pH 7.0

Medium 8: glucose 6 g; yeast extract 4 g; pH 7.0

Medium 9: 2 g KH_2_PO_4_, 1.5 g NH_4_Cl, 0.5 g MgSO_4_·7H_2_O, 0.5 g NaCl, 10 g glycerol, 0.4 g myoinositol, 5 g monosodium L-glutamate monohydrate, 0.084 g NaF, 0.025 g FeSO_4_·7H_2_O, 0.01 g ZnSO_4_·7H_2_O, 0.01 g CoCl_2_·6H_2_O, 0.25 g CaCO_3_, 0.001 g *p*-aminobenzoic acid, pH 7.0

Medium 10 (1 L): 10 g glucose, 40 g soluble amylum, 5 g yeast extract, 25 g soybean powder, 5 g peptone, 2 g CaCO_3_, 8 g MgSO_4_·7H_2_O, 6 g FeSO_4_·7H_2_O, 2 g ZnSO_4_·7H_2_O, 2 g MnSO_4_·H_2_O, 0.5 g CoCl_2_·6H_2_O, 2 g Na_2_MoO_4_·2H_2_O, pH 7.0

Trace Salts Solution (100 mL): FeSO_4_·7H_2_O 0.1 g; MnCl_2_·4H_2_O 0.1 g; ZnSO_4_·7H_2_O 0.1 g

### 4.5. HPLC Fractionation Procedure

The fraction library was generated following a previously described protocol [21]. A small amount of dried sample (10 mg) was suspended in 300 µL of DMSO, and 100 µL of the extract was fractionated using HPLC with a C18 Phenomenex Onyx monolithic analytical column (100 × 4.6 mm). The solvent conditions involved a linear gradient starting from 90% H_2_O (0.1% TFA)/10% MeOH (0.1% TFA) to 50% H_2_O (0.1% TFA)/50% MeOH (0.1% TFA) over 3 min at a flow rate of 4 mL/min, followed by a convex gradient to 100% MeOH (0.1% TFA) over 3.5 min at 3 mL/min. This was held at 100% MeOH (0.1% TFA) for 0.5 min at 3 mL/min and an additional 1 min at 4 mL/min. A linear gradient then returned to 90% H_2_O (0.1% TFA)/10% MeOH (0.1% TFA) over 1 min at 4 mL/min, followed by a final hold at 90% H_2_O (0.1% TFA)/10% MeOH (0.1% TFA) for 2 min at 4 mL/min. This cycle prepared the system for the next injection. The total run time for each injection was 11 min, during which time five fractions were collected between 2.0 and 7.0 min: Fraction 1, 2.01–3.00 min; Fraction 2, 3.01–4.00 min; Fraction 3, 4.01–5.00 min; Fraction 4, 5.01–6.00 min; Fraction 5, 6.01–7.00 min.

### 4.6. NMR Fingerprinting

Three combined replicates of each of the five fractions were analyzed using ^1^H NMR spectroscopy. The samples were dissolved in 600 µL of DMSO-*d*_6_ and run in 5 mm NMR tubes. For each sample, the following parameters were applied: pulse width (pw) = 30°, pulse delay (p1) = 9.250 ms, relaxation delay (d2) = 0.4 s, delay (d1) = 1 s, acquisition time (at) = 2.04 s, spectral width (sw) = 20.03 ppm, and number of transients (nt) = 128 scans.

### 4.7. NMR Data Processing and Multivariate Analysis

The spectra were manually phase- and baseline-corrected using Topspin software (v.3.6, Bruker Biospin, Karlsruhe, Germany). The ^1^H NMR spectra were then automatically exported as ASCII files using AMIX (v.3.7, Bruker Biospin, Karlsruhe, Germany). Spectral intensities were normalized to the largest peak (*δ*_H_ = 2.50 ppm) and reduced to integrated regions of equal width (0.02 ppm) across the range of 0.00–15.00 ppm. The region between 2.4 and 3.5 ppm, containing solvent signals from DMSO and water, was excluded from the analysis. Once a bucket table was generated, PCA was performed using the Bruker AMIX software. Because the variables (NMR chemical shifts) were comparable, “No scaling” was applied to preserve natural intensity differences and to emphasize dominant effects. To exclude columns with minimal variance that would have negligible influence on PCA, a minimum variance threshold of 5% of the maximum variance was set. Variables below this threshold were removed from the PCA calculation. A confidence level of 95% was applied to generate the score and loading plots.

### 4.8. Anti-BCG Assay

The *M. bovis* BCG 1173P2 strain used in this assay was transformed with a green fluorescent protein (GFP) constitutive expression plasmid, pUV3583c, enabling direct fluorescence-based measurement of bacterial growth. BCG cultures were grown at 37 °C to mid-log phase in Middlebrook 7H9 broth (Becton Dickinson) supplemented with 10% OADC enrichment (Becton Dickinson), 0.05% Tween-80, and 0.2% glycerol. The culture was then adjusted to an OD_600_ of 0.025 using the same medium to prepare the bacterial suspension. Aliquots of 80 µL of the bacterial suspension were added to each well of a 96-well clear flat-bottom microplate, followed by the addition of 2 µL of compounds dissolved in DMSO, serially two-fold diluted. Isoniazid served as the positive control, while DMSO was used as the negative control. The plate was incubated at 37 °C for 3 days, and GFP fluorescence was measured using a multi-label plate reader in bottom-read mode, with excitation at 485 nm and emission at 535 nm. The minimum inhibitory concentration (MIC) was defined as the lowest drug concentration that inhibited more than 90% of bacterial growth, as determined by the fluorescence intensity.

Each crude extract in the 96-well plate, dried from a 100 μL methanol extract, was reconstituted with 100 μL of DMSO. For the anti-BCG activity test, 2 μL of each well (1× concentration) was tested. Extracts showing activity at the 1× dose were further tested using a serial dilution (8 concentrations) to determine activity.

Each fraction was tested at a maximum concentration of 2.5 μgE/μL (1×), derived from a stock solution of 100 μgE/μL. The concentration units (μgE/μL) are based on two factors: (i) the amount of dry crude extract weighed for fractionation and (ii) the volume of DMSO used to dissolve the crude extract. For example, when 10 mg crude extract is dissolved in 0.3 mL of DMSO, the resulting stock solution concentration is 33.3 μgE/μL. Since three replicates were combined to generate each fraction, the fraction concentration becomes 100 μgE/μL. In the BCG assay, a 40-fold dilution occurs for each sample (2 μL of compound added to 80 μL of culture per well), resulting in a starting test concentration of 2.5 μgE/μL for each fraction.

### 4.9. Anti-Mtb Assay

The *Mycobacterium tuberculosis* strain mc^2^6230 and H_37_Rv used in this study were obtained from the Howard Hughes Medical Institute, Department of Microbiology and Immunology, Albert Einstein College of Medicine. The strain was cultured in Middlebrook 7H9 medium (Difco, Sparks, MD, USA) supplemented with 10% (*v*/*v*) OADC enrichment (Difco), 0.2% (*v*/*v*) glycerol, 0.05% (*v*/*v*) tyloxapol, and pantothenate (50 mg/L). Cultures were grown at 37 °C with shaking at 160 rpm. For the assay, two-fold serial dilutions (eight steps) of each compound were prepared in 100 µL of 7H9 medium in clear-bottomed 96-well plates (Nunc). The last column of each plate, containing no compound, served as the negative control. Previously prepared *Mtb* inocula were diluted in the same medium to achieve an OD_600_ of 0.05 and added to each well. Plates were incubated at 37 °C for five days. Following incubation, 30 µL of 0.02% resazurin was added to each well, and plates were incubated for an additional 24 h at 37 °C. The minimum inhibitory concentration (MIC) was defined as the lowest compound concentration where the well remained blue, indicating no bacterial growth (no color change to pink). All experiments were performed in triplicate.

### 4.10. Antimicrobial Assay

Antimicrobial assays were conducted following the Antimicrobial Susceptibility Testing Standards outlined by the Clinical and Laboratory Standards Institute (CLSI) (NCCLS 1999) using *Staphylococcus aureus* (ATCC 6538), *Bacillus subtilis* (ATCC 6633), methicillin-resistant *S. aureus* (MRSA), and *Pseudomonas aeruginosa* (PAO1). For each organism, a loopful of glycerol stock was streaked onto an LB agar plate and incubated overnight at 37 °C. A single bacterial colony was picked and suspended in Mueller–Hinton broth to achieve a final concentration of approximately 1 × 10^4^ CFU/mL. A two-fold serial dilution of each sample (ranging from 4000 to 31.25 µg/mL in DMSO) was prepared. Aliquots of 2 µL from each dilution were transferred to a 96-well flat-bottom microtiter plate (Greiner). Vancomycin and ciprofloxacin were used as positive controls, and DMSO served as the negative control. Subsequently, 78 µL of the bacterial suspension was added to each well, resulting in final compound concentrations ranging from 100 to 0.78 µg/mL in 2.5% DMSO. The plates were incubated aerobically at 37 °C for 16 h. Following incubation, the optical density at 600 nm OD_600_ for each well was measured using an EnVision 2103 Multi-label Plate Reader (Perkin-Elmer Life Sciences). The minimum inhibitory concentration (MIC) was defined as the lowest compound concentration that inhibited visible bacterial growth. All experiments were performed in triplicate.

### 4.11. Time-Kill Assay

Time-kill analyses were conducted following the CLSI method M26-A.20 (1999). BCG 1173P2 was cultured for 6 days at 37 °C in 7H9 broth. The cells were then diluted in the medium to an initial OD_600_ of 0.025 to prepare the bacterial suspension. The compounds (final concentrations of 0.5, 1, and 2× MIC), or DMSO (drug free control) were added to the suspension. Aliquots of 80 µL were removed at 0, 18, 36, 54, 72, 90, and 108 h of incubation. GFP fluorescence was measured using a multi-label plate reader in bottom-read mode, with excitation at 485 nm and emission at 535 nm. Killing rates were determined by measuring the reduction in GFP fluorescence of viable bacteria log_10_RFU/mL at each time point for fixed concentrations of the compounds. All experiments were performed in triplicate.

### 4.12. Compounds ***1***–***11***

Dihydromaltophilin (**1**): colorless powder, ${\left[\alpha \right]}_{D}^{25}$ +47 (*c* 0.032, MeOH); UV (MeOH) *λ*_max_ (log *ε*) 229 nm (3.42), 328 nm (3.37); ^1^H NMR (800 MHz, DMSO-*d*_6_) *δ*_H_ 8.90 (1H, s, 22-NH), 7.96 (1H, t, J = 6.0 Hz, 28-NH), 6.86 (1H, d, J = 16.2 Hz, H-18), 6.58 (1H, dd, J = 10.0, 15.0 Hz, H-17), 5.01 (1H, td, J = 2.0, 11.0, H-3), 5.71 (1H, dd, J = 2.0, 11.2, H-2), 5.10 (1H, s, 25-OH), 4.47 (1H, s, 14-OH), 3.86 (1H, m, H-23), 3.81 (1H, m, H-25), 3.53 (2H, m, H-4), 3.34 (1H, m, H-14), 3.26 (1H, td, J = 4.0, 9.2 Hz, H-27a), 2.58 (1H, m, H-27b), 2.43 (1H, m, H-8), 2.05 (1H, m, H-16), 2.02 (1H, m, H-9a), 1.97 (1H, m, H-7a), 1.90 (1H, dd, J =2.1, 17.0 Hz, H-7b), 1.76 (1H, m, H-12), 1.75 (1H, m, H-15a), 1.64 (1H, m, H-6), 1.56 (1H, m, H-29a), 1.40 (1H, t, J = 13.6 Hz, H-26a), 1.33 (1H, m, H-10), 1.28 (1H, m, H-5), 1.27 (1H, m, H-11), 1.26 (1H, m, H-15b), 1.20 (1H, m, H-26b), 1.10 (1H, m, H-13), 1.06 (3H, d, J = 6.5, H-31), 1.04 (1H, m, H-29b), 0.87 (1H, m, H-7b), 0.86 (3H, t, J = 7.2, H-30), 0.81 (1H, m, H-9b); ^13^C NMR data (200 MHz, DMSO-*d*_6_) *δ*_C_ 193.5 (C, C24), 176.3 (C, C21), 172.6 (C, C19), 165.7 (C, C1), 150.5 (CH, C17), 139.5 (CH, C3), 124.6 (CH, C2), 121.6 (CH, C18), 103.0 (C, C20), 75.2 (CH, C14), 70.5 (CH, C25), 69.1 (CH, C23), 59.6 (CH, C13), 58.7 (CH, C12), 54.1 (CH, C10), 48.1 (CH, C6), 46.8 (CH, C11), 46.3 (CH, C16), 44.1 (CH, C5), 43.3 (CH, C8), 42.4 (CH_2_, C15), 40.7 (CH_2_, C9), 37.9 (CH_2_, C7), 36.7 (CH_2_, C27), 31.6 (CH_2_, C26), 28.5 (CH_2_, C4), 26.4 (CH_2_, C29), 19.0 (CH_3_, C31), 13.3 (CH_3_, C30). HRMS *m*/*z* 513.2935 [M + H]^+^, calcd. for 513.2959.

Xanthobaccin A (**2**): colorless powder, ${\left[\alpha \right]}_{D}^{25}$ +27 (*c* 0.032, MeOH); UV (MeOH) *λ*_max_ (log *ε*) 227 nm (3.27), 328 nm (3.15); ^1^H NMR (800 MHz, DMSO-*d*_6_) *δ*_H_ 8.95 (1H, s, 22-NH), 8.00 (1H, t, J = 6.0 Hz, 28-NH), 6.84 (1H, d, J = 16.4 Hz, H-18), 6.67 (1H, dd, J = 10.4, 15.2 Hz, H-17), 5.89 (1H, td, J = 2.0, 11.4, H-3), 5.75 (1H, dd, J = 2.0, 11.0, H-2), 5.08 (1H, s, 25-OH), 3.86 (1H, m, H-25), 3.82 (1H, m, H-23), 3.50 (1H, m, H-4), 3.21 (1H, m, H-27a), 2.55 (1H, m, H-27b), 2.46 (1H, m, H-8), 2.41 (1H, m, H-12), 2.25 (1H, m, H-15a), 2.23 (1H, m, H-16), 2.10 (1H, m, H-13), 2.06 (1H, m, H-15b), 2.00 (1H, m, H-7a), 1.95 (1H, m, H-9a), 1.90 (1H, m, H-4b), 1.63 (1H, m, H-6), 1.50 (1H, m, H-29a), 1.42 (1H, m, H-26a), 1.27 (1H, m, H-10), 1.25 (1H, m, H-26b), 1.24 (1H, m, H-5), 1.24 (1H, m, H-11), 0.97 (3H, d, J = 6.5, H-31), 0.95 (1H, m, H-29b), 0.90 (1H, m, H-7b), 0.81 (3H, t, J = 7.2, H-30), 0.80 (1H, m, H-9b); ^13^C NMR data (200 MHz, DMSO-*d*_6_) *δ*_C_ 207.6 (C, C14), 194.5 (C, C24), 178.1 (C, C21), 172.9 (C, C19), 167.0 (C, C1), 150.4 (CH, C17), 138.6 (CH, C3), 125.5 (CH, C2), 121.9 (CH, C18), 102.9 (C, C20), 71.6 (CH, C25), 69.1 (CH, C23), 63.9 (CH, C13), 53.8 (CH, C10), 50.9 (CH, C12), 49.0 (CH_2_, C6), 47.4 (CH, C16), 47.4 (CH, C11), 47.2 (CH_2_, C15), 44.3 (CH, C5), 43.1 (CH, C8), 40.4 (CH_2_, C9), 37.8 (CH, C6), 37.6 (CH_2_, C27), 32.3 (CH_2_, C26), 28.5 (CH_2_, C4), 26.1 (CH_2_, C29), 18.9 (CH_3_, C31), 12.7 (CH_3_, C30). HRMS *m*/*z* 511.2809 [M + H]^+^, calcd. for 511.2803.

Xanthobaccin C (**3**): colorless powder, ${\left[\alpha \right]}_{D}^{25}$ +49 (*c* 0.032, MeOH); UV (MeOH) *λ*_max_ (log *ε*) 226 nm (3.17), 328 nm (3.00); ^1^H NMR (800 MHz, DMSO-*d*_6_) *δ*_H_ 8.90 (1H, s, 22-NH), 7.96 (1H, t, J = 6.0 Hz, 28-NH), 6.94 (1H, d, J = 16.0 Hz, H-18), 6.65 (1H, dd, J = 10.4, 15.4 Hz, H-17), 5.92 (1H, td, J = 2.2, 11.2, H-3), 5.71 (1H, dd, J = 2.0, 11.2, H-2), 3.86 (1H, m, H-23), 3.62 (1H, m, H-4a), 3.24 (1H, m, H-27a), 2.56 (1H, m, H-27b), 2.35 (1H, m, H-13), 2.33 (1H, m, H-8), 2.27 (1H, m, H-15a), 2.27 (1H, m, H-16), 2.20 (1H, m, H-12), 2.20 (1H, m, H-25a), 2.10 (1H, m, H-15b), 2.05 (1H, m, H-7a), 2.03 (1H, m, H-9a), 2.01 (1H, m, H-25b), 1.98 (1H, m, H-4b), 1.68 (1H, m, H-6), 1.50 (1H, m, H-29a), 1.36 (1H, m, H-26a), 1.35 (1H, m, H-10), 1.29 (1H, m, H-5), 1.18 (1H, m, H-26b), 1.17 (1H, m, H-11), 0.96 (3H, d, J = 6.5, H-31), 1.00 (1H, m, H-29b), 0.85 (1H, m, H-7b), 0.83 (3H, t, J = 7.2, H-30), 0.82 (1H, m, H-9b); ^13^C NMR data (200 MHz, DMSO-*d*_6_) *δ*_C_ 207.0 (C, C14), 192.6 (C, C24), 175.3 (C, C21), 171.5 (C, C19), 165.1 (C, C1), 147.3 (CH, C17), 137.9 (CH, C3), 123.9 (CH, C2), 121.4 (CH, C18), 103.4 (C, C20), 68.2 (CH, C23), 62.6 (CH, C13), 52.9 (CH, C10), 50.7 (CH, C6), 50.0 (CH_2_, C12), 47.4 (CH, C16), 46.4 (CH, C11), 45.2 (CH_2_, C15), 43.1 (CH, C8), 42.8 (CH, C5), 39.4 (CH_2_, C9), 38.0 (CH_2_, C7), 36.0 (CH_2_, C27), 28.7 (CH_2_, C25), 27.6 (CH_2_, C26), 27.3 (CH_2_, C4), 25.1 (CH_2_, C29), 19.3 (CH_3_, C31), 13.0 (CH_3_, C30). HRMS *m*/*z* 495.2859 [M + H]^+^, calcd. for 495.2853.

L-O-Lac-L-Val-D-O-Hiv-D-Val (**4**): Yellow solid; ${\left[\alpha \right]}_{D}^{25}$ −8 (*c* 0.2, MeOH); UV (MeOH) *λ*_max_ (log *ε*) 202 nm (3.92); ^1^H NMR (600 MHz, DMSO-*d*_6_) *δ*_H_ 8.41 (1H, d, 2-NH), *δ*_H_ 8.34 (1H, d, 12-NH), *δ*_H_ 5.42 (1H, d, H-7), *δ*_H_ 4.27 (1H, dd, H-2), *δ*_H_ 4.13 (1H, dd, H-12), *δ*_H_ 4.06 (1H, q, H-17), *δ*_H_ 2.65 (1H, m, H-3), *δ*_H_ 2.57 (1H, m, H-13), *δ*_H_ 2.50 (1H, m, H-8), *δ*_H_ 1.57 (3H, d, H-18), *δ*_H_ 1.14 (3H, d, H-14), *δ*_H_ 1.09 (3H, d, H-4), *δ*_H_ 1.02 (3H, d, H-10), *δ*_H_ 0.92 (3H, d, H-9), *δ*_H_ 0.86 (3H, d, H-5), *δ*_H_ 0.85 (3H, d, H-15); ^13^C NMR data (150 MHz, DMSO-*d*_6_) *δ*_C_ 172.7 (C, C16), *δ*_C_ 171.8 (C, C11), *δ*_C_ 171.0 (C, C6), *δ*_C_ 170.3 (C, C1), *δ*_C_ 78.7 (CH, C7), *δ*_C_ 70.4 (CH, C17),*δ*_C_ 60.8 (CH, C12),*δ*_C_ 59.7 (CH, C2), *δ*_C_ 30.5 (CH, C8), *δ*_C_ 29.4 (CH_3_, C4), *δ*_C_ 28.3 (CH, C13), *δ*_C_ 28.2 (CH, C3), *δ*_C_ 19.8 (CH_3_, C14), *δ*_C_ 19.7 (CH_3_, C5), *δ*_C_ 19.4 (CH_3_, C15), *δ*_C_ 19.0 (CH_3_, C9), *δ*_C_ 17.3 (CH_3_, C18), *δ*_C_ 16.7 (CH_3_, C10). HRMS *m*/*z* 389.2289 [M + H]^+^, calcd. for 389.2282.

Borrelidin (**5**): Yellow solid; ${\left[\alpha \right]}_{D}^{25}$ −24 (*c* 0.2, MeOH); UV (MeOH) *λ*_max_ (log *ε*) 244 nm (4.13); ^1^H NMR (600 MHz, methanol-*d*_4_) *δ*_H_ 6.92 (1H, d, H-13), *δ*_H_ 6.61 (1H, dd, H-14), *δ*_H_ 6.33 (1H, ddd, H-15), *δ*_H_ 5.00 (1H, d, H-17), *δ*_H_ 4.20 (1H, d, H-11), *δ*_H_ 3.94 (1H, d, H-3), *δ*_H_ 2.69 (1H, p, H-18), *δ*_H_ 2.50–2.63 (2H, m, H-16), *δ*_H_ 2.46 (1H, q, H-22), *δ*_H_ 2.40 (1H, dd, H-2a), *δ*_H_ 2.25 (2H, dd, H-2b), *δ*_H_ 2.01 (2H, m, H-19a), *δ*_H_ 2.01 (2H, m, H-21a), *δ*_H_ 1.83 (1H, m, H-4), *δ*_H_ 1.83 (1H, m, H-6), *δ*_H_ 1.83 (1H, m, H-10), *δ*_H_ 1.83 (2H, m, H-20), *δ*_H_ 1.83 (1H, m, H-21b), *δ*_H_ 1.59–1.71 (2H, m, H-7), *δ*_H_ 1.43 (1H, m, H-19b), *δ*_H_ 1.22 (1H, m, H-5a), *δ*_H_ 1.22 (1H, m, H-9a), *δ*_H_ 1.05 (3H, d, H-27), *δ*_H_ 0.99 (1H, t, H-5b), *δ*_H_ 0.99 (1H, t, H-8), *δ*_H_ 0.87 (3H, d, H-24), *δ*_H_ 0.87 (3H, d, H-25), *δ*_H_ 0.87 (3H, d, H-26), *δ*_H_ 0.72 (1H, t, H-9b); ^13^C NMR data (150 MHz, methanol-*d*_4_) *δ*_C_ 178.8 (C, C23), *δ*_C_ 171.9 (C, C1), *δ*_C_ 144.0 (CH, C13), *δ*_C_ 138.7 (CH, C15), *δ*_C_ 127.6 (CH, C14), *δ*_C_ 118.5 (C, C28), *δ*_C_ 116.1 (C, C12), *δ*_C_ 76.0 (CH, C17), *δ*_C_ 71.6 (CH, C3), *δ*_C_ 71.5 (CH, C11), *δ*_C_ 48.6 (CH, C22), *δ*_C_ 48.0 (CH, C8), *δ*_C_ 46.1 (CH, C18), *δ*_C_ 43.2 (CH_2_, C5), *δ*_C_ 37.7 (CH_2_, C9), *δ*_C_ 36.7 (CH_2_, C2), *δ*_C_ 35.7 (CH, C4), *δ*_C_ 35.3 (CH_2_, C16), *δ*_C_ 34.6 (CH, C10), *δ*_C_ 31.1 (CH_2_, C21), *δ*_C_ 29.1 (CH_2_, C19), *δ*_C_ 27.2 (CH, C6), *δ*_C_ 26.3 (CH_2_, C7), *δ*_C_ 24.8 (CH_2_, C20), *δ*_C_ 19.6 (CH_3_, C25), *δ*_C_ 17.6 (CH_3_, C26), *δ*_C_ 17.4 (CH_3_, C24), *δ*_C_ 14.1 (CH_3_, C27). HRMS *m/z* 512.2986 [M + Na]^+^, calcd. for 512.2983.

Elaiophylin (**6**): Colorless powder; ${\left[\alpha \right]}_{D}^{25}$ −44 (*c* 0.6, MeOH); UV (MeOH) *λ*_max_ (log *ε*) 254 nm (4.42); ^1^H NMR (600 MHz, DMSO-*d*_6_) *δ*_H_ 6.80 (1H, dd, H-3/H-3′), *δ*_H_ 6.09 (1H, dd, H-4/H-4′), *δ*_H_ 5.69 (1H, d, H-2/H-2′), *δ*_H_ 5.63 (1H, dd, H-5/H-5′), *δ*_H_ 5.06 (1H, m, H-7/H-7′), *δ*_H_ 4.90 (1H, m, H-22/H-22′), *δ*_H_ 4.41 (1H, m, H-9/H-9′), *δ*_H_ 3.78 (1H, m, H-26/H-26′), *δ*_H_ 3.77 (1H, m, H-24/H-24′), *δ*_H_ 3.76 (1H, m, H-13/H-13′), *δ*_H_ 3.75 (1H, m, H-15/H-15′), *δ*_H_ 3.71 (1H, m, H-25/H-25′), *δ*_H_ 2.25 (1H, m, H-6/H-6′), *δ*_H_ 2.23 (1H, d, H-12a/H-12′a), *δ*_H_ 1.96 (1H, m, H-8/H-8′), *δ*_H_ 1.80 (1H, m, H-23/H-23′), *δ*_H_ 1.71 (1H, m, H-10/H-10′), *δ*_H_ 1.61 (1H, m, H-20a/H-20′a), *δ*_H_ 1.44 (1H, m, H-20b/H-20′b), *δ*_H_ 1.18 (1H, m, H-14/H-14′), *δ*_H_ 1.06 (3H, d, H-27/H-27′),*δ*_H_ 1.03 (3H, d, H-16/H-16′), *δ*_H_ 0.96 (3H, d, H-17/H-17′), *δ*_H_ 0.95 (1H, m, H-12b/H-12′b), *δ*_H_ 0.84 (3H, d, H-7/H-7′), *δ*_H_ 0.78 (3H, t, H-21/H-21′), *δ*_H_ 0.77 (3H, d, H-18/H-18′); ^13^C NMR data (150 MHz, DMSO-*d*_6_) *δ*_C_ 145.1 (CH, C3/C3′), *δ*_C_ 144.4 (CH, C5/C5′), *δ*_C_ 132.0 (CH, C4/C4′), *δ*_C_ 121.1 (CH, C2/C2′), *δ*_C_ 93.4 (CH, C22/C22′), *δ*_C_ 77.8 (CH, C7/C7′), *δ*_C_ 71.8 (CH, C25/C25′), *δ*_C_ 70.7 (CH, C9/C9′), *δ*_C_ 70.5 (CH, C13/C13′), *δ*_C_ 66.7 (CH, C15/C15′), *δ*_C_ 66.2 (CH, C26/C26′), *δ*_C_ 66.0 (CH, C24/C24′), *δ*_C_ 48.6 (CH, C14/C14′), *δ*_C_ 41.8 (CH, C10/C10′), *δ*_C_ 40.9 (CH, C6/C6′), *δ*_C_ 39.0 (CH_2_, C12/C12′), *δ*_C_ 36.0 (CH, C8/C8′), *δ*_C_ 33.7 (CH_2_, C23/C23′), *δ*_C_ 19.5 (CH_2_, C20/C20′), *δ*_C_ 19.2 (CH_3_, C16/C16′), *δ*_C_ 16.9 (CH_3_, C27/C27′), *δ*_C_ 15.0 (CH_3_, C17/C17′), *δ*_C_ 9.3 (CH_3_, C21/C21′), *δ*_C_ 8.8 (CH_3_, C18/C18′), *δ*_C_ 7.1 (CH_3_, C19/C19′). HRMS *m*/*z* 1025.6049 [M + H]^+^, calcd. for 1025.6043.

Efomycin G (**7**): Colorless powder; ${\left[\alpha \right]}_{D}^{25}$ −45 (*c* 0.6, MeOH); UV (MeOH) *λ*_max_ (log *ε*) 254 nm (4.96); ^1^H NMR (600 MHz, DMSO-*d*_6_) *δ*_H_ 6.79 (1H, dd, H-3/H-3′), *δ*_H_ 6.08 (1H, dd, H-4/H-4′), *δ*_H_ 5.68 (1H, d, H-2/H-2′), *δ*_H_ 5.64 (1H, dd, H-5/H-5′), *δ*_H_ 5.06 (1H, m, H-7/H-7′), *δ*_H_ 4.90 (1H, m, H-22/H-22′), *δ*_H_ 4.11 (1H, m, H-9/H-9′), *δ*_H_ 4.00 (1H, m, H-26/H-26′), *δ*_H_ 4.00 (1H, m, H-24/H-24′), *δ*_H_ 3.97 (1H, m, H-13), *δ*_H_ 3.92 (1H, m, H-15), *δ*_H_ 3.71 (1H, m, H-15′), *δ*_H_ 3.66 (1H, m, H-13′), *δ*_H_ 3.62 (1H, m, H-25/H-25′), *δ*_H_ 2.55 (1H, m, H-6/H-6′), *δ*_H_ 2.39 (1H, dd, H-12a/H-12′a), *δ*_H_ 1.96 (1H, m, H-8/H-8′), *δ*_H_ 1.80 (1H, m, H-23/H-23′), *δ*_H_ 1.72 (1H, m, H-10/H-10′), *δ*_H_ 1.63 (1H, m, H-20a/H-20′a), *δ*_H_ 1.42 (1H, m, H-20b/H-20′b), *δ*_H_ 1.29 (1H, m, H-14′),*δ*_H_ 1.25 (3H, d, H-27/H-27′), *δ*_H_ 1.21 (1H, m, H-14), *δ*_H_ 1.11 (3H, d, H-16′), *δ*_H_ 1.10 (3H, d, H-16), *δ*_H_ 1.04 (1H, d, H-17/H-17′), *δ*_H_ 1.03 (1H, m, H-12b/H-12′b), *δ*_H_ 1.01 (3H, d, H-19/H-19′), *δ*_H_ 0.92 (3H, d, H-20′), *δ*_H_ 0.85 (3H, t, H-21), *δ*_H_ 0.82 (3H, d, H-18/H-18′); ^13^C NMR data (150 MHz, DMSO-*d*_6_) *δ*_C_ 170.0 (C, C1/C1′), *δ*_C_ 145.0 (CH, C3/C3′), *δ*_C_ 144.3 (CH, C5/C5′), *δ*_C_ 132.0 (CH, C4/C4′), *δ*_C_ 121.0 (CH, C2/C2′), *δ*_C_ 99.1 (C, C11), *δ*_C_ 99.0 (C, C11′), *δ*_C_ 93.1 (CH, C22), *δ*_C_ 92.9 (CH, C22′), *δ*_C_ 77.9 (CH, C7/C7′), *δ*_C_ 73.3 (CH, C13′), *δ*_C_ 71.4 (CH, C25/C25′), *δ*_C_ 70.6 (CH, C9/C9′), *δ*_C_ 70.0 (CH, C13), *δ*_C_ 69.3 (CH, C15′), *δ*_C_ 66.5 (CH, C15), *δ*_C_ 66.0 (CH, C26/C26′), *δ*_C_ 65.9 (CH, C24), *δ*_C_ 65.8 (CH, C24′), *δ*_C_ 48.3 (CH, C14), *δ*_C_ 43.5 (CH, C14′), *δ*_C_ 41.6 (CH, C10), *δ*_C_ 41.5 (CH, C10′),*δ*_C_ 40.8 (CH, C6/C6′), *δ*_C_ 38.8 (CH_2_, C12), *δ*_C_ 38.5 (CH_2_, C12′), *δ*_C_ 35.9 (CH, C8/C8′), *δ*_C_ 33.5 (CH_2_, C23), *δ*_C_ 33.4 (CH_2_, C23′), *δ*_C_ 24.9 (CH_2_, C20), *δ*_C_ 19.4 (CH_3_, C16), *δ*_C_ 19.1 (CH_3_, C16′), *δ*_C_ 16.8 (CH_3_, C27), *δ*_C_ 16.7 (CH_3_, C27′), *δ*_C_ 14.9 (CH_3_, C17/C17′), *δ*_C_ 13.4 (CH_2_, C20′), *δ*_C_ 9.0 (CH_3_, C21), *δ*_C_ 8.8 (CH_3_, C18/C18′), *δ*_C_ 7.1 (CH_3_, C19/C19′). HRMS *m/z* 1011.5882 [M + H]^+^, calcd. for 1011.5887.

Bafilomycin A1 (**8**): White powder; ${\left[\alpha \right]}_{D}^{25}$ −24 (*c* 0.3, MeOH); UV (MeOH) *λ*_max_ (log *ε*) 244 nm (4.24), 281 nm (3.96); ^1^H NMR (800 MHz, DMSO-*d*_6_) *δ*_H_ 6.53 (1H, s, H-3), 6.53 (1H, dd, J = 15.7, 10.8 Hz, H-12), 5.83 (1H, d, J = 8.0 Hz, H-5), 5.71 (1H, d, J = 11.4, H-11), 5.33 (1H, d, J = 1.6 Hz, 19-OH), 5.15 (1H, dd, J = 14.4, 8.4 Hz, H-13), 5.13 (1H, dd, J = 1.6, 6.4 Hz, H-15), 4.91 (1H, d, J =5.6 Hz, 7-OH), 4.52 (1H, d, J = 5.6 Hz, 17-OH), 4.46 (1H, d, J = 5.6 Hz, 21-OH), 3.99 (1H, m, H-17), 3.97 (1H, dd, J = 7.2, 8.0 Hz, H-17), 3.54 (3H, s, 2-OCH_3_), 3.36 (1H, m, H-21), 3.32 (1H, m, H-23), 3.17 (1H, m, H-7), 3.16 (3H, s, 14-OCH_3_), 2.42 (1H, m, H-6a), 2.04 (1H, dd, J = 11.2, 13.6 Hz, H-6b), 2.00 (2H, dd, J = 4.8, 12.0, H-20), 1.89 (3H, s, H-26), 1.88 (2H, m, H-9), 1.86 (1H, m, H-16), 1.82 (1H, td, J = 2.4, 7.2 Hz, H-24), 1.79 (3H, s, H-29), 1.72 (1H, m, H-18), 1.60 (1H, dd, J = 1.6, 7.2 Hz, H-8), 1.14 (1H, m, H-22a), 1.07 (1H, td, J = 1.6, 12.0 Hz, H-22b), 0.96 (3H, d, J = 7.2, H-27), 0.89 (3H, d, J = 6.5, H-28), 0.88 (3H, d, J = 3.2, H-31), 0.87 (3H, d, J = 6.4, H-32), 0.83 (3H, d, J = 6.4, H-33), 0.78 (3H, d, J = 7.2, H-30), 0.72 (3H, d, J = 7.2, H-25); ^13^C NMR data (200 MHz, DMSO-*d*_6_) *δ*_C_ 164.9 (C, C1), 144.6 (CH, C5), 143.2 (C, C10), 141.3 (C, C2), 132.6 (CH, C12), 132.3 (CH, C3), 131.1 (C, C4), 126.0 (CH, C13), 124.3 (CH, C11), 99.1 (C, C19), 83.4, (CH, C14), 78.9 (CH, C7), 76.1 (CH, C23), 75.8 (CH, C15), 70.4 (CH, C17), 69.1 (CH, C21), 59.8 (CH_3_, 2-OCH_3_), 55.6 (CH_3_, 14-OCH_3_), 43.3 (CH_2_, C20), 43.0 (CH, C8), 41.7 (CH_2_, C9), 41.2 (CH, C22), 39.9 (CH, C18), 39.0 (CH, C16), 37.7 (CH, C6), 28.0 (CH, C24), 23.1 (CH_3_, C28), 21.5 (CH_3_, C32), 19.7 (CH_3_, C29), 18.4 (CH_3_, C27), 12.8 (CH_3_, C33), 11.0 (CH_3_, C30), 7.4 (CH_3_, C31). HRMS *m*/*z* 621.4002 [M − H]^−^, calcd. for 621.4008.

Bafilomycin G (**9**): White powder; ${\left[\alpha \right]}_{D}^{25}$ −21 (*c* 0.3, MeOH); UV (MeOH) *λ*_max_ (log *ε*) 245 nm (4.42), 280 nm (3.67); ^1^H NMR (800 MHz, DMSO-*d*_6_) *δ*_H_ 6.64 (1H, dd, J = 15.0, 11.0 Hz, H-12), 6.55 (1H, s, H-3), 5.83 (1H, d, J = 9.0 Hz, H-5), 5.76 (1H, d, J = 10.5, H-11), 5.35 (1H, d, J = 1.6 Hz, 19-OH), 5.21 (1H, dd, J = 15.0, 9.5 Hz, H-13), 5.05 (1H, d, J = 9.0 Hz, H-15), 4.91 (1H, d, J = 5.6 Hz, 7-OH), 4.54 (1H, d, J = 6.2 Hz, 17-OH), 4.03 (1H, m, H-17), 3.87 (1H, dd, J = 8.5, 8.0 Hz H-14), 3.57 (1H, m, H-23), 3.56 (3H, s, 2-OCH_3_), 3.51 (3H, s, 21-OCH_3_), 3.20 (1H, m, H-7), 3.16 (3H, s, 14-OCH_3_), 2.94 (1H, m, H-21), 2.44 (1H, m, H-6), 2.19 (1H, m, H-20a), 2.10 (1H, m, H-20b), 2.00 (3H, s, H-26), 1.91 (3H, m, H-29), 1.90 (1H, m, H-24), 1.88 (2H, m, H-9), 1.87 (1H, m, H-16), 1.78 (1H, m, H-18), 1.65 (1H, m, H-8), 1.48 (1H, m, H-22a), 1.14 (1H, m, H-22b), 0.98 (3H, d, J = 7.0, H-27), 0.95 (3H, d, J = 6.5, H-28), 0.88 (3H, d, J = 7.2 Hz, H-31), 0.87 (3H, d, J = 6.8, H-30), 0.84 (3H, d, J = 7.5, H-32), 0.83 (3H, d, J = 7.5, H-33), 0.78 (3H, d, J = 6.7, H-25); ^13^C NMR data (200 MHz, DMSO-*d*_6_) 141.0 (CH, C5), 133.2 (CH, C3), 132.0 (CH, C12), 126.5 (CH, C13), 125.5 (CH, C11), 83.0, (CH, C14), 80.2 (CH, C21), 80.0 (CH, C7), 76.2 (CH, C15), 71.3 (CH, C23), 70.9 (CH, C17), 60.2 (CH_3_, 2-OCH_3_), 57.4 (CH_3_, 21-OCH_3_), 55.8 (CH_3_, 14-OCH_3_), 43.3 (CH_2_, C20), 41.8 (CH, C8), 41.3 (CH_2_, C18), 40.6 (CH_2_, C9), 38.8 (CH, C16), 37.2 (CH, C6), 35.9 (CH, C22), 28.1 (CH, C24), 22.5 (CH_3_, C28), 22.5 (CH_3_, C32), 20.2 (CH_3_, C29), 17.4 (CH_3_, C27), 14.3 (CH_3_, C33), 14.1 (CH_3_, C26), 14.0 (CH_3_, C25), 12.3 (CH_3_, C30), 7.3 (CH_3_, C31). HRMS *m*/*z* 635.4161 [M − H]^−^, calcd. for 635.4165.

Bafilomycin H (**10**): ${\left[\alpha \right]}_{D}^{25}$ −14 (*c* 0.3, MeOH); UV (MeOH) *λ*_max_ (log *ε*) 245 nm (4.47), 280 nm (3.52); ^1^H NMR (800 MHz, DMSO-*d*_6_) *δ*_H_ 6.53 (1H, s, H-3), 6.49 (1H, dd, J = 15.2, 10.6 Hz, H-12), 5.84 (1H, d, J = 9.0 Hz, H-5), 5.73 (1H, d, J = 11.0, H-11), 5.18 (1H, dd, J = 15.0, 9.4 Hz, H-13), 5.15 (1H, dd, J = 8.5, 1.7 Hz, H-15), 4.90 (1H, d, J = 5.8 Hz, 7-OH), 4.51 (1H, d, J = 5.6 Hz, 17-OH), 3.96 (1H, m, H-17), 3.96 (1H, dd, J = 8.2, 8.0 Hz H-14), 3.66 (1H, m, H-23), 3.54 (3H, s, 2-OCH_3_), 3.19 (1H, m, H-7), 3.18 (3H, s, 19-OCH_3_), 3.17 (3H, s, 14-OCH_3_), 3.15 (3H, s, 21-OCH_3_), 2.98 (1H, m, H-21), 2.44 (1H, m, H-6a), 2.12 (1H, m, H-6b), 2.08 (1H, m, H-20a), 1.90 (3H, s, H-26), 1.75 (3H, m, H-29), 1.92 (1H, m, H-24), 1.91 (1H, m, H-16), 1.87 (2H, m, H-9), 1.77 (1H, m, H-18), 1.71 (1H, m, H-8), 1.15 (1H, m, H-22), 1.17 (1H, m, H-20b), 0.98 (3H, d, J = 7.2 Hz, H-31), 0.96 (3H, d, J = 7.2, H-27), 0.94 (3H, d, J = 6.5, H-28), 0.88 (3H, d, J = 6.8, H-30), 0.84 (3H, d, J = 7.4, H-32), 0.83 (3H, d, J = 7.4, H-33), 0.80 (3H, d, J = 6.7, H-25); ^13^C NMR data (200 MHz, DMSO-*d*_6_) 142.1 (CH, C5), 141.6 (C, C2), 132.6 (CH, C3), 132.0 (CH, C12), 125.9 (CH, C13), 124.5 (CH, C11), 83.0, (CH, C14), 79.0 (CH, C7), 78.9 (CH, C21), 75.9 (CH, C15), 72.0 (CH, C17), 72.3 (CH, C23), 60.0 (CH_3_, 2-OCH_3_), 55.8 (CH_3_, 21-OCH_3_), 55.8 (CH_3_, 14-OCH_3_), 48.9 (CH_3_, 19-OCH_3_), 44.5 (CH_2_, C20), 42.6 (CH, C8), 40.6 (CH_2_, C18), 40.5 (CH_2_, C9), 38.3 (CH, C16), 37.6 (CH, C6), 36.2 (CH, C22), 29.0 (CH, C24), 22.5 (CH_3_, C32), 22.1 (CH_3_, C28), 19.7 (CH_3_, C29), 17.4 (CH_3_, C27), 14.2 (CH_3_, C33), 14.2 (CH_3_, C25), 14.1 (CH_3_, C26), 12.2 (CH_3_, C30), 9.4 (CH_3_, C31). HRMS *m*/*z* 649.4317 [M − H]^−^, calcd. for 649.4321.

Bafilomycin D (**11**): ${\left[\alpha \right]}_{D}^{25}$ −17 (*c* 0.3, MeOH); UV (MeOH) *λ*_max_ (log *ε*) 245 nm (4.32), 283 nm (3.26); ^1^H NMR (800 MHz, DMSO-*d*_6_) *δ*_H_ 6.80 (1H, dd, J = 15.7, 8.8 Hz, H-21), 6.53 (1H, s, H-3), 6.49 (1H, dd, J = 15.1, 10.4 Hz, H-12), 6.20 (1H, d, J = 15.7 Hz, H-20), 5.84 (1H, d, J = 9.3, H-5), 5.73 (1H, d, J = 10.4 Hz, H-11), 5.19 (1H, m, H-13), 5.17 (1H, m, H-15), 4.89 (1H, d, J = 6.6 Hz, 7-OH), 4.53 (1H, d, J = 6.6 Hz, 17-OH), 4.44 (1H, d, J = 6.6 Hz, 23-OH), 3.98 (1H, m, H-14), 3.96 (1H, m, H-17), 3.68 (1H, m, H-23), 3.55 (3H, s, 2-OCH_3_), 3.19 (1H, dd, J = 2.3, 6.8 Hz, H-7), 3.15 (3H, s, 14-OCH_3_), 2.99 (1H, m, H-18), 2.53 (1H, m, H-22), 2.45 (1H, m, H-6), 2.11 (1H, m, H-16), 1.90 (1H, m, H-24), 1.90 (1H, m, H-9a), 1.89 (3H, s, H-26), 1.88 (3H, m, H-29), 1.82 (1H, m, H-9b), 1.50 (1H, m, H-8), 0.99 (3H, d, J = 7.0 Hz, H-31), 0.97 (3H, d, J = 7.0, H-32), 0.96 (3H, d, J = 6.8, H-30), 0.90 (3H, d, J = 7.0, H-27), 0.89 (3H, d, J = 7.5, H-33), 0.85 (3H, d, J = 6.5, H-28), 0.81 (3H, d, J = 6.7, H-25); ^13^C NMR data (200 MHz, DMSO-*d*_6_) 147.7 (CH, C21), 143.1 (CH, C5), 132.7 (CH, C12), 132.2 (CH, C3), 127.4 (CH, C20), 125.4 (CH, C11), 83.7 (CH, C23), 82.8 (CH, C14), 80.4 (CH, C7), 75.9 (CH, C15), 72.1 (CH, C17), 60.2 (CH_3_, 2-OCH_3_), 55.8 (CH_3_, 14-OCH_3_), 46.9 (CH, C18), 43.3 (CH, C8), 41.5 (CH_2_, C9), 38.8 (CH, C16), 38.5 (CH, C22), 36.9 (CH, C6), 31.4 (CH, C24), 23.5 (CH_3_, C-28), 20.2 (CH_3_, C-29), 18.4 (CH_3_, C-27), 17.8 (CH_3_, C-25), 15.6 (CH_3_, C-32), 14.3 (CH_3_, C-33), 14.1 (CH_3_, C-26), 12.3 (CH_3_, C-30), 9.5 (CH_3_, C-31). HRMS *m*/*z* 603.3896 [M − H]^−^, calcd. for 603.3897.

## Figures and Tables

**Table 1:** Examples of active microbial natural products identified through the application of various OSMAC strategies.

OSMAC Strategy	Microorganisms	Induced Metabolites	Bioactivity
Heat shock [14]	*Streptomyces* *venezuelae*	* 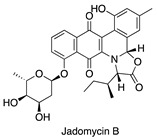 *	Antibacterial
Addition of antibiotics [15]	*Streptomyces* sp.	* 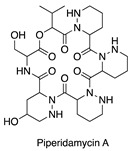 *	Antibacterial
Addition of inducers [16,17]	*S. natalensis*	* 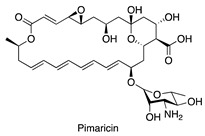 *	Antifungal
Addition of inhibitors [18]	*Phomospis asparagi*	* 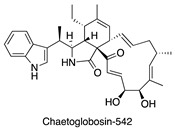 *	Cytotoxic
Co-culture [19]	*Pestalotia* sp.	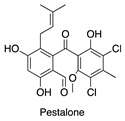	Antibacterial
Media variation [20]	*Bipolaris oryzae*	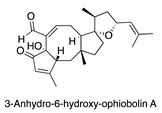	Antibacterial

**Table 2:** Antimicrobial activities of compounds **1**–**11** (μg/mL).

Strains	1	2	3	4	5	6	7	8	9	10	11	Positive Control
*M. bovis* BCG	3.125	25	100	3.125	0.78	1.56	6.25	6.25	6.25	6.25	12.5	0.05 ^a^
*Mtb* mc2 6220	>100	>100	>100	50% at 50 μM	50% at 50 μM	3.125	25	3.89	45% at 50 μM	>100	>100	0.1 ^a^
*Mtb* H37Rv	NT #	NT	NT	NT	NT	>100	>100	>100	NT	NT	NT	0.1 ^a^
*Staphylococcus aureus* (SA)	>100	>100	>100	3.125	100	0.78	1.56	50	50	50	50	1 ^b^
Methicillin-resistant *S. aureus* (MRSA)	>100	>100	>100	12.5	>100	1.56	6.25	>100	>100	>100	>100	1 ^b^
*Bacillus subtilis* (BS)	>100	>100	>100	3.125	25	1.56	6.25	>100	>100	>100	>100	0.5 ^b^
*Pseudomonas aeruginosa* (PA)	>100	>100	>100	>100	>100	>100	>100	>100	>100	>100	>100	1 ^c^

# NT: not tested. a. Isoniazid. b. Vancomycin. c. Ciprofloxacin.

**Table 3:** 32 OSMAC conditions used in this study.

Conditions	Media variation (1–10)	Media variation (11–20)	Heat shock (21–23)	pH shock (24–26)	Ethanol shock (27–29)	Inducer addition (30–31)	Standard (32)
Medium	Media 1–10	Media 1–10	AM2	AM2	AM2	AM2	AM2
Culture length	7 days	14 days	7 days	7 days	7 days	7 days	7 days
pH	7.0–7.4	7.0–7.4	7.3	5.5, 7.5, 9.5	7.3	7.3	7.3
Temperature	28 °C	28 °C	37 °C, 42 °C, or 42 °C for one hour (then 28 °C)	28 °C	28 °C	28 °C	28 °C
Ethanol	0	0	0	0	1 mM, 10 mM, or 100 mM	0	0
Inducers	0	0	0	0	0	200 nM of L-homoserine lactone hydrochloride or N-carbobenzoxy-L-homoserine lactone	0

## Data Availability

The original contributions presented in this study are included in the article/Supplementary Materials. Further inquiries can be directed to the corresponding authors.

## Supplementary Materials

The following supporting information can be downloaded at: www.mdpi.com/article/10.3390/antibiotics14010108/s1, Figure S1: Legend information of the PCA scores plot for Figures S2–S5. Figure S2. PCA Results of 320 Fractions from ES120127. Figure S3. PCA Results of 320 Fractions from LS120167. Figure S4. PCA Results of 320 Fractions from MS110105. Figure S5. PCA Results of 320 Fractions from MS110104. Figure S6. 1H NMR spectrum of **1** (DMSO-d6, 800 MHz). Figure S7. COSY NMR spectrum of **1** (DMSO-d6, 800 MHz). Figure S8. HSQC NMR spectrum of **1** (DMSO-d6, 800 MHz). Figure S9. HMBC NMR spectrum of **1** (DMSO-d6, 800 MHz). Figure S10. HRMS spectrum of **1**. Figure S11. 1H NMR spectrum of **2** (DMSO-d6, 800 MHz). Figure S12. COSY NMR spectrum of **2** (DMSO-d6, 800 MHz). Figure S13. HSQC NMR spectrum of **2** (DMSO-d6, 800 MHz). Figure S14. HMBC NMR spectrum of **2** (DMSO-d6, 800 MHz). Figure S15. HRMS spectrum of **2**. Figure S16. 1H NMR spectrum of **3** (DMSO-d6, 800 MHz). Figure S17. COSY NMR spectrum of **3** (DMSO-d6, 800 MHz). Figure S18. HSQC NMR spectrum of **3** (DMSO-d6, 800 MHz). Figure S19. HMBC NMR spectrum of **3** (DMSO-d6, 800 MHz). Figure S20. HRMS spectrum of **3**. Figure S21. 1H NMR spectrum of **4** (DMSO-d6, 600 MHz). Figure S22. 13C NMR spectrum of **4** (DMSO-d6, 150 MHz). Figure S23. COSY NMR spectrum of **4** (DMSO-d6, 600 MHz). Figure S24. HSQC NMR spectrum of **4** (DMSO-d6, 600 MHz). Figure S25. HMBC NMR spectrum of **4** (DMSO-d6, 600 MHz). Figure S26. HRMS spectrum of **4**. Figure S27. 1H NMR spectrum of **5** (methanol-d4, 600 MHz). Figure S28. 13C NMR spectrum of **5** (methanol-d4, 150 MHz). Figure S29. COSY NMR spectrum of **5** (methanol-d4, 600 MHz). Figure S30. HSQC NMR spectrum of **5** (methanol-d4, 600 MHz). Figure S31. HMBC NMR spectrum of **5** (methanol-d4, 600 MHz). Figure S32. HRMS spectrum of **5**. Figure S33. 1H NMR spectrum of **6** (DMSO-d6, 600 MHz). Figure S34. COSY NMR spectrum of **6** (DMSO-d6, 600 MHz). Figure S35. HSQC NMR spectrum of **6** (DMSO-d6, 600 MHz). Figure S36. HRMS spectrum of **6**. Figure S37. 1H NMR spectrum of **7** (DMSO-d6, 600 MHz). Figure S38. COSY NMR spectrum of **7** (DMSO-d6, 600 MHz). Figure S39. HSQC NMR spectrum of **7** (DMSO-d6, 600 MHz). Figure S40. HRMS spectrum of **7**. Figure S41. 1H NMR spectrum of **8** (DMSO-d6, 800 MHz). Figure S42. 13C NMR spectrum of **8** (DMSO-d6, 200 MHz). Figure S43. COSY NMR spectrum of **8** (DMSO-d6, 800 MHz). Figure S44. HSQC NMR spectrum of **8** (DMSO-d6, 800 MHz). Figure S45. HMBC NMR spectrum of **8** (DMSO-d6, 800 MHz). Figure S46. HRMS spectrum of **8**. Figure S47. 1H NMR spectrum of **9** (DMSO-d6, 800 MHz). Figure S48. COSY NMR spectrum of **9** (DMSO-d6, 800 MHz). Figure S49. HSQC NMR spectrum of **9** (DMSO-d6, 800 MHz). Figure S50. HRMS spectrum of **9**. Figure S51. 1H NMR spectrum of **10** (DMSO-d6, 800 MHz). Figure S52. COSY NMR spectrum of **10** (DMSO-d6, 800 MHz). Figure S53. HSQC NMR spectrum of **10** (DMSO-d6, 800 MHz). Figure S54. HRMS spectrum of **10**. Figure S55. 1H NMR spectrum of **11** (DMSO-d6, 800 MHz). Figure S56. COSY NMR spectrum of **11** (DMSO-d6, 800 MHz). Figure S57. HSQC NMR spectrum of **11** (DMSO-d6, 800 MHz). Figure S58. HRMS spectrum of **11**.
