# Supplementary material for: Nuclear Magnetic Resonance Fingerprinting and Principal Component Analysis Strategies Lead to Anti-Tuberculosis Natural Product Discovery from Actinomycetes
Source: Antibiotics (Basel). 2025 Jan 20;14(1):108. doi: 10.3390/antibiotics14010108 (PMC11763000; doi:10.3390/antibiotics14010108)
Supplement: Supplementary file 1 [file antibiotics-14-00108-s001.zip › antibiotics-3372988-supplementary.pdf]

Nuclear Magnetic Resonance Fingerprinting and Principal Component Analysis Strategies Lead to Anti-Tuberculosis Natural Product Discovery from Actinomycetes

Jianying Han [1,2]*, Xueting Liu [3], Lixin Zhang [3] Ronald J. Quinn [1] and Miaomiao Liu [1,]*

[1] Institute for Biomedicine and Glycomics, Griffith University, Brisbane, QLD, 4111, Australia; r.quinn@griffith.edu.au (R.J.Q.)

[2] Institute for Molecular Bioscience, The University of Queensland, St Lucia, QLD 4072, Australia

[3] State Key Laboratory of Bioreactor Engineering, East China University of Science and Technology, Shanghai 200237, China; liuxueting@ecust.edu.cn (X.L.); lxzhang@ecust.edu.cn (L.Z.)

* Correspondence: jianying.han@uq.edu.au (J.H.); miaomiao.liu@griffith.edu.au (M.L.)

Content

Figure S1. Legend information of the PCA scores plot for Figures 5 and S2 – S5.
Figure S2. PCA Results of 320 Fractions from ES120127.
Figure S3. PCA Results of 320 Fractions from LS120167.
Figure S4. PCA Results of 320 Fractions from MS110105.
Figure S5. PCA Results of 320 Fractions from MS110104.
Figure S6. ^{1}H NMR spectrum of **1** (DMSO-d_6, 800 MHz).
Figure S7. COSY NMR spectrum of **1** (DMSO-d_6, 800 MHz).
Figure S8. HSQC NMR spectrum of **1** (DMSO-d_6, 800 MHz).
Figure S9. HMBC NMR spectrum of **1** (DMSO-d_6, 800 MHz).
Figure S10. HRMS spectrum of **1**.
Figure S11. ^{1}H NMR spectrum of **2** (DMSO-d_6, 800 MHz).
Figure S12. COSY NMR spectrum of **2** (DMSO-d_6, 800 MHz).
Figure S13. HSQC NMR spectrum of **2** (DMSO-d_6, 800 MHz).
Figure S14. HMBC NMR spectrum of **2** (DMSO-d_6, 800 MHz).
Figure S15. HRMS spectrum of **2**.
Figure S16. ^{1}H NMR spectrum of **3** (DMSO-d_6, 800 MHz).
Figure S17. COSY NMR spectrum of **3** (DMSO-d_6, 800 MHz).
Figure S18. HSQC NMR spectrum of **3** (DMSO-d_6, 800 MHz).
Figure S19. HMBC NMR spectrum of **3** (DMSO-d_6, 800 MHz).
Figure S20. HRMS spectrum of **3**.
Figure S21. ^{1}H NMR spectrum of **4** (DMSO-d_6, 600 MHz).
Figure S22. ^{13}C NMR spectrum of **4** (DMSO-d_6, 150 MHz).
Figure S23. COSY NMR spectrum of **4** (DMSO-d_6, 600 MHz).
Figure S24. HSQC NMR spectrum of **4** (DMSO-d_6, 600 MHz).
Figure S25. HMBC NMR spectrum of **4** (DMSO-d_6, 600 MHz).

Figure S26. HRMS spectrum of **4**.
Figure S27. ^{1}H NMR spectrum of **5** (methanol-d_4, 600 MHz).
Figure S28. ^{13}C NMR spectrum of **5** (methanol-d_4, 150 MHz).
Figure S29. COSY NMR spectrum of **5** (methanol-d_4, 600 MHz).
Figure S30. HSQC NMR spectrum of **5** (methanol-d_4, 600 MHz).
Figure S31. HMBC NMR spectrum of **5** (methanol-d_4, 600 MHz).
Figure S32. HRMS spectrum of **5**.
Figure S33. ^{1}H NMR spectrum of **6** (DMSO-d_6, 600 MHz).
Figure S34. COSY NMR spectrum of **6** (DMSO-d_6, 600 MHz).
Figure S35. HSQC NMR spectrum of **6** (DMSO-d_6, 600 MHz).
Figure S36. HRMS spectrum of **6**.
Figure S37. ^{1}H NMR spectrum of **7** (DMSO-d_6, 600 MHz).
Figure S38. COSY NMR spectrum of **7** (DMSO-d_6, 600 MHz).
Figure S39. HSQC NMR spectrum of **7** (DMSO-d_6, 600 MHz).
Figure S40. HRMS spectrum of **7**.
Figure S41. ^{1}H NMR spectrum of **8** (DMSO-d_6, 800 MHz).
Figure S42. ^{13}C NMR spectrum of **8** (DMSO-d_6, 200 MHz).
Figure S43. COSY NMR spectrum of **8** (DMSO-d_6, 800 MHz).
Figure S44. HSQC NMR spectrum of **8** (DMSO-d_6, 800 MHz).
Figure S45. HMBC NMR spectrum of **8** (DMSO-d_6, 800 MHz).
Figure S46. HRMS spectrum of **8**.
Figure S47. ^{1}H NMR spectrum of **9** (DMSO-d_6, 800 MHz).
Figure S48. COSY NMR spectrum of **9** (DMSO-d_6, 800 MHz).
Figure S49. HSQC NMR spectrum of **9** (DMSO-d_6, 800 MHz).
Figure S50. HRMS spectrum of **9**.
Figure S51. ^{1}H NMR spectrum of **10** (DMSO-d_6, 800 MHz).
Figure S52. COSY NMR spectrum of **10** (DMSO-d_6, 800 MHz).
Figure S53. HSQC NMR spectrum of **10** (DMSO-d_6, 800 MHz).
Figure S54. HRMS spectrum of **10**.
Figure S55. ^{1}H NMR spectrum of **11** (DMSO-d_6, 800 MHz).
Figure S56. COSY NMR spectrum of **11** (DMSO-d_6, 800 MHz).
Figure S57. HSQC NMR spectrum of **11** (DMSO-d_6, 800 MHz).
Figure S58. HRMS spectrum of **11**.

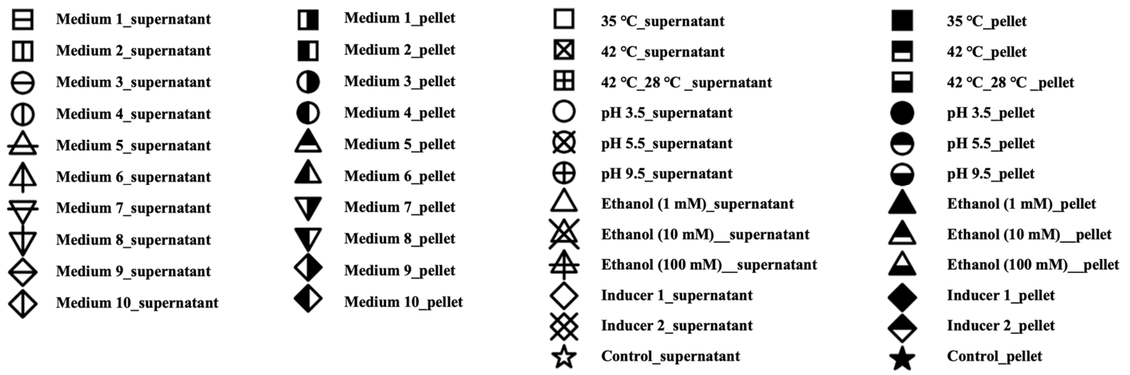

Figure S1: Legend information of the PCA scores plot for Figures 5 and S2-S5.

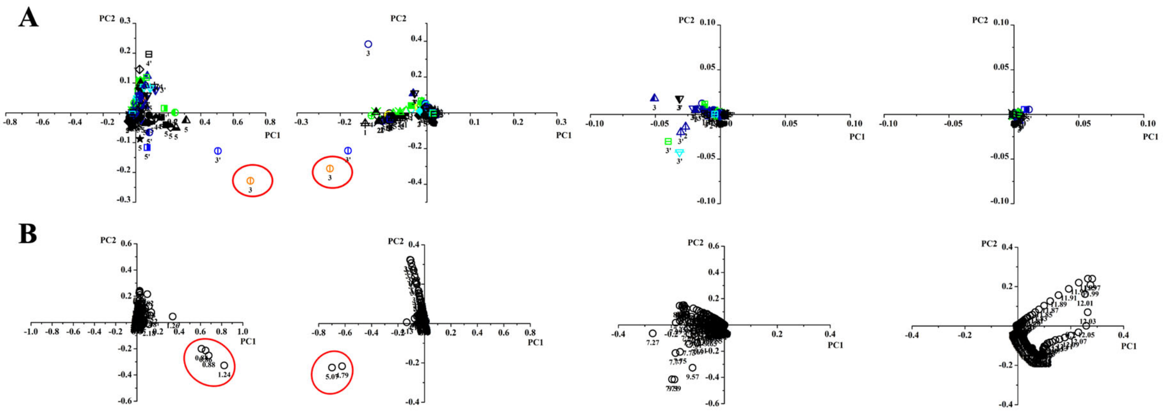

Figure S2: PCA Results of 320 Fractions from ES120127. Anti-BCG activity is indicated by color coding according to the legend in Figure 2 (blue for lowest activity, red for highest activity). A. From left to right, score plots of NMR chemical shifts in the regions 0–2.4 ppm, 3.5–6 ppm, 6–10 ppm, and 10–15 ppm. Each type of symbol represents fractions derived from an OSMAC condition (see symbol details in the supporting information). Fraction numbers are indicated below each symbol, with the supernatant fractions labeled as 1 to 5 and the cell pellet fractions labeled as 1' to 5'. The fraction highlighted with a red circle is the selected fraction: fraction 3, derived from the supernatant extract cultured in medium 4. B. Loading plots of NMR chemical shifts for the regions 0–2.4 ppm, 3.5–6 ppm, 6–10 ppm, and 10–15 ppm. Bucket values in the loading plots are shown as numbers below each circle (in ppm).

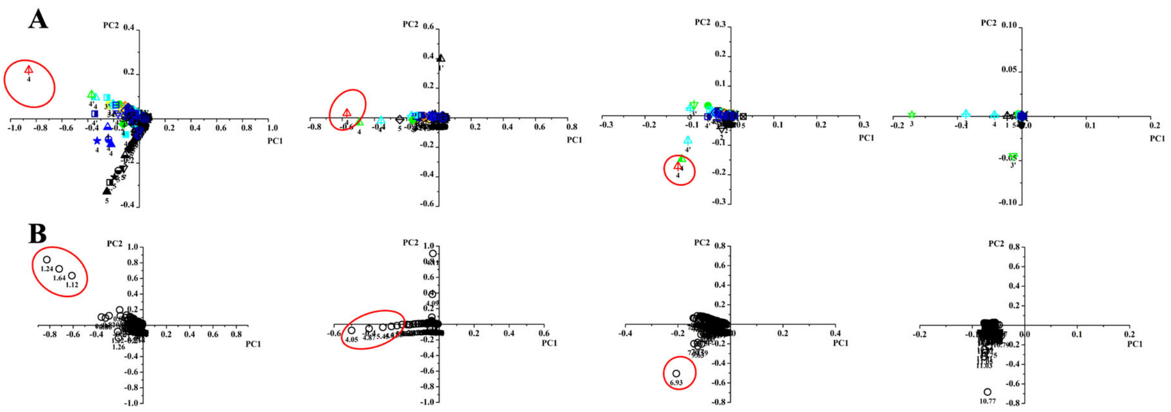

Figure S3. PCA Results of 320 Fractions from LS120167. Anti-BCG activity is indicated by color coding according to the legend in Figure 2 (blue for lowest activity, red for highest activity). A. From left to right, score plots of NMR chemical shifts in the regions 0–2.4 ppm, 3.5–6 ppm, 6–10 ppm, and 10–15 ppm. Each type of symbol represents fractions derived from an OSMAC condition (see symbol details in the supporting information). Fraction numbers are indicated below each symbol, with the supernatant fractions labeled as 1 to 5 and the cell pellet fractions labeled as 1' to 5'. The fraction highlighted with a red circle is the selected fraction: fraction 4 from the supernatant extract cultured in medium 6. B. Loading plots of NMR chemical shifts for the regions 0–2.4 ppm, 3.5–6 ppm, 6–10 ppm, and 10–15 ppm. Bucket values in the loading plots are shown as numbers below each circle (in ppm).

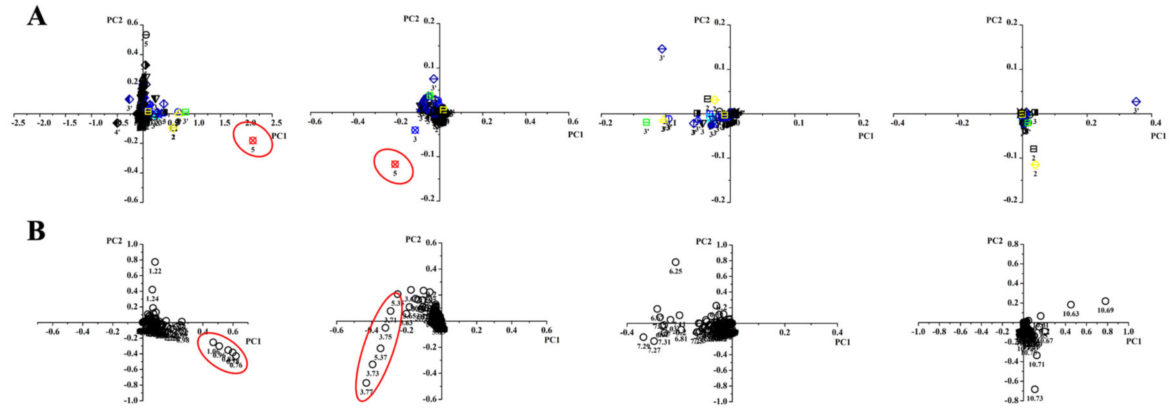

Figure S4. PCA Results of 320 Fractions from MS110105. Anti-BCG activity is indicated by color coding according to the legend in Figure 2 (blue for lowest activity, red for highest activity). A. From left to right, score plots of NMR chemical shifts in the regions 0–2.4 ppm, 3.5–6 ppm, 6–10 ppm, and 10–15 ppm. Each type of symbol represents fractions derived from an OSMAC condition (see symbol details in the supporting information). Fraction numbers are indicated below each symbol, with the supernatant fractions labeled as 1 to 5 and the cell pellet fractions labeled as 1' to 5'. The fraction highlighted with a red circle is the selected fraction: fraction 5 from the supernatant extract cultured at pH 5.5. B. Loading plots of NMR chemical shifts for the regions 0–2.4 ppm, 3.5–6 ppm, 6–10 ppm, and 10–15 ppm. Bucket values in the loading plots are shown as numbers below each circle (in ppm).

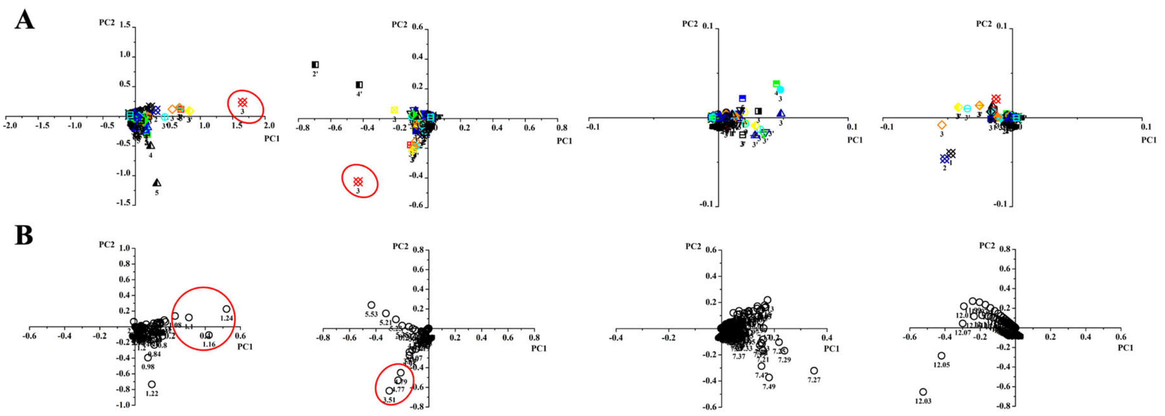

Figure S5. PCA Results of 320 Fractions from MS110104. Anti-BCG activity is indicated by color coding according to the legend in Figure 2 (blue for lowest activity, red for highest activity). A. From left to right, score plots of NMR chemical shifts in the regions 0–2.4 ppm, 3.5–6 ppm, 6–10 ppm, and 10–15 ppm. Each type of symbol represents fractions derived from an OSMAC condition (see symbol details in the supporting information). Fraction numbers are indicated below each symbol, with the supernatant fractions labeled as 1 to 5 and the cell pellet fractions labeled as 1' to 5'. The fraction highlighted with a red circle is the selected fraction: fraction 3 from the supernatant extract cultured with the inducer molecule N-carbobenzoxy-L-homoserine lactone. B. Loading plots of NMR chemical shifts for the regions 0–2.4 ppm, 3.5–6 ppm, 6–10 ppm, and 10–15 ppm. Bucket values in the loading plots are shown as numbers below each circle (in ppm).

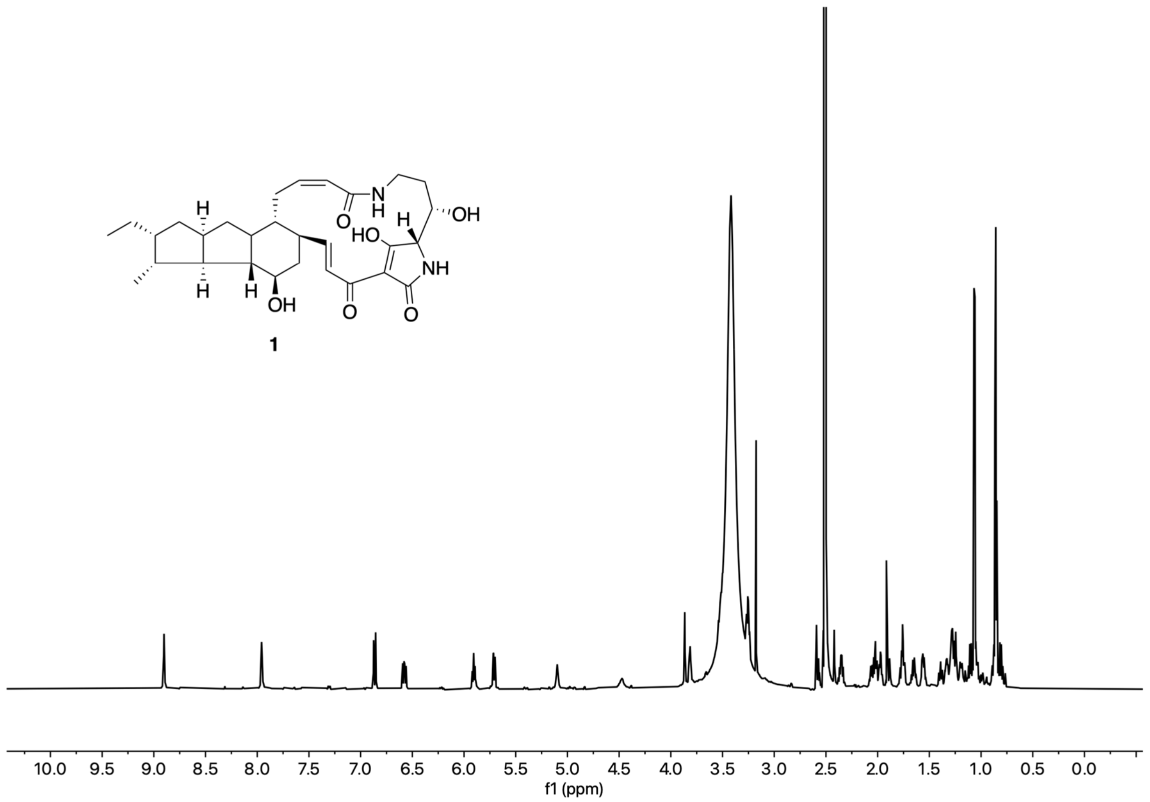

Figure S6. ^{1}H NMR spectrum of **1** (DMSO-d_6, 800 MHz).

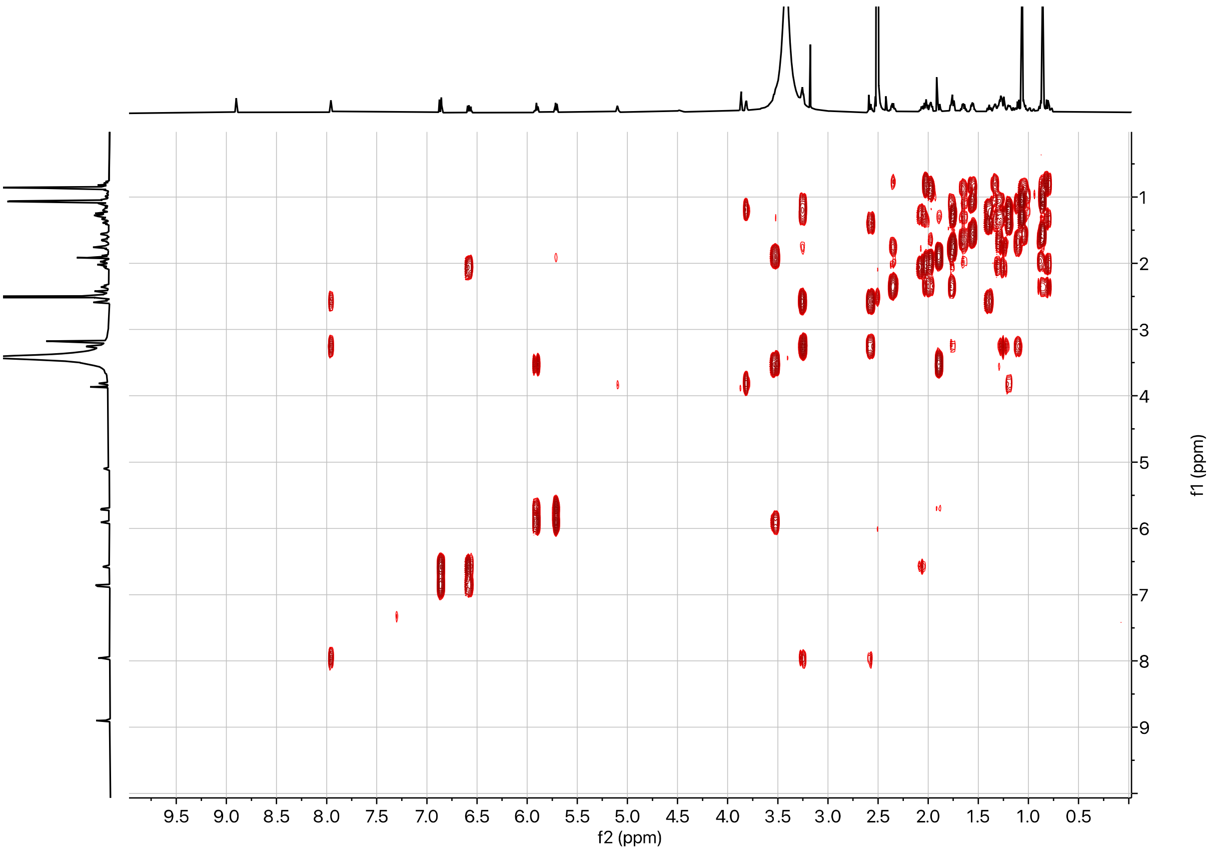

Figure S7. COSY NMR spectrum of **1** (DMSO-d_6, 800 MHz).

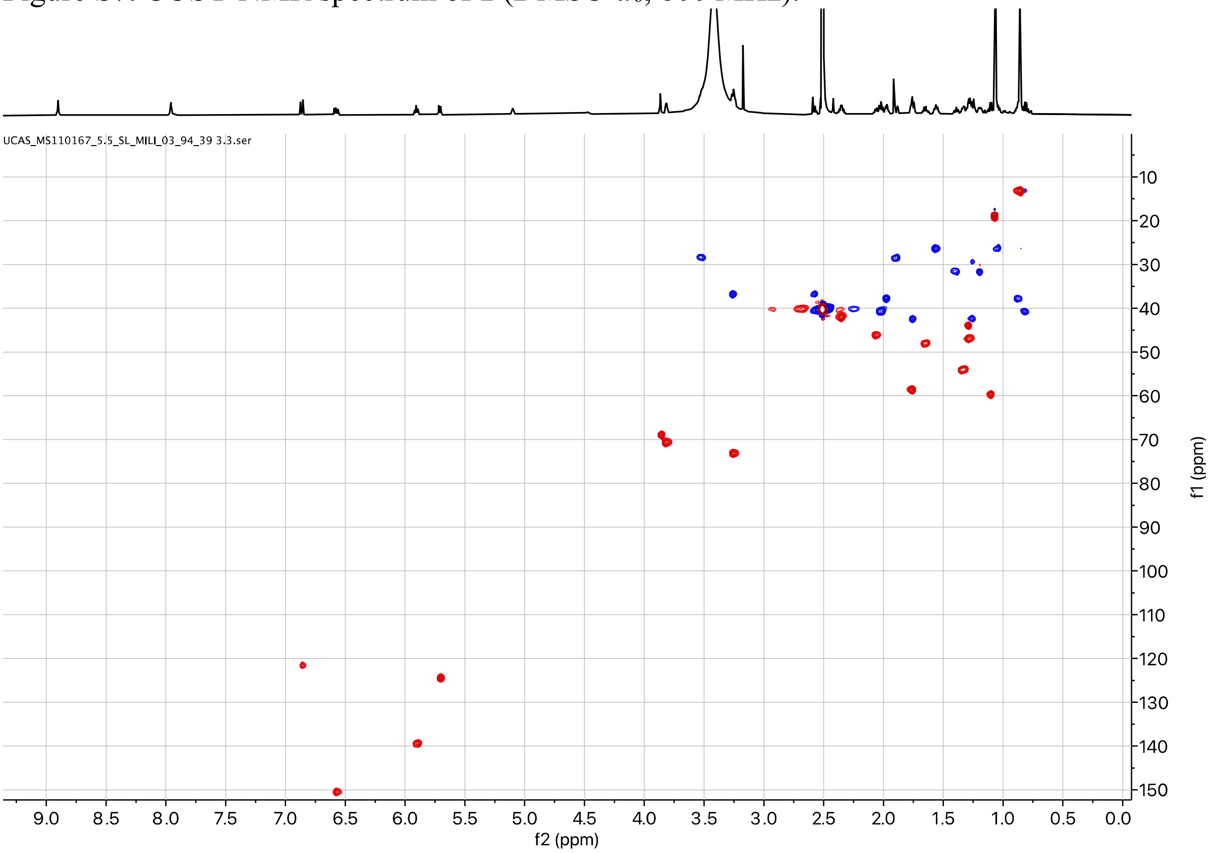

Figure S8. HSQC NMR spectrum of **1** (DMSO-d_6, 800 MHz).

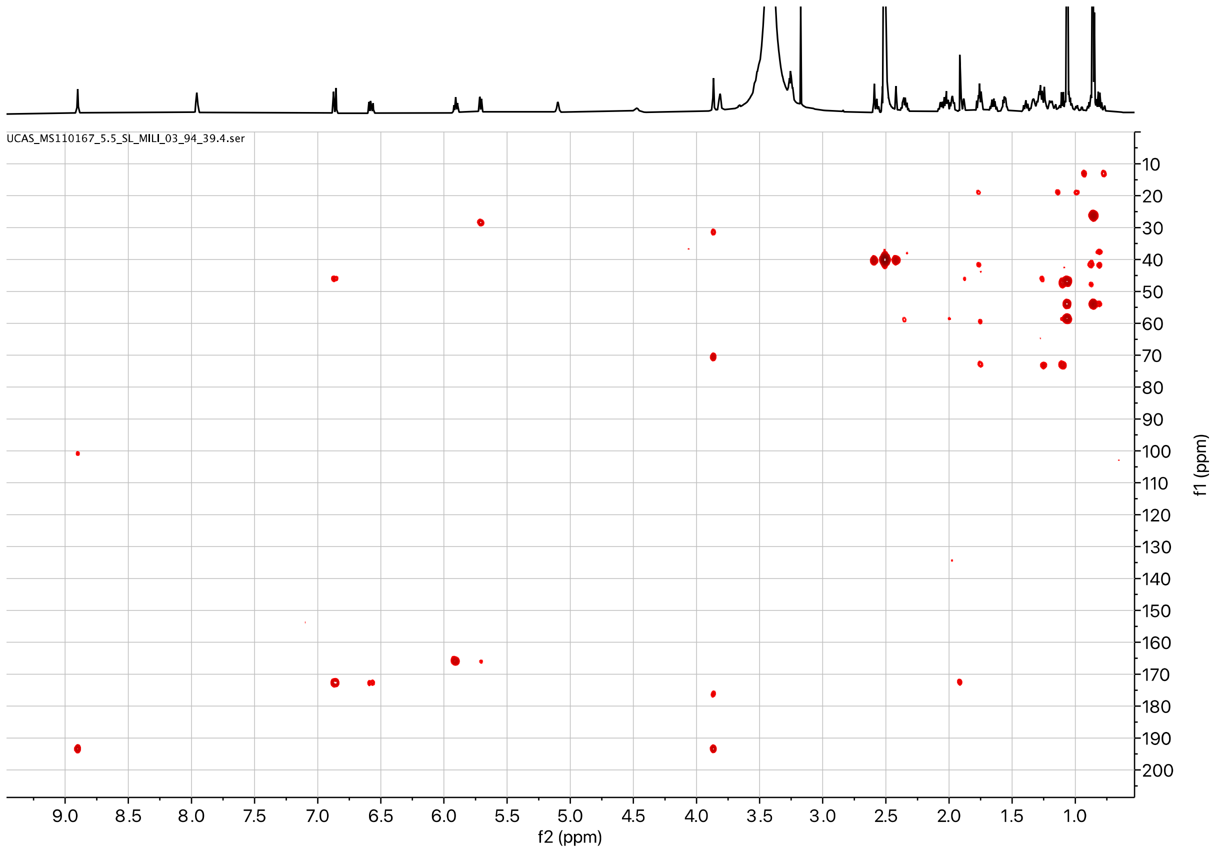

Figure S9. HMBC NMR spectrum of **1** (DMSO-d_6, 800 MHz).

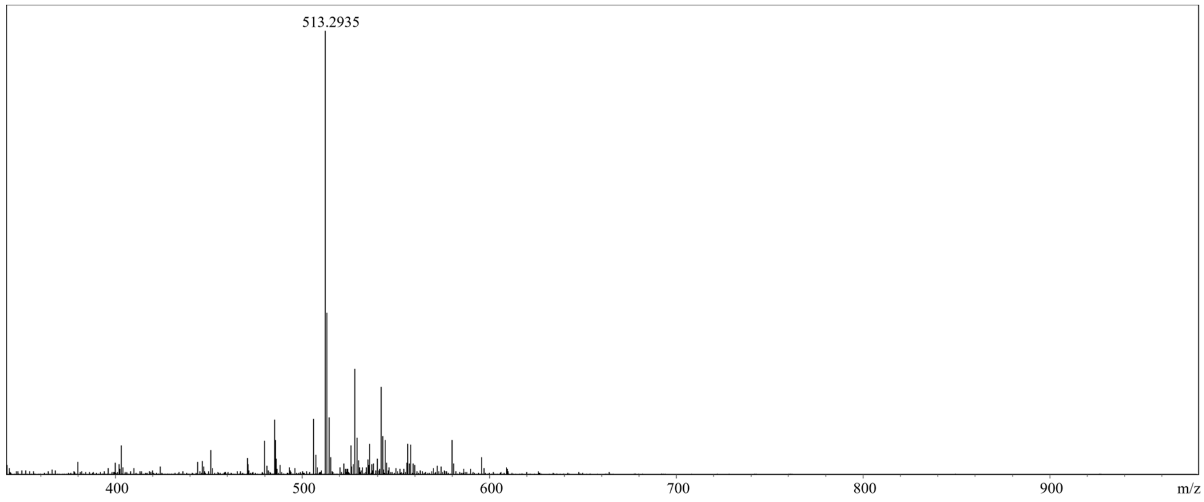

Figure S10. HRMS spectrum of **1**.

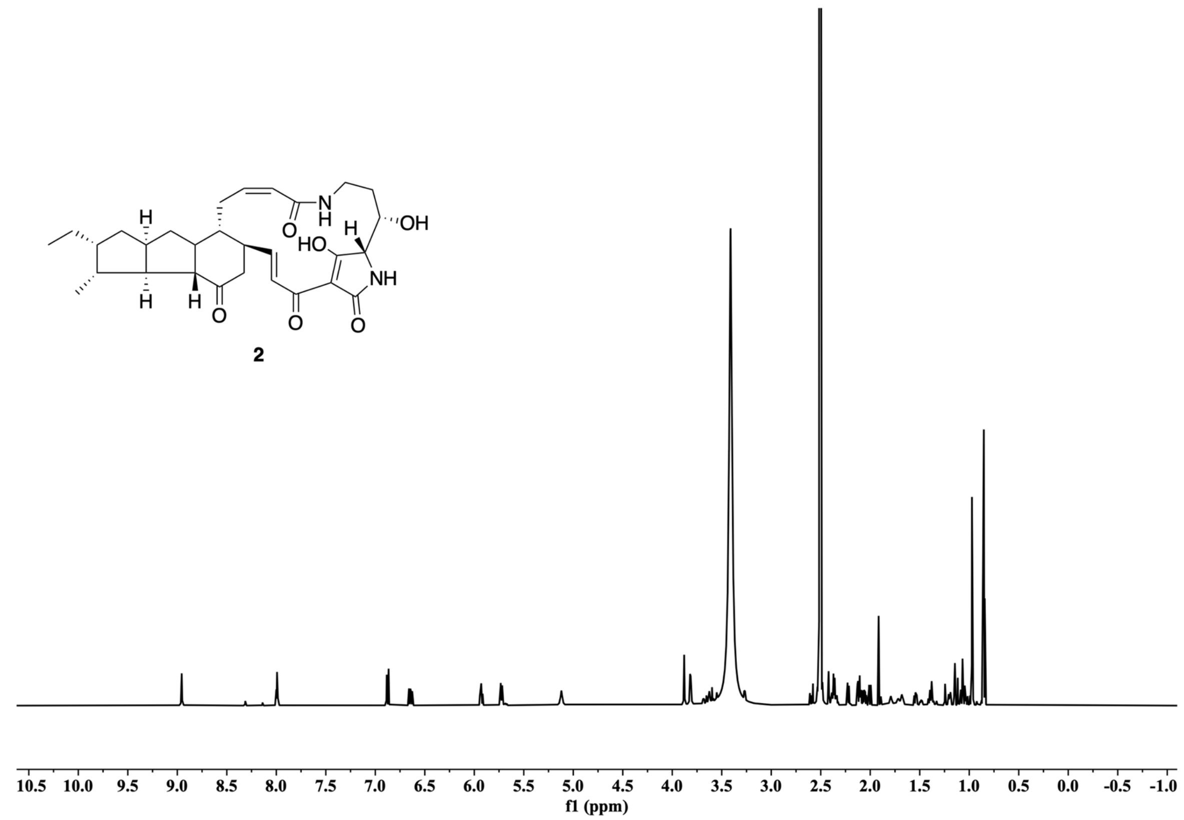

Figure S11. ^{1}H NMR spectrum of **2** (DMSO-*d*6, 800 MHz).

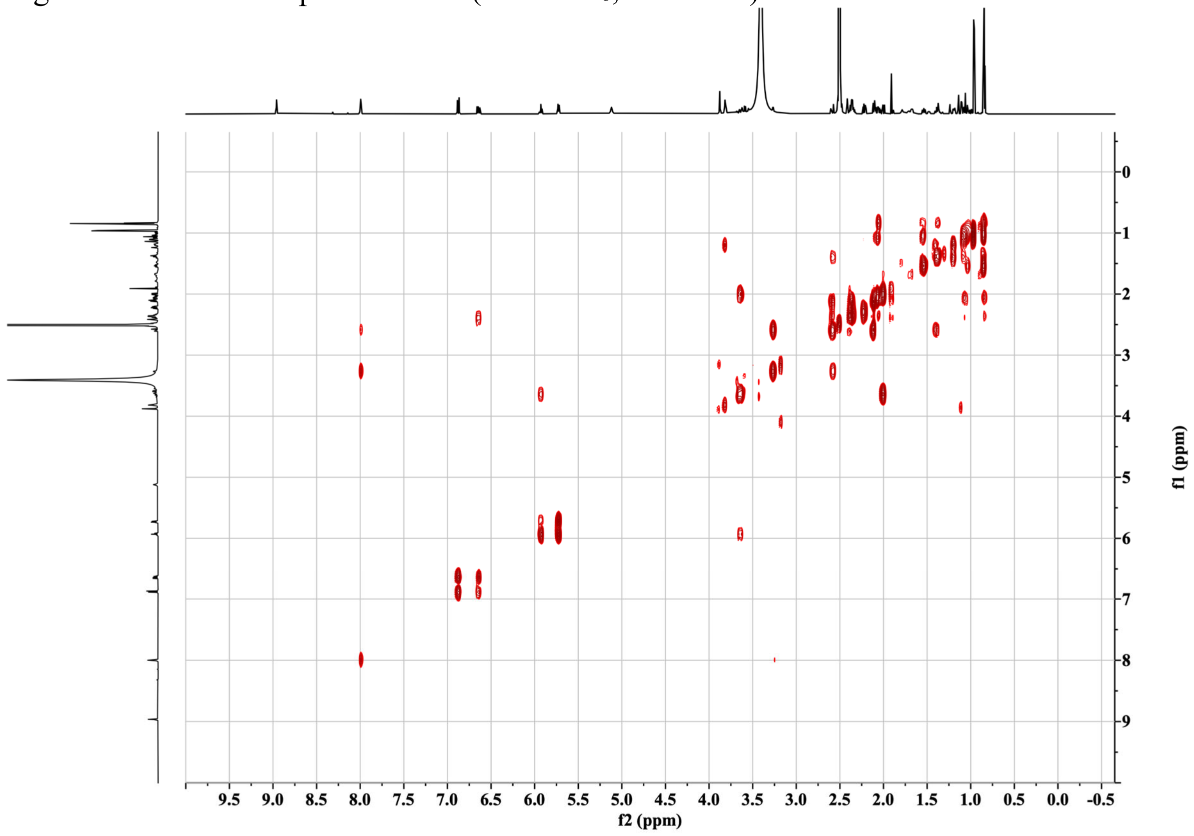

Figure S12. COSY NMR spectrum of **2** (DMSO-*d*6, 800 MHz).

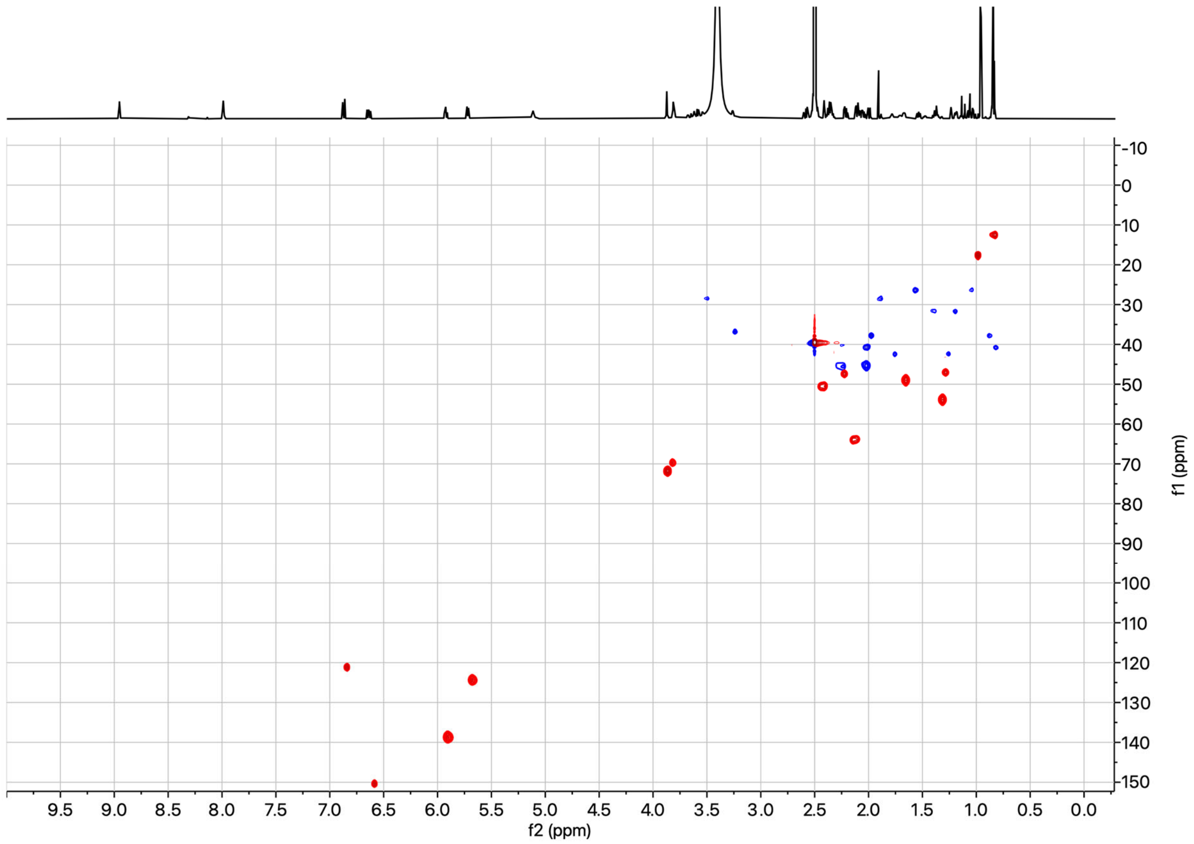

Figure S13. HSQC NMR spectrum of **2** (DMSO-d_6, 800 MHz).

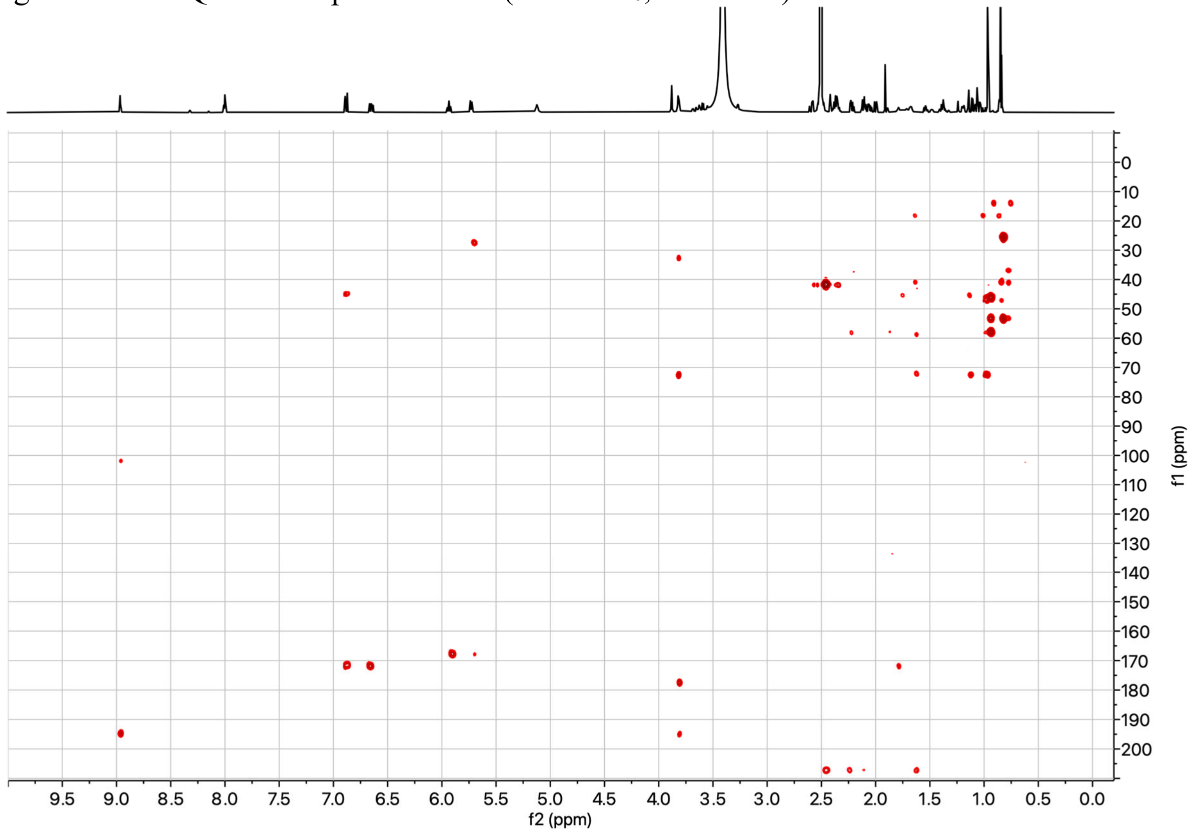

Figure S14. HMBC NMR spectrum of **2** (DMSO-d_6, 800 MHz).

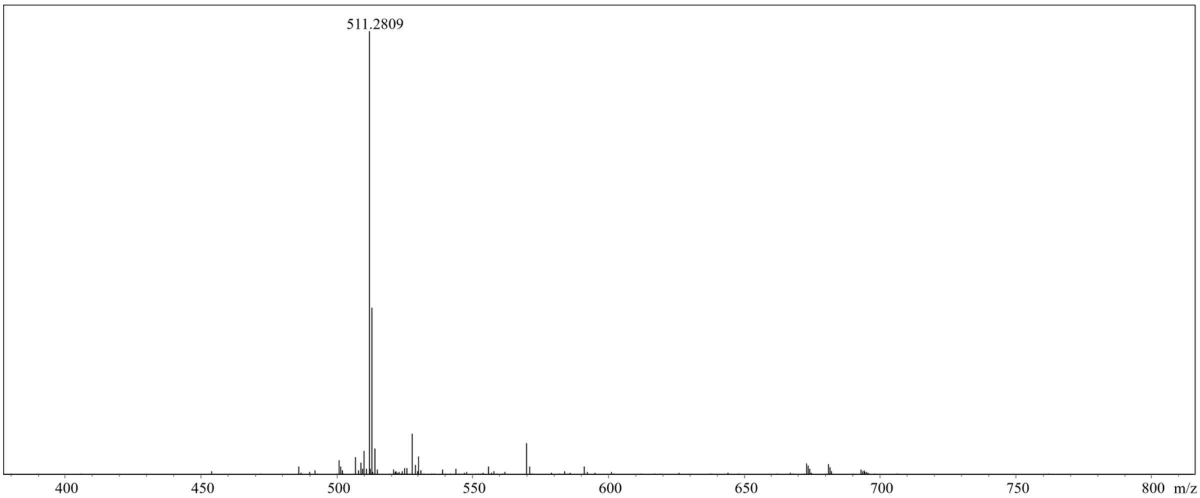

Figure S15. HRMS spectrum of **2**.

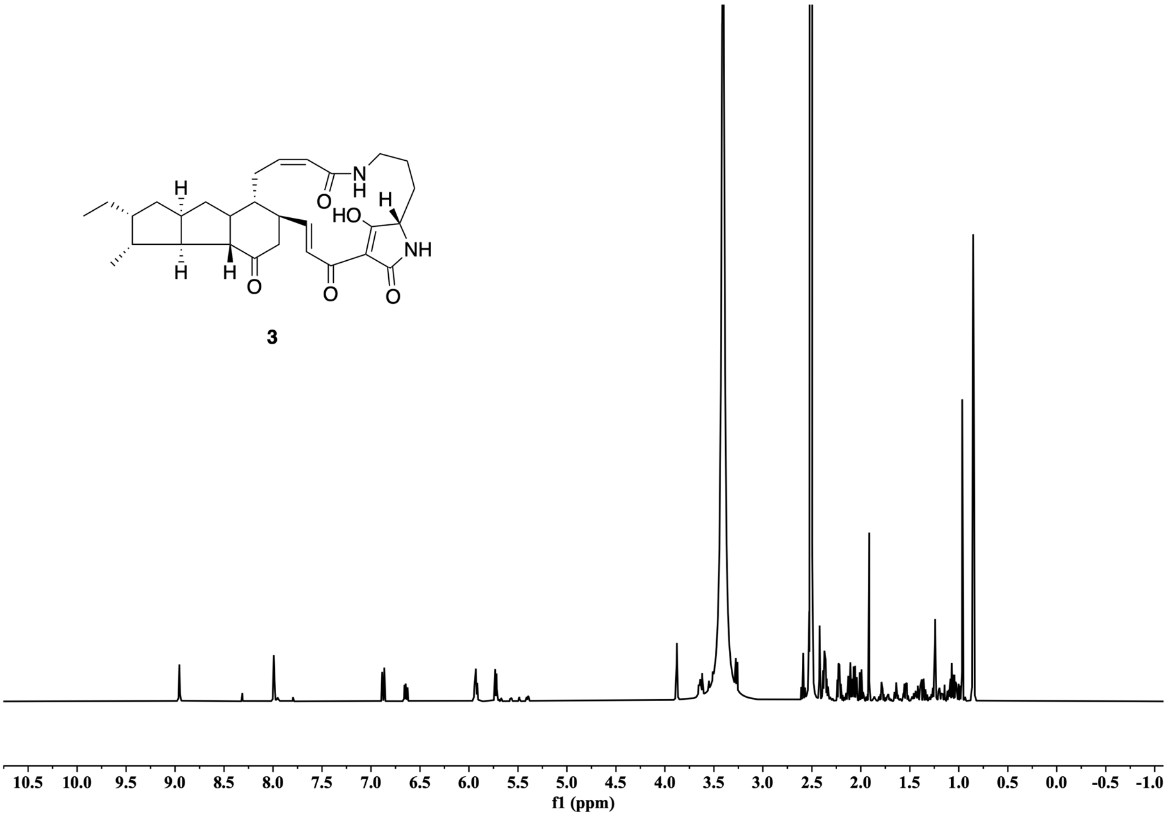

Figure S16. ^{1}H NMR spectrum of **3** (DMSO-d_6, 800 MHz).

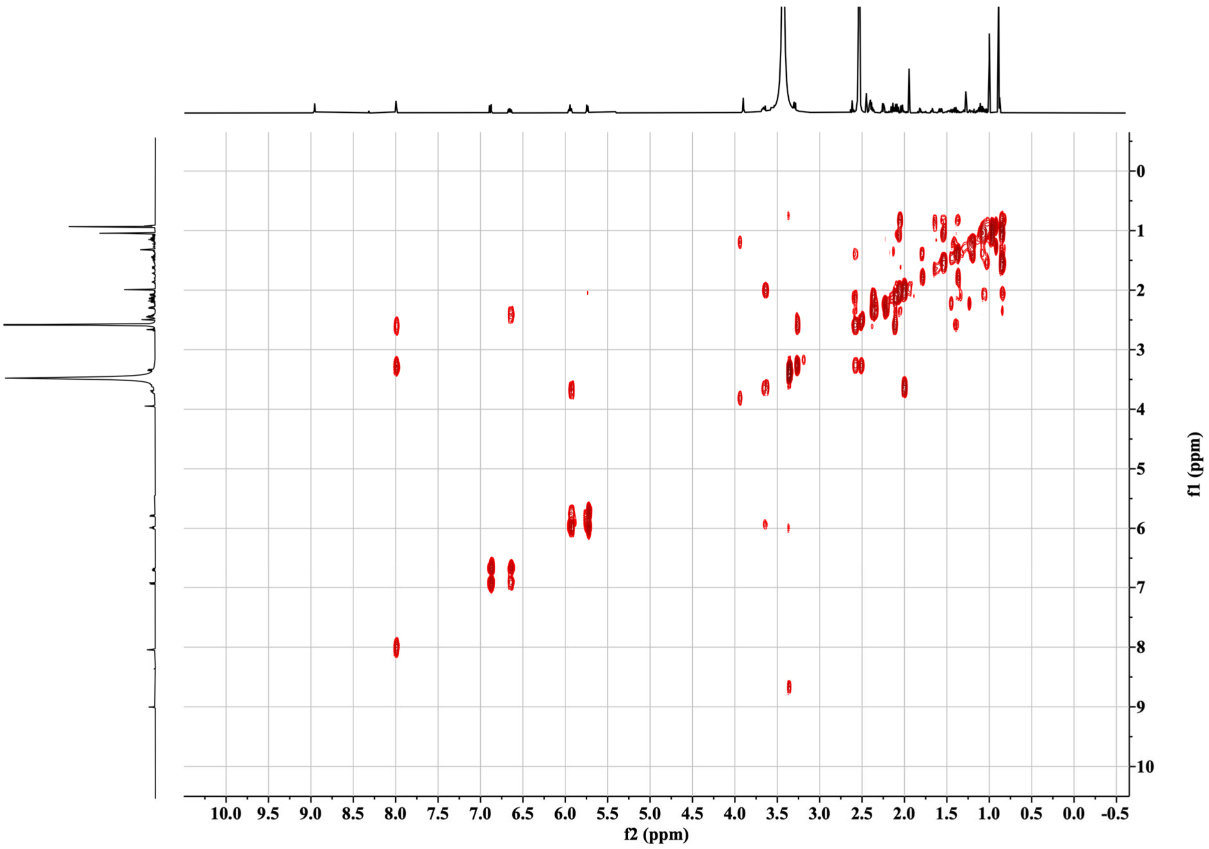

Figure S17. COSY NMR spectrum of **3** (DMSO-d_6, 800 MHz).

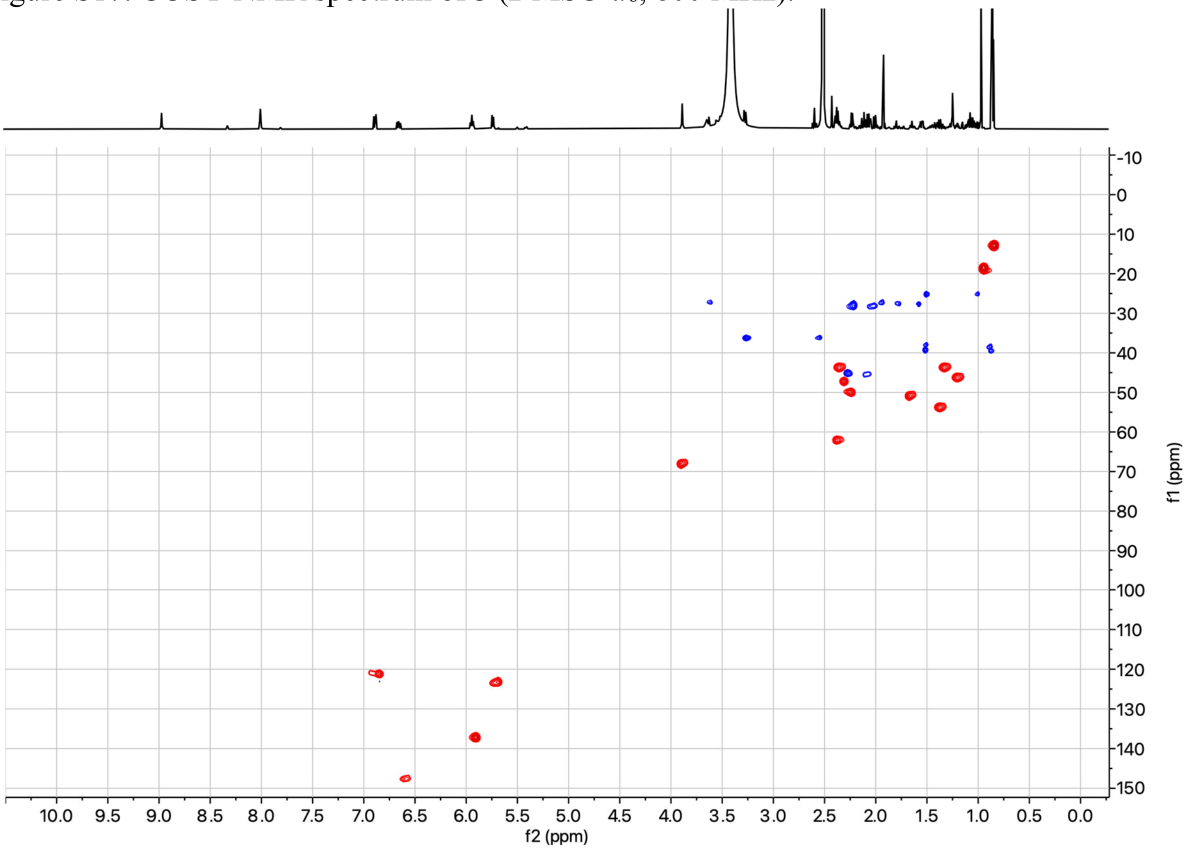

Figure S18. HSQC NMR spectrum of **3** (DMSO-d_6, 800 MHz).

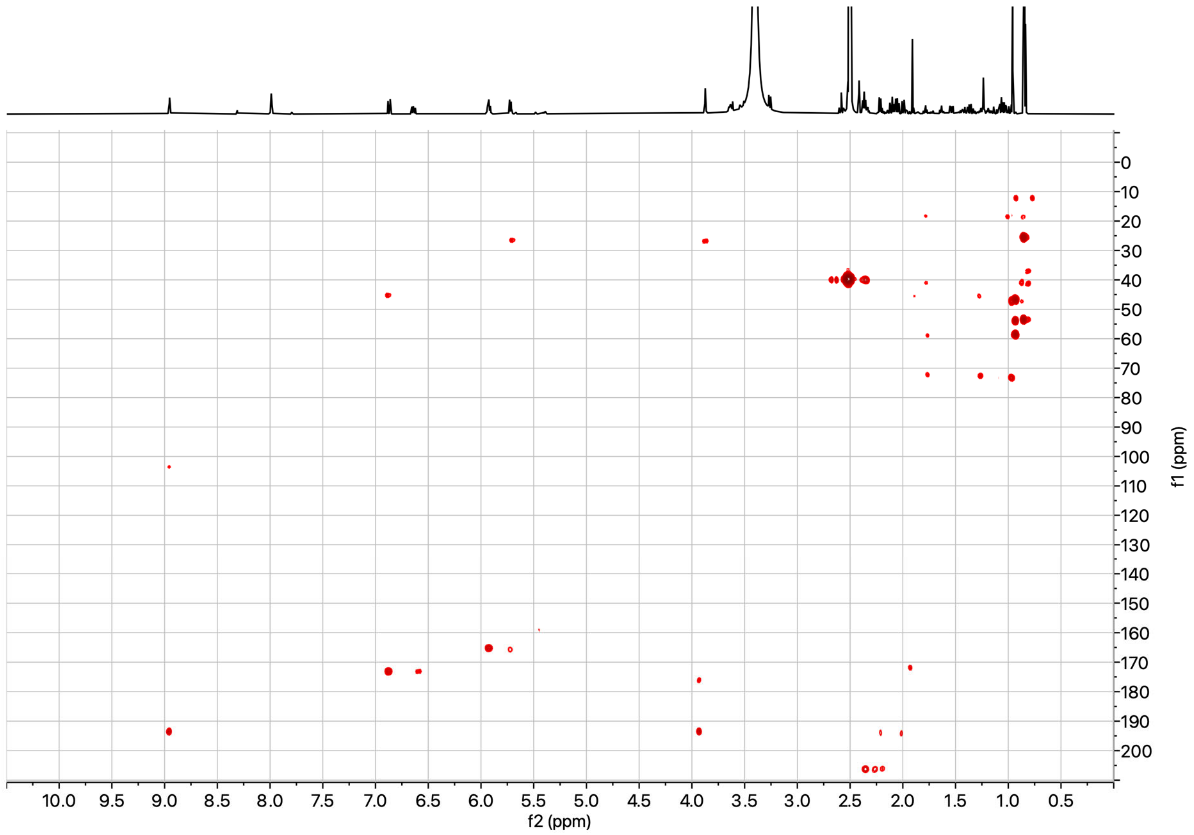

Figure S19. HMBC NMR spectrum of **3** (DMSO-d_6, 800 MHz).

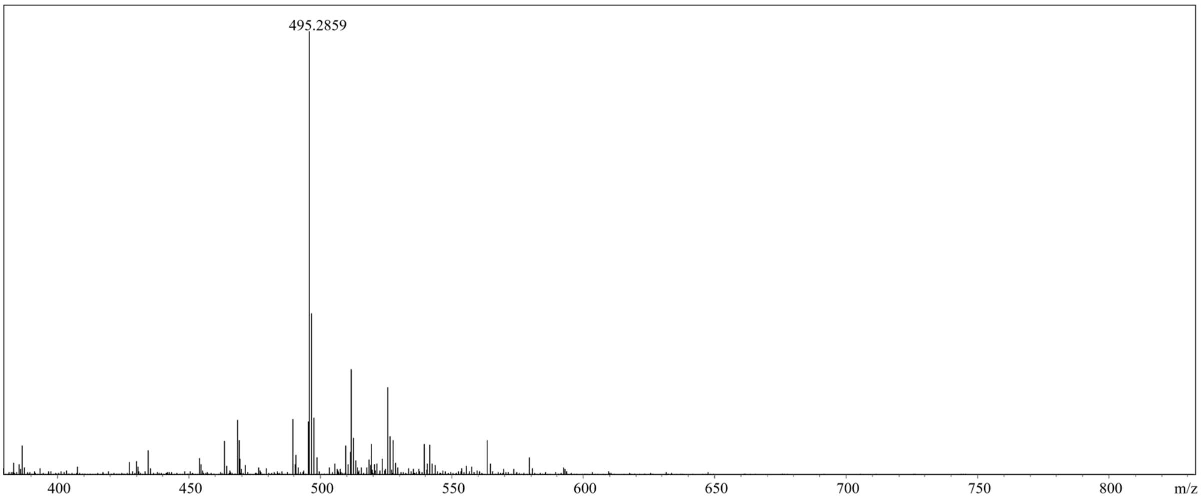

Figure S20. HRMS spectrum of **3**.

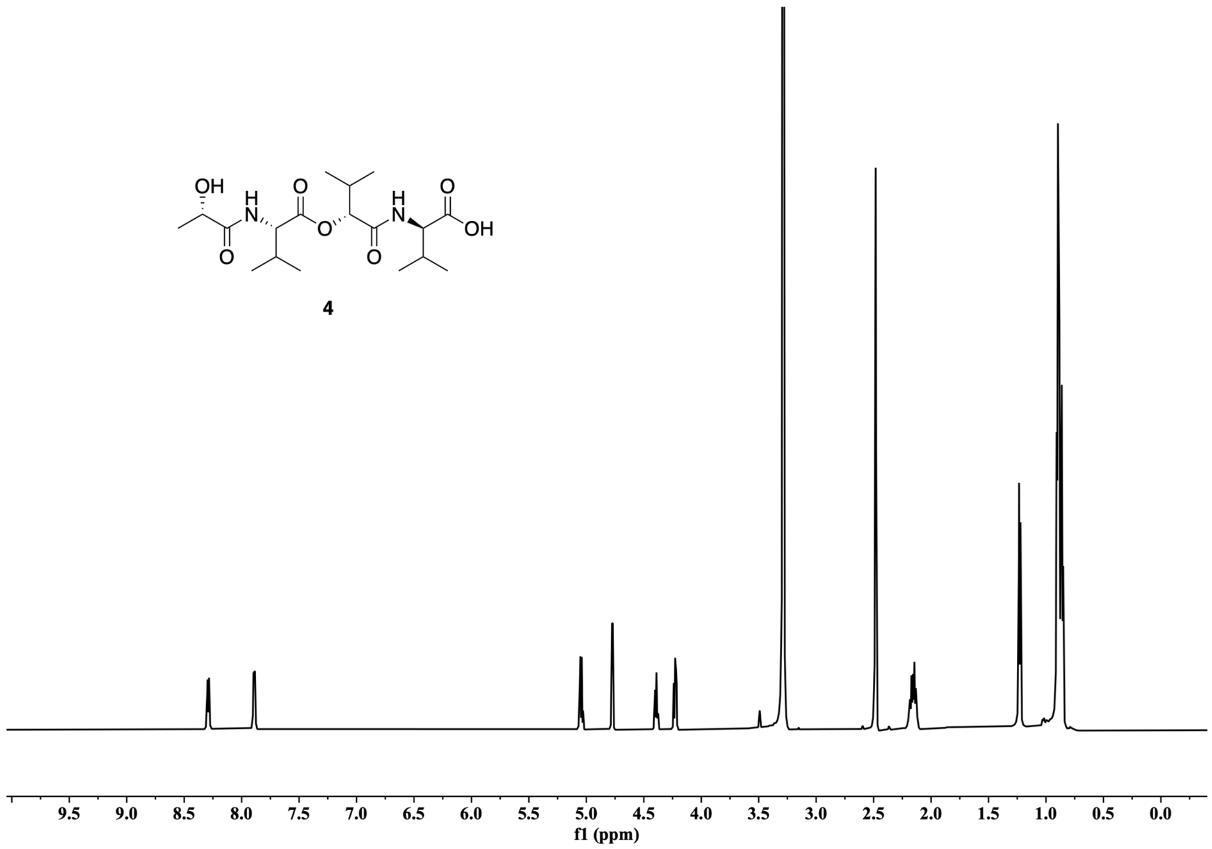

Figure S21. ^{1}H NMR spectrum of **4** (DMSO-d_6, 600 MHz).

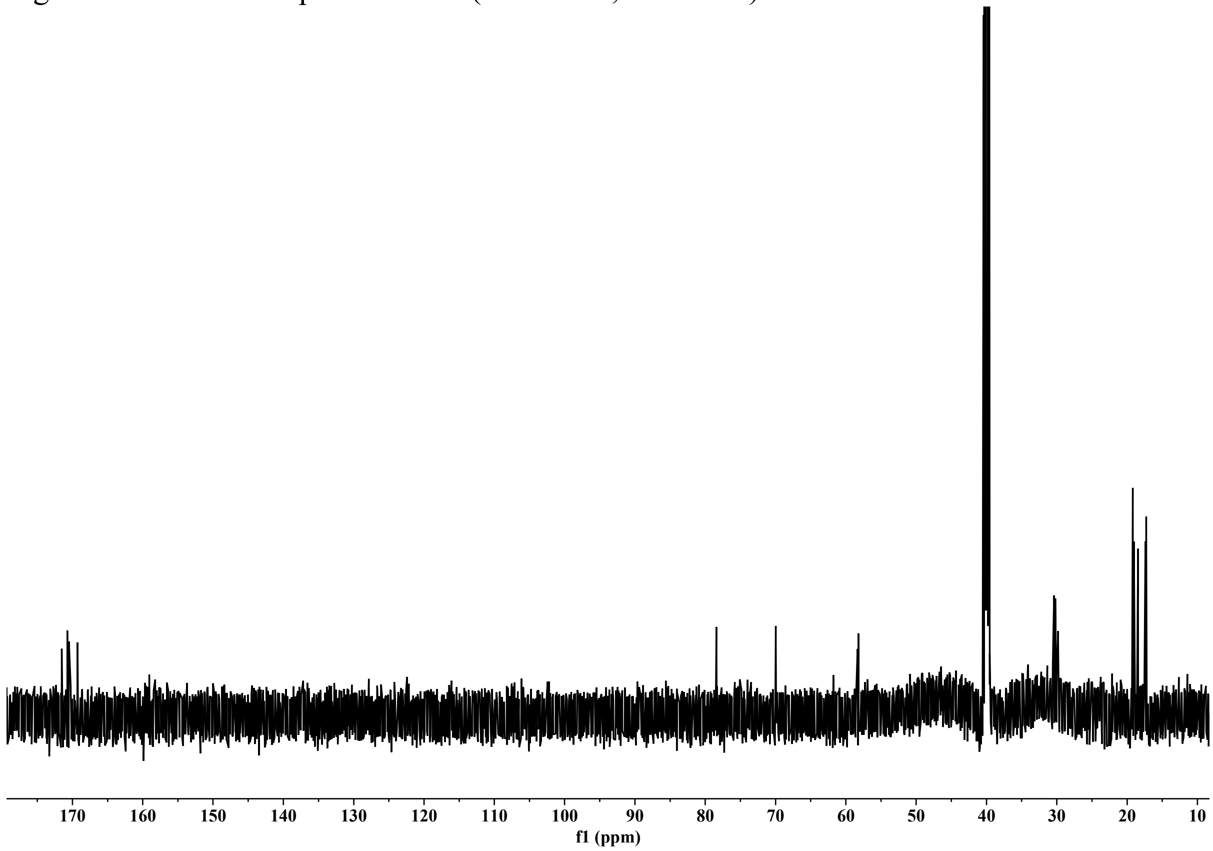

Figure S22. ^{13}C NMR spectrum of **4** (DMSO-d_6, 150 MHz).

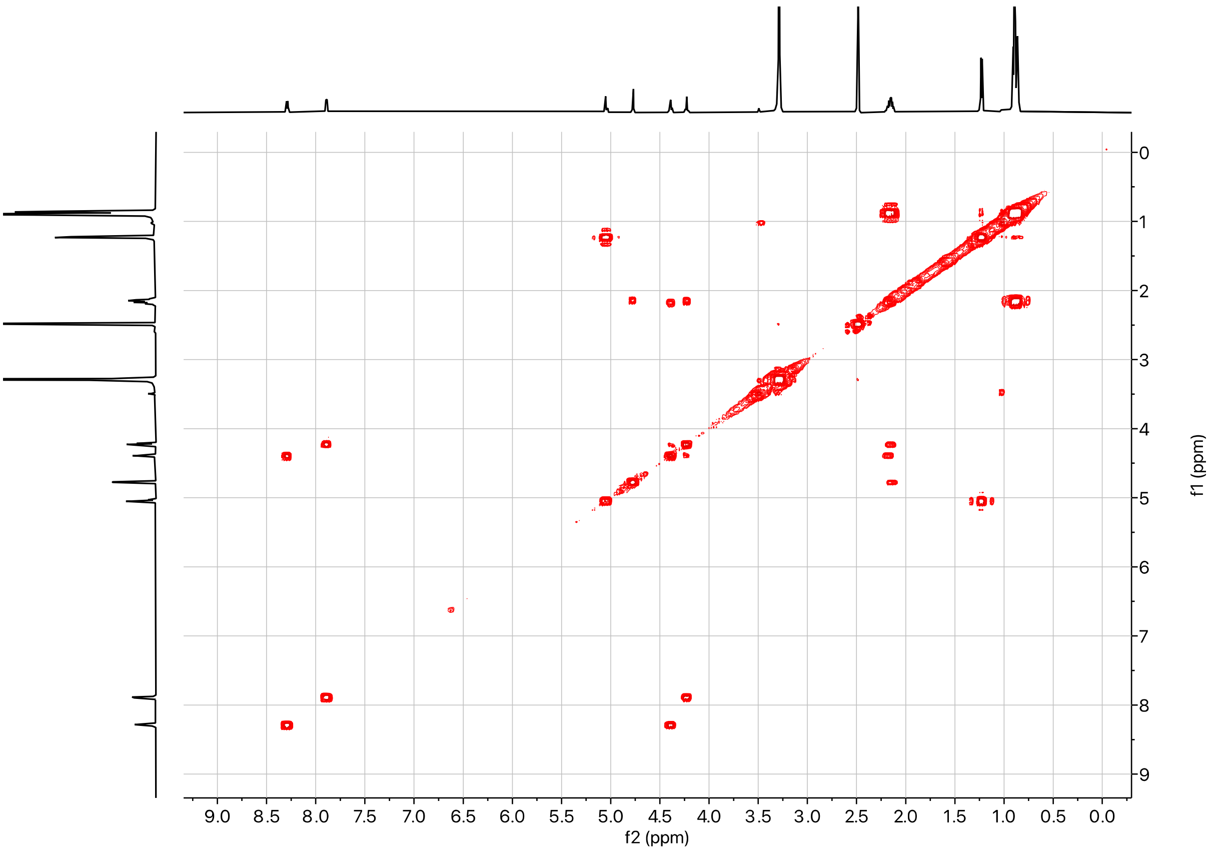

Figure S23. COSY NMR spectrum of **4** (DMSO-d_6, 600 MHz).

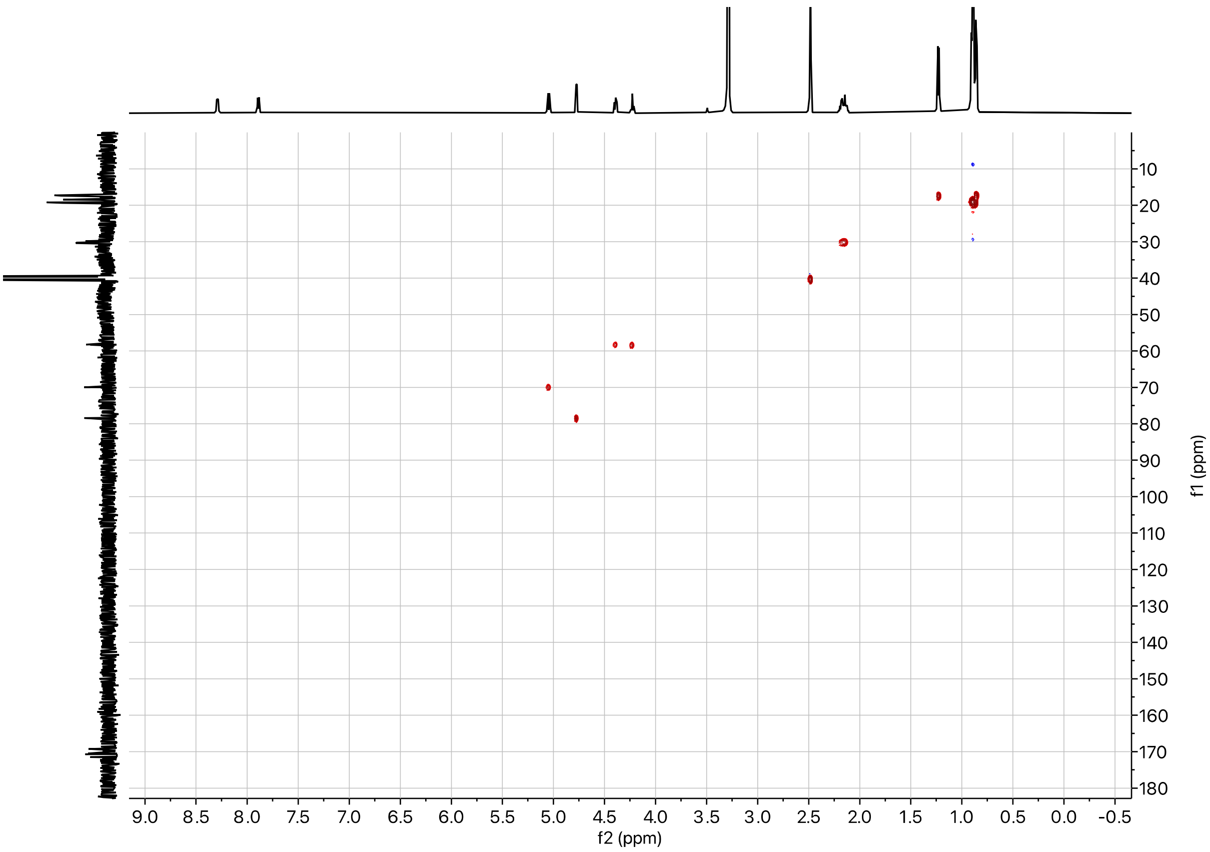

Figure S24. HSQC NMR spectrum of **4** (DMSO-d_6, 600 MHz).

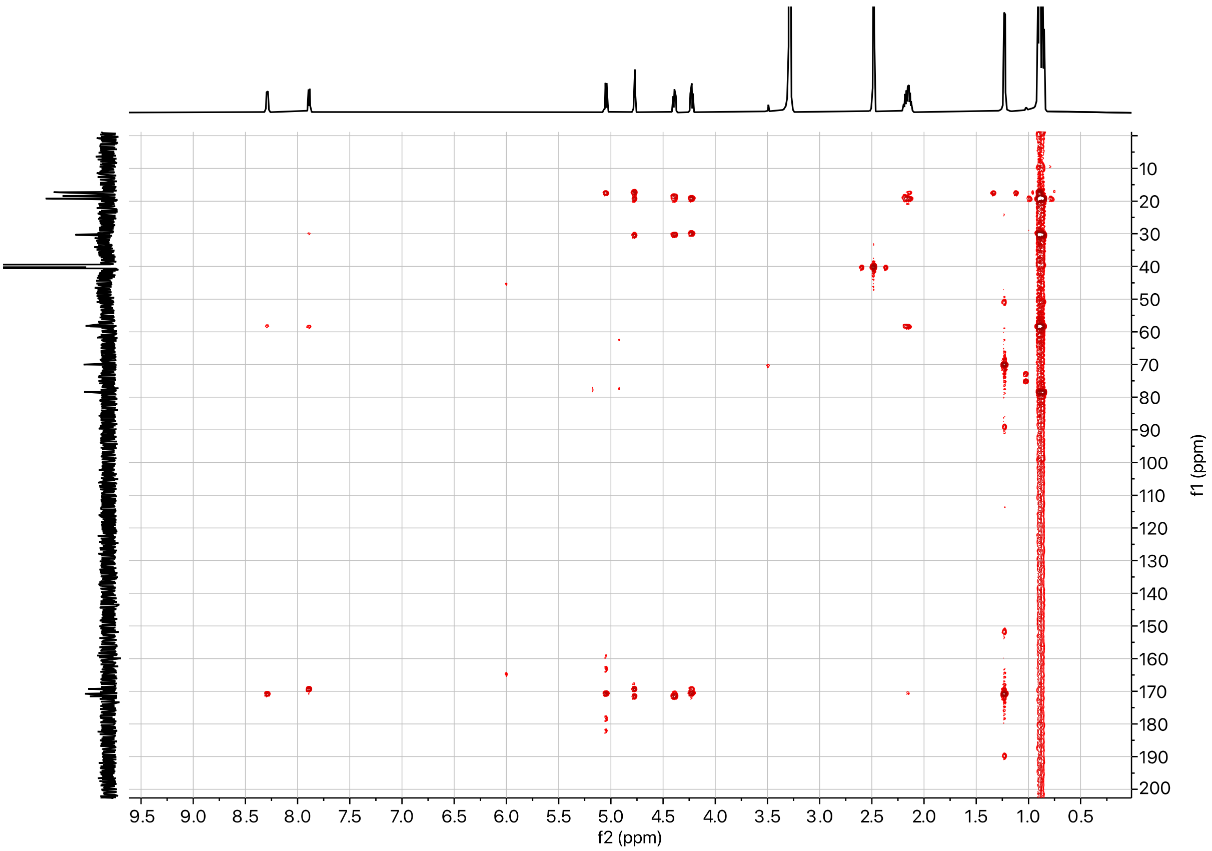

Figure S25. HMBC NMR spectrum of **4** (DMSO-d_6, 600 MHz).

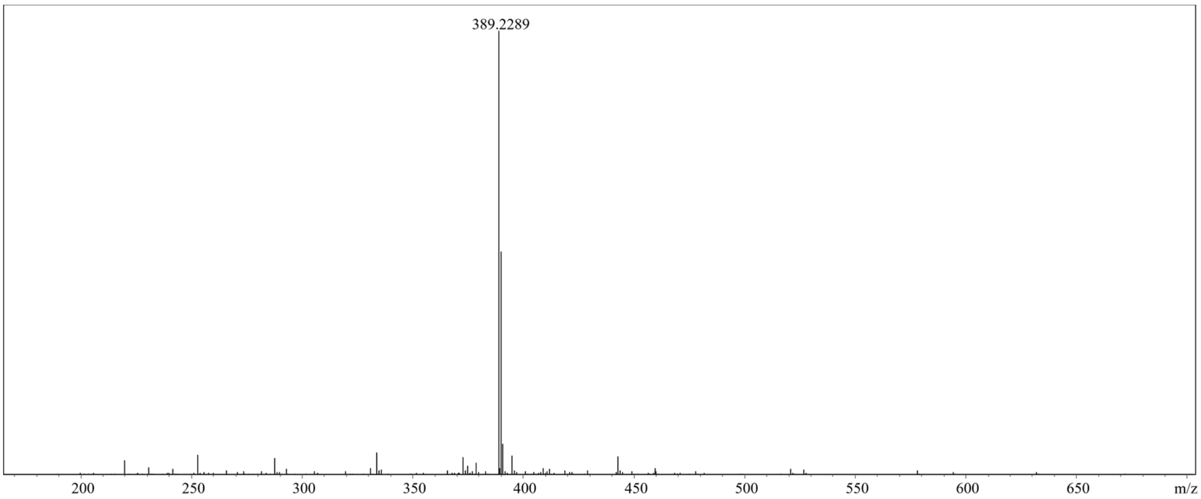

Figure S26. HRMS spectrum of **4**.

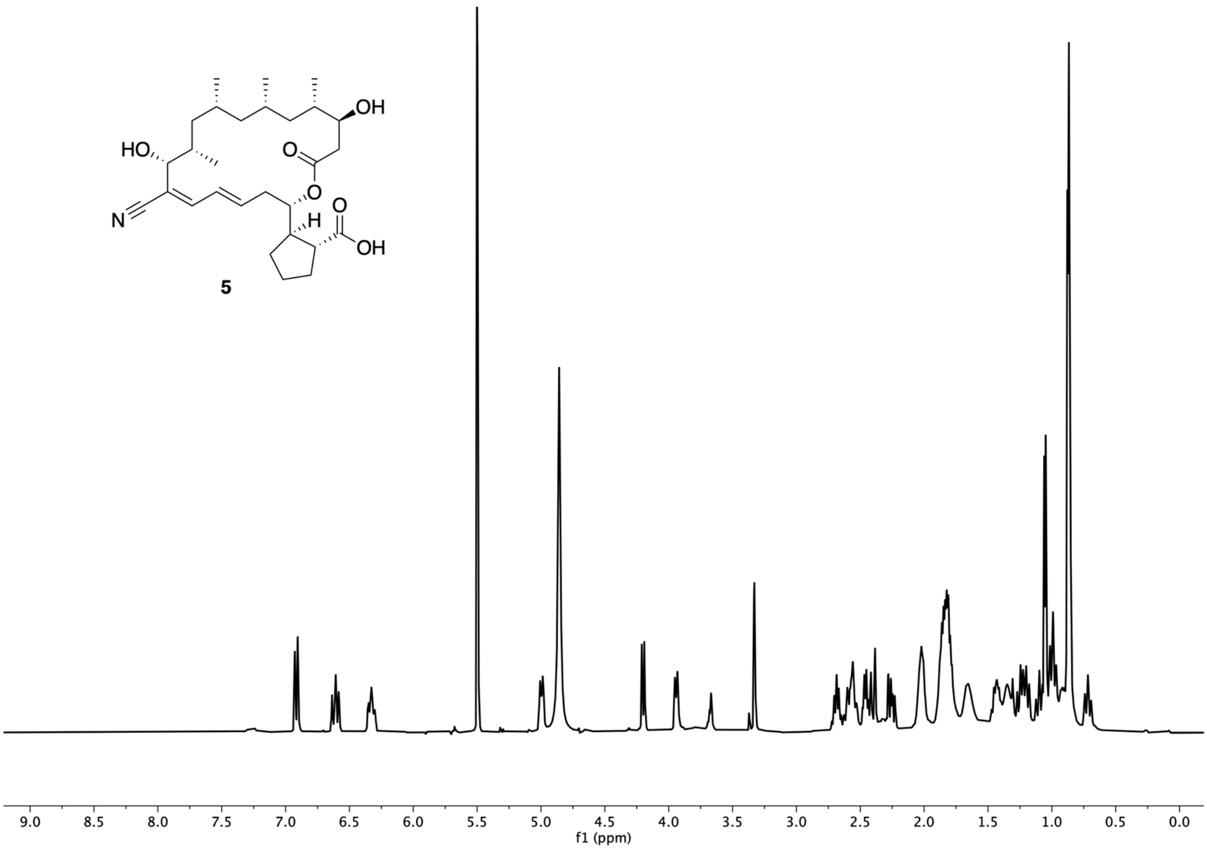

Figure S27. ^{1}H NMR spectrum of **5** (methanol-*d*4, 600 MHz).

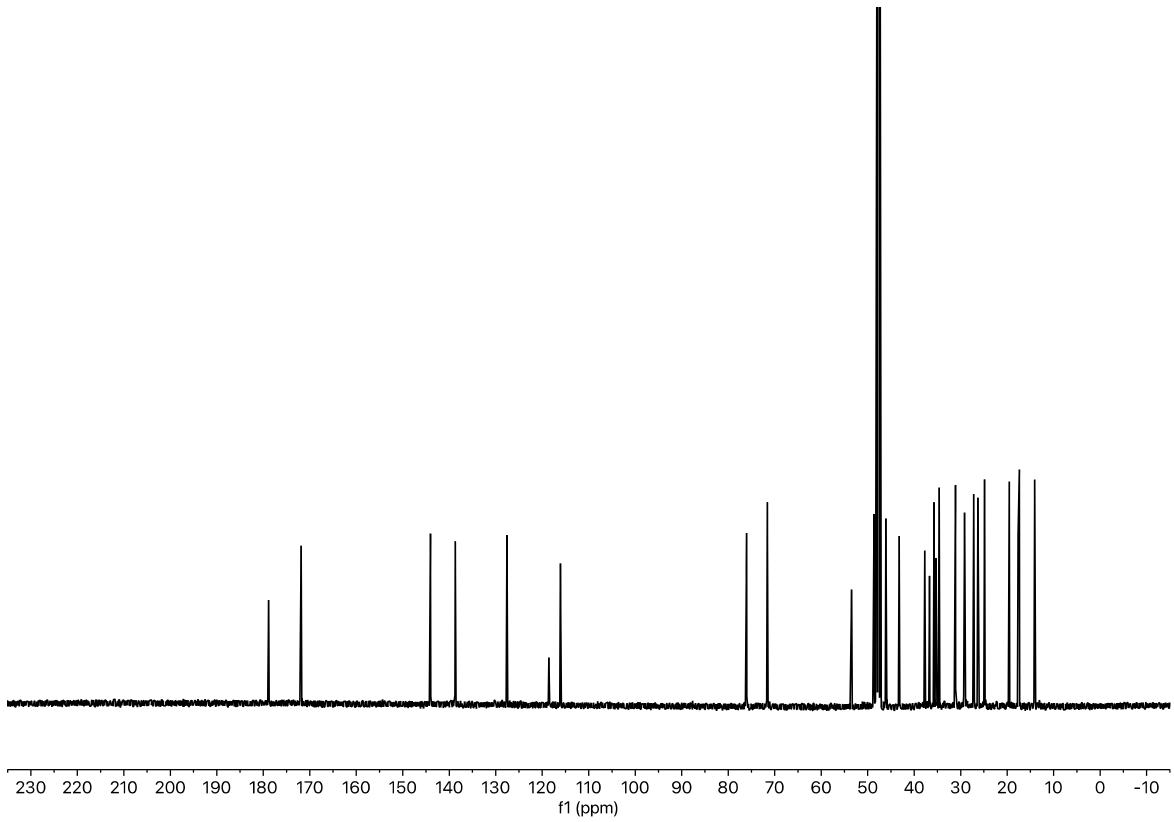

Figure S28. ^{13}C NMR spectrum of **5** (methanol-*d*4, 150 MHz).

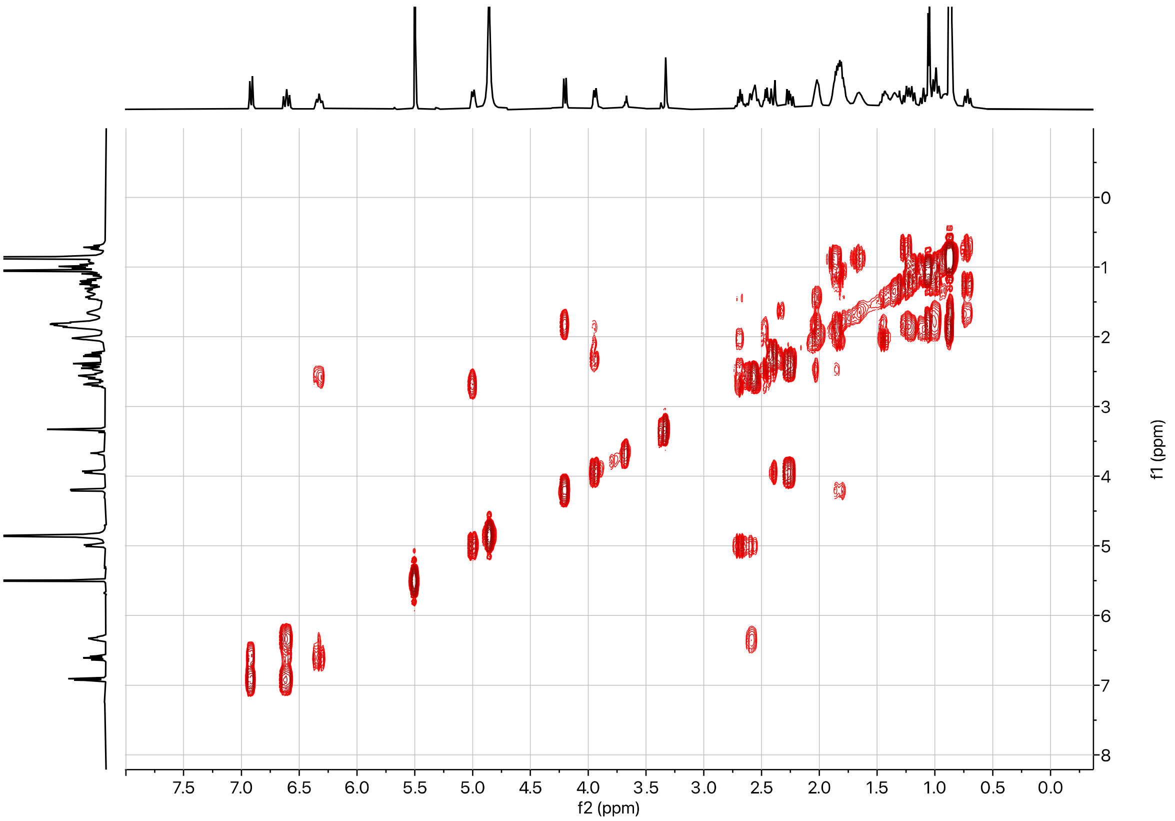

Figure S29. COSY NMR spectrum of **5** (methanol-*d*4, 600 MHz).

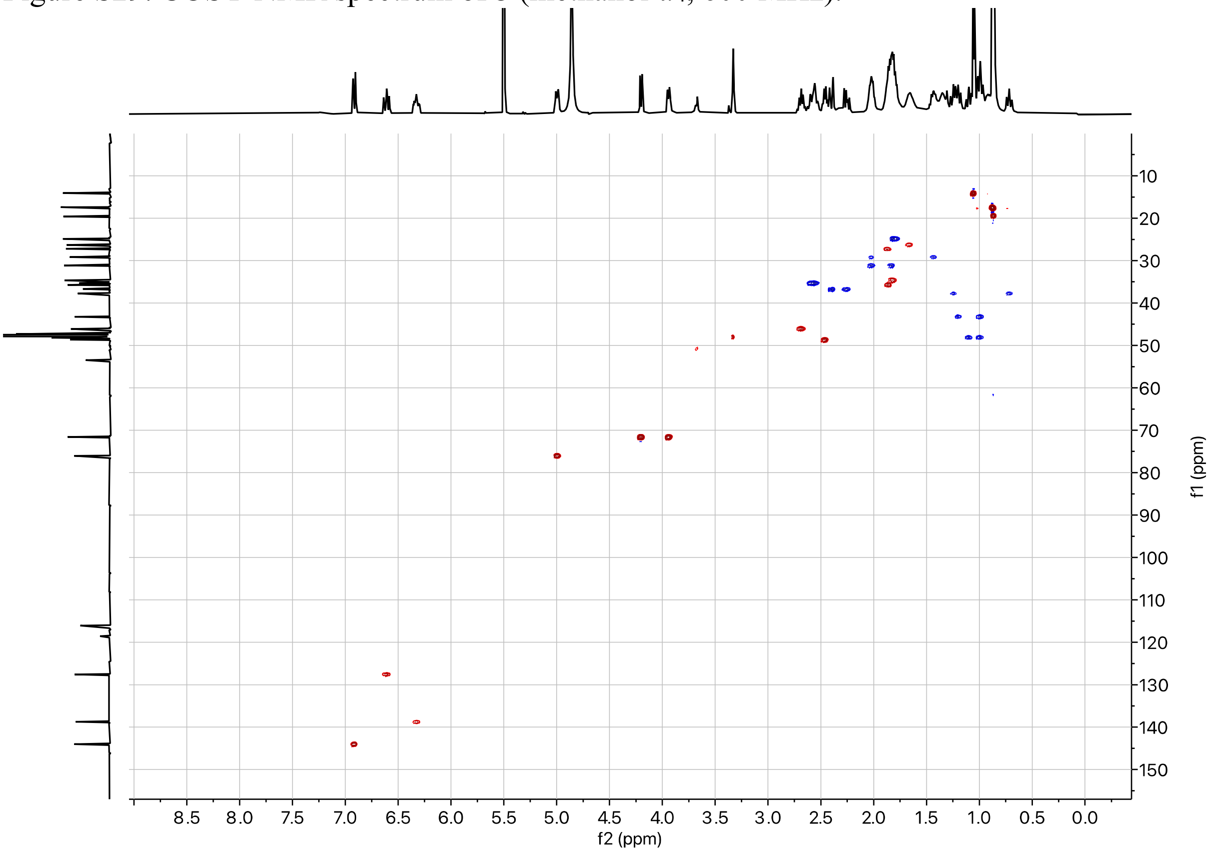

Figure S30. HSQC NMR spectrum of **5** (methanol-*d*4, 600 MHz).

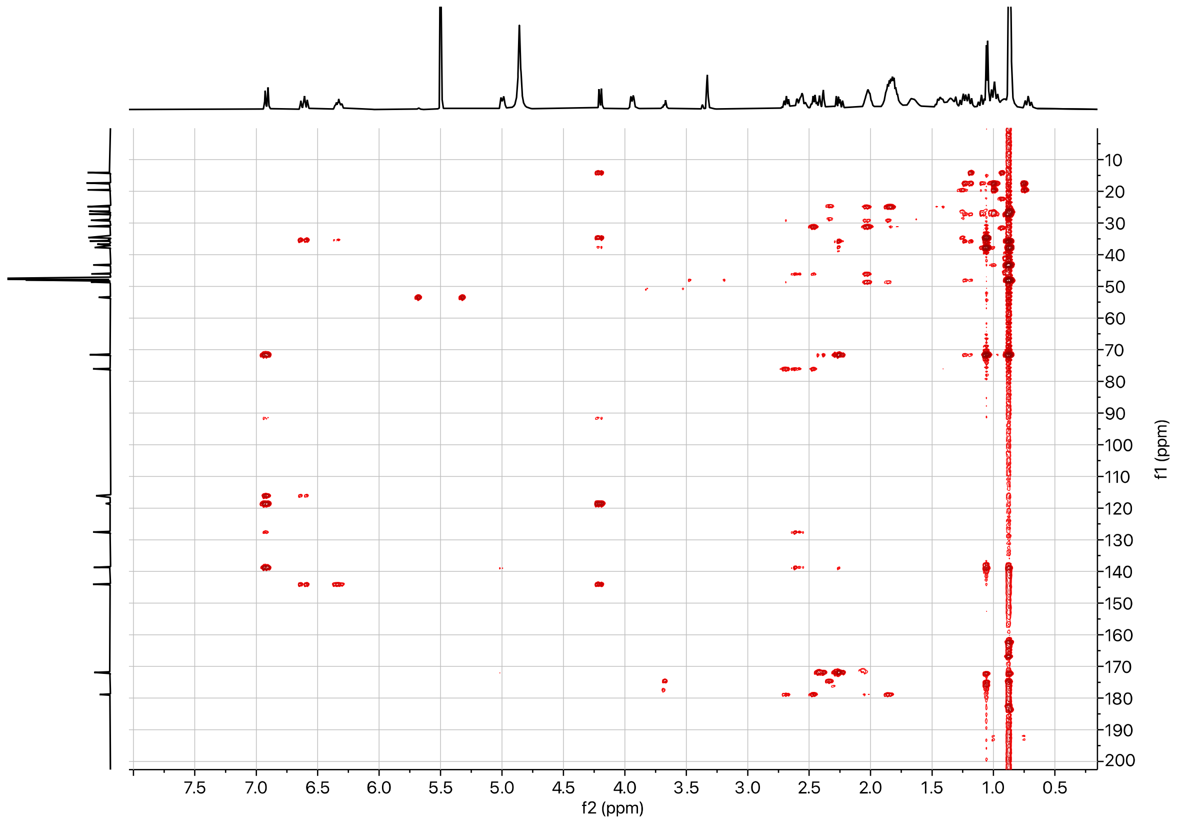

Figure S31. HMBC NMR spectrum of **5** (methanol-d_4, 600 MHz).

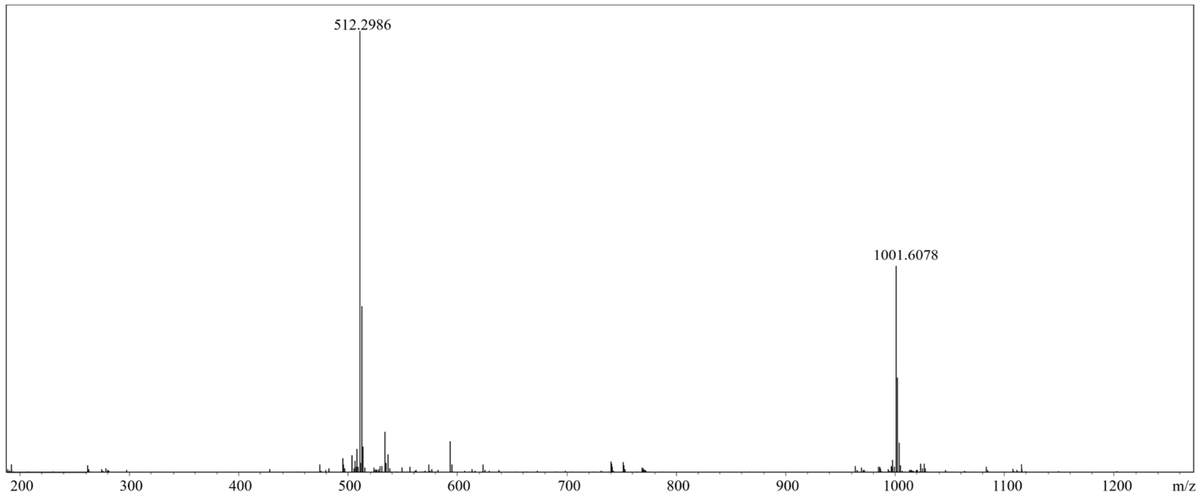

Figure S32. HRMS spectrum of **5**.

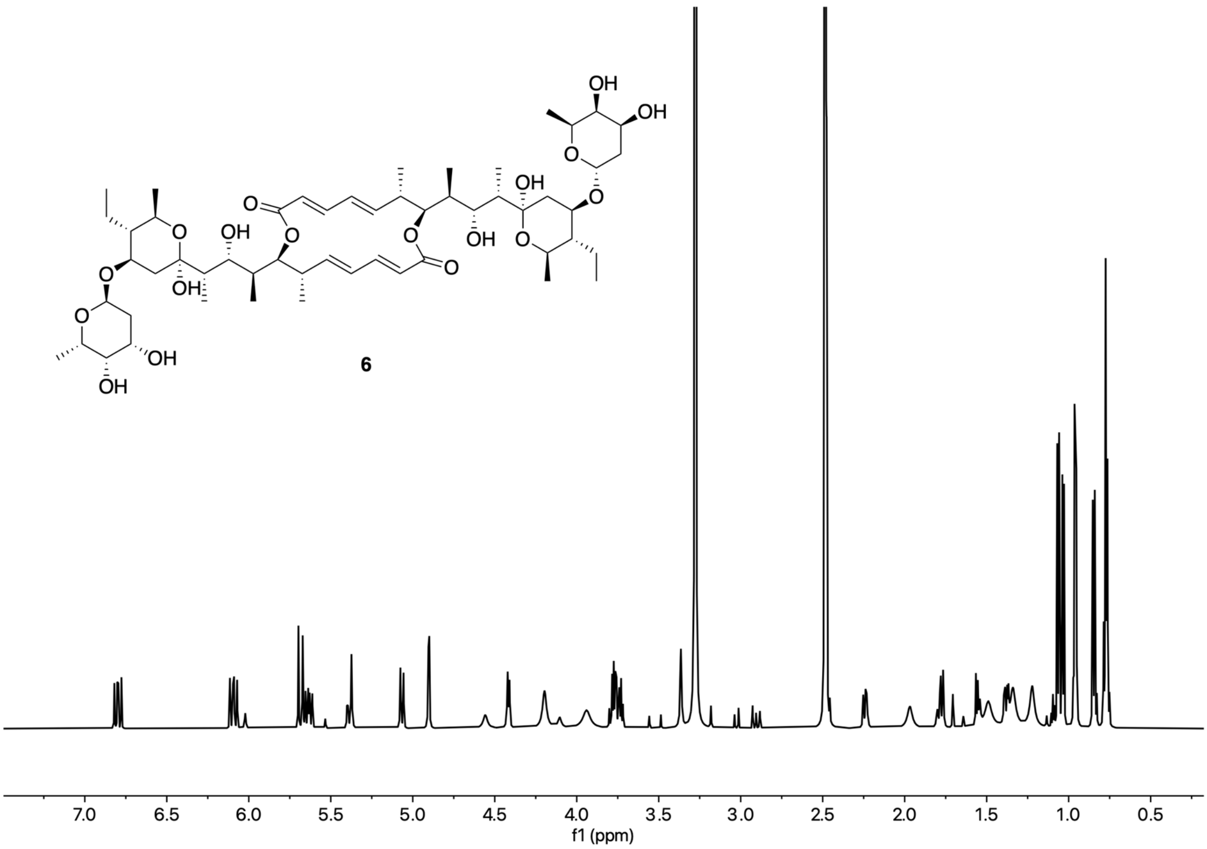

Figure S33. ^{1}H NMR spectrum of **6** (DMSO-d_6, 600 MHz).

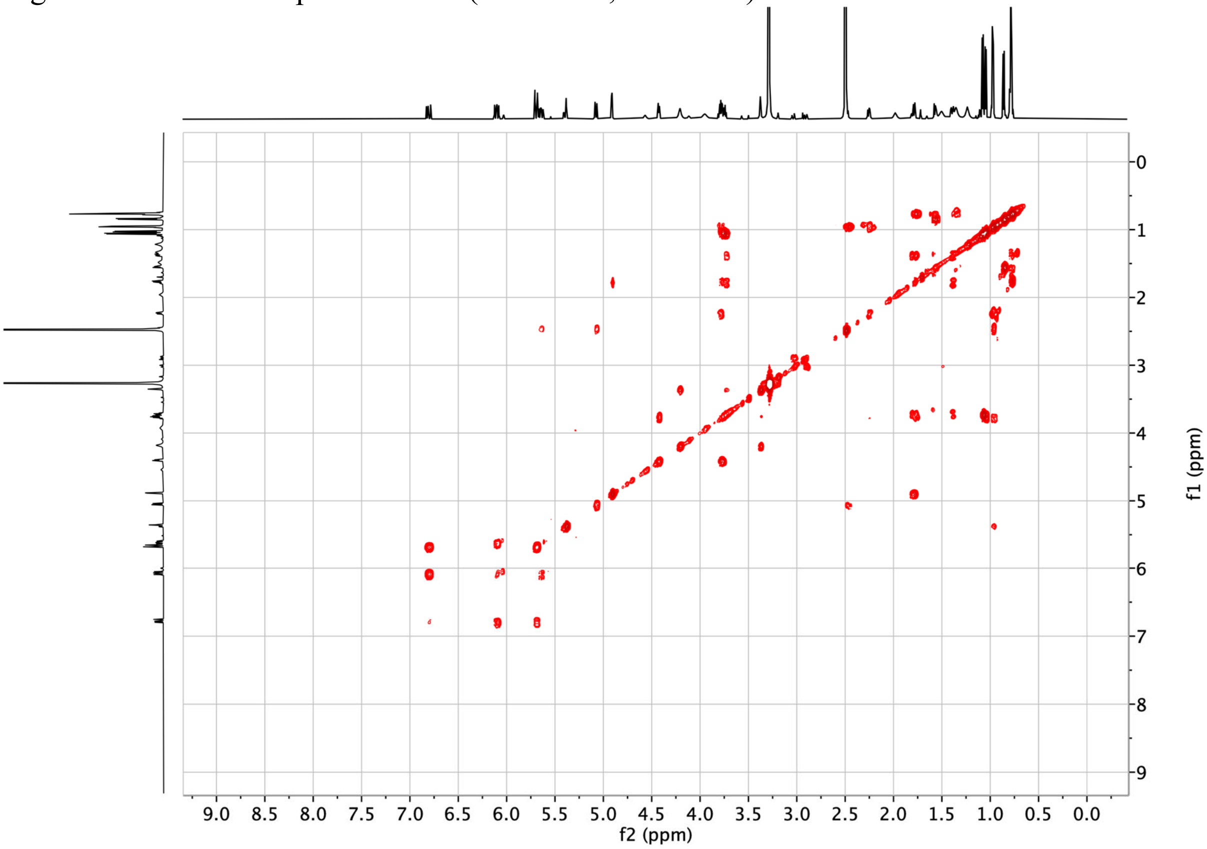

Figure S34. COSY NMR spectrum of **6** (DMSO-d_6, 600 MHz).

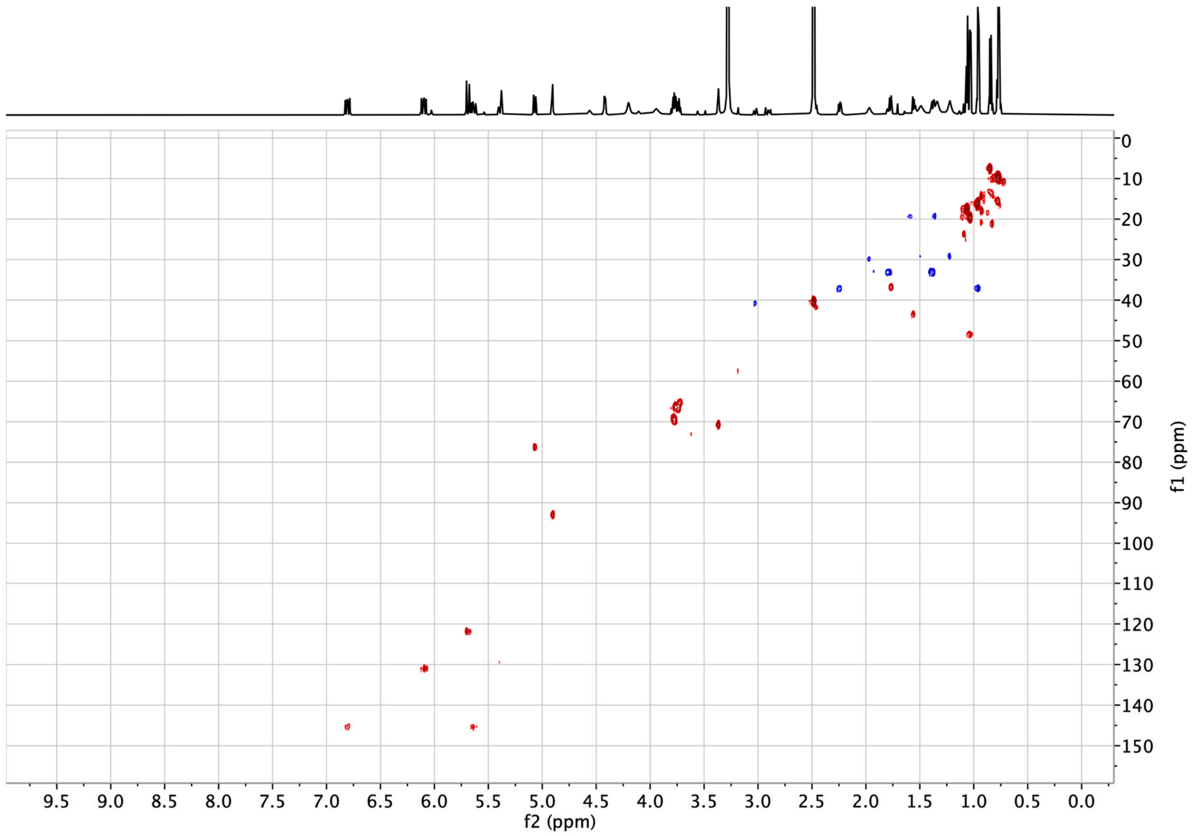

Figure S35. HSQC NMR spectrum of **6** (DMSO-d_6, 600 MHz).

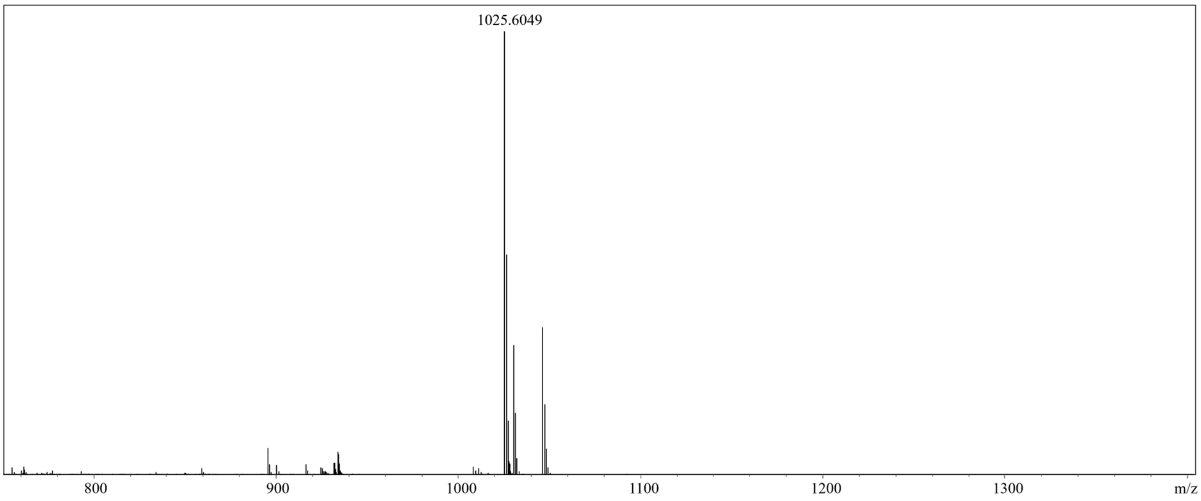

Figure S36. HRMS spectrum of **6**.

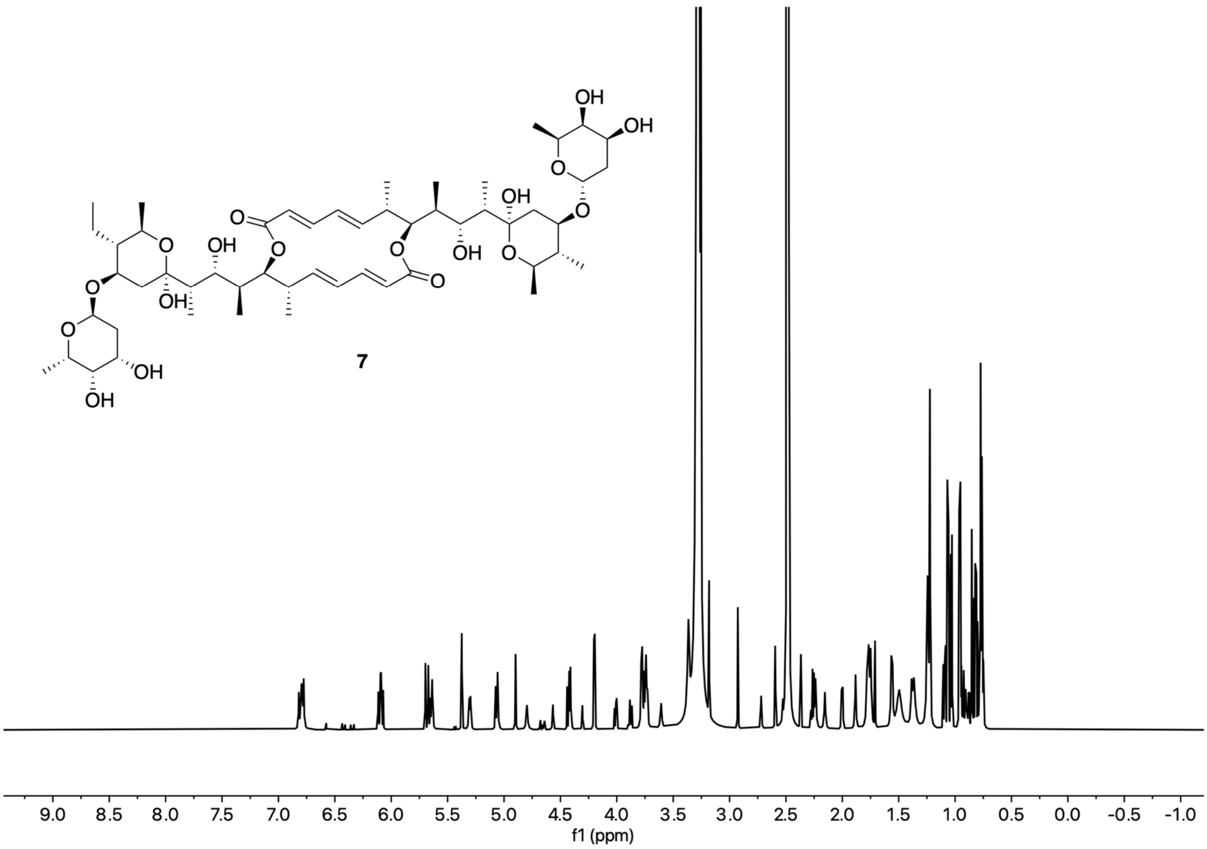

Figure S37. ^{1}H NMR spectrum of **7** (DMSO-d_6, 600 MHz).

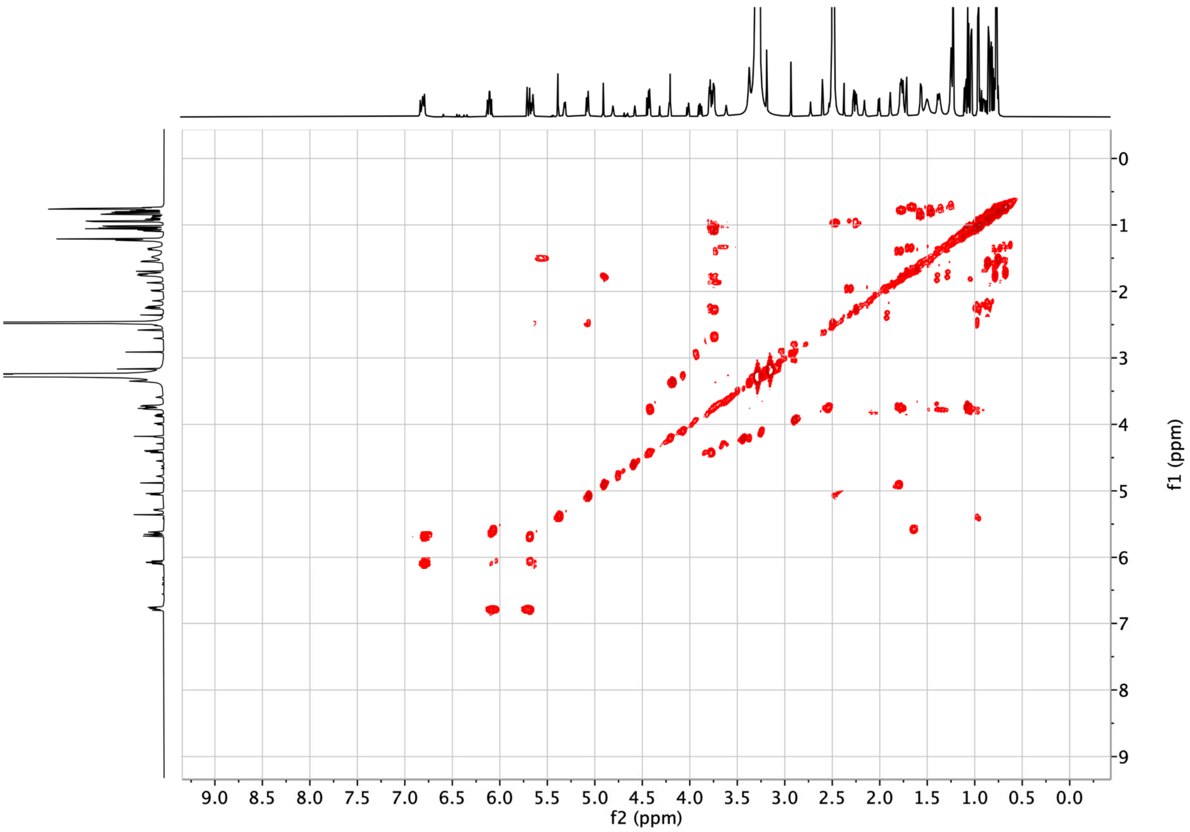

Figure S38. COSY NMR spectrum of **7** (DMSO-d_6, 600 MHz).

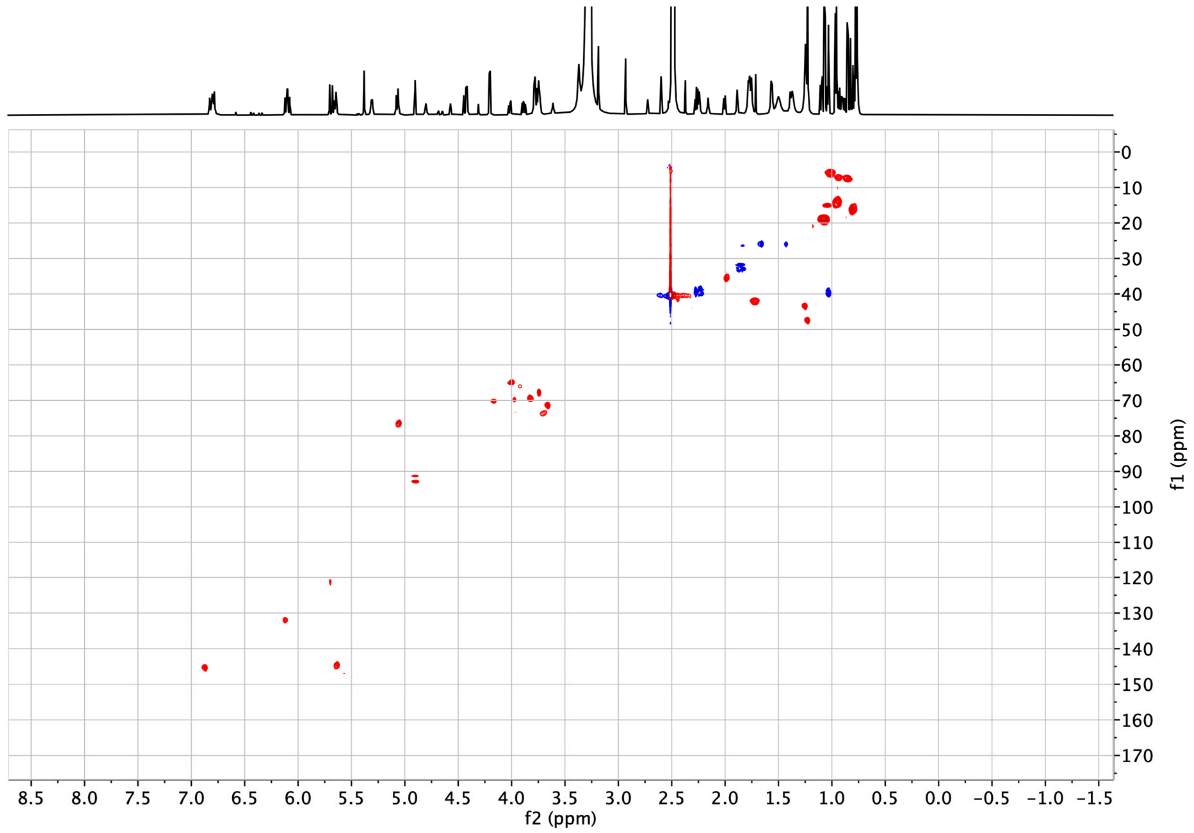

Figure S39. HSQC NMR spectrum of **7** (DMSO-d_6, 600 MHz).

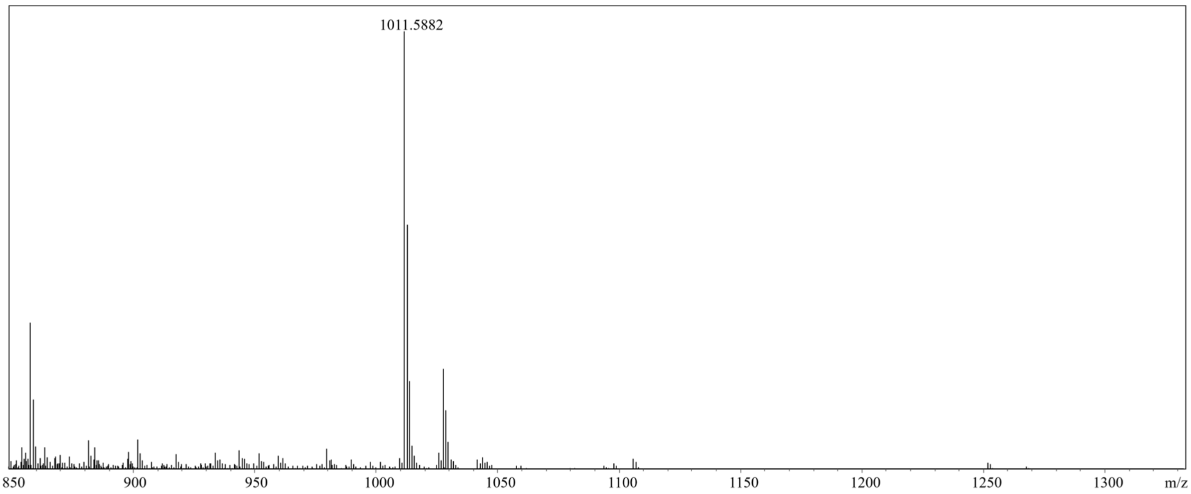

Figure S40. HRMS spectrum of **7**.

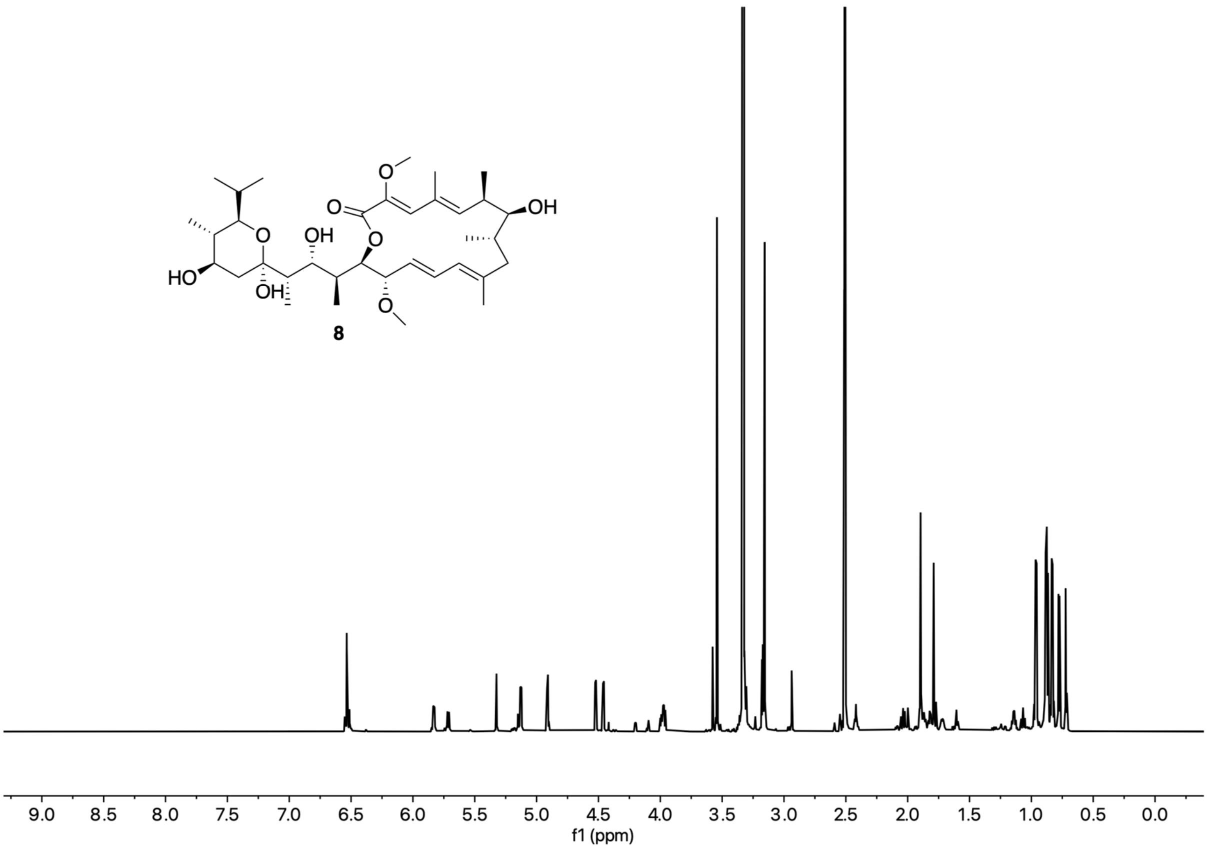

Figure S41. ^{1}H NMR spectrum of **8** (DMSO-d_6, 800 MHz).

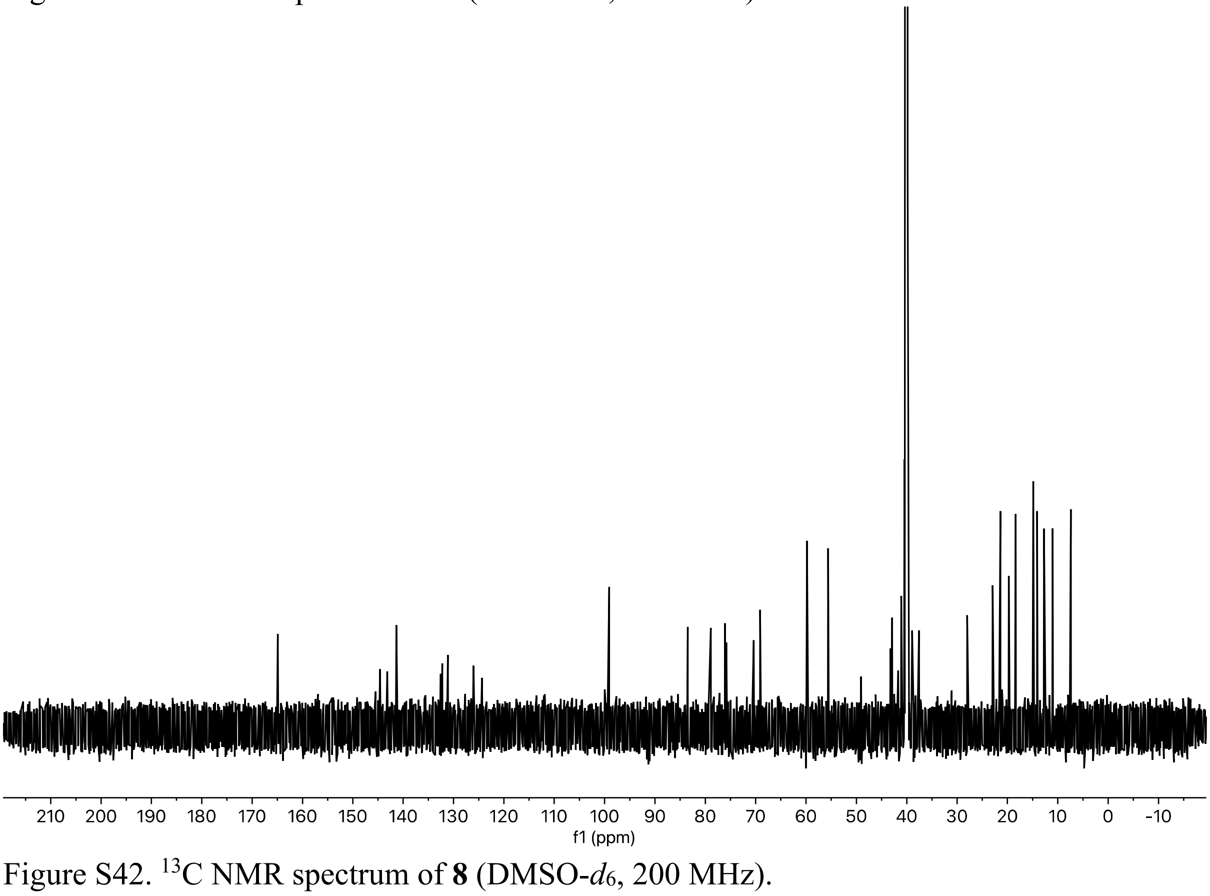

Figure S42. ^{13}C NMR spectrum of **8** (DMSO-d_6, 200 MHz).

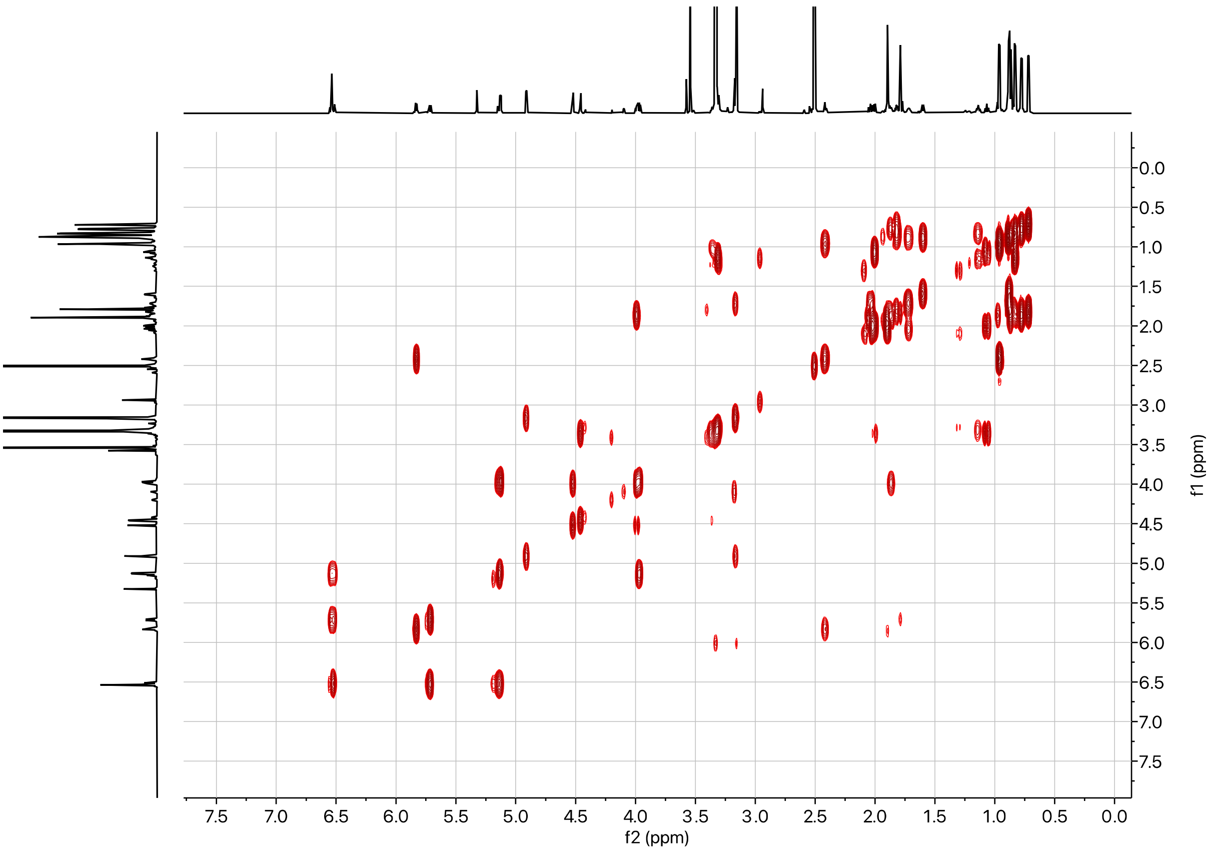

Figure S43. COSY NMR spectrum of **8** (DMSO-d_6, 800 MHz).

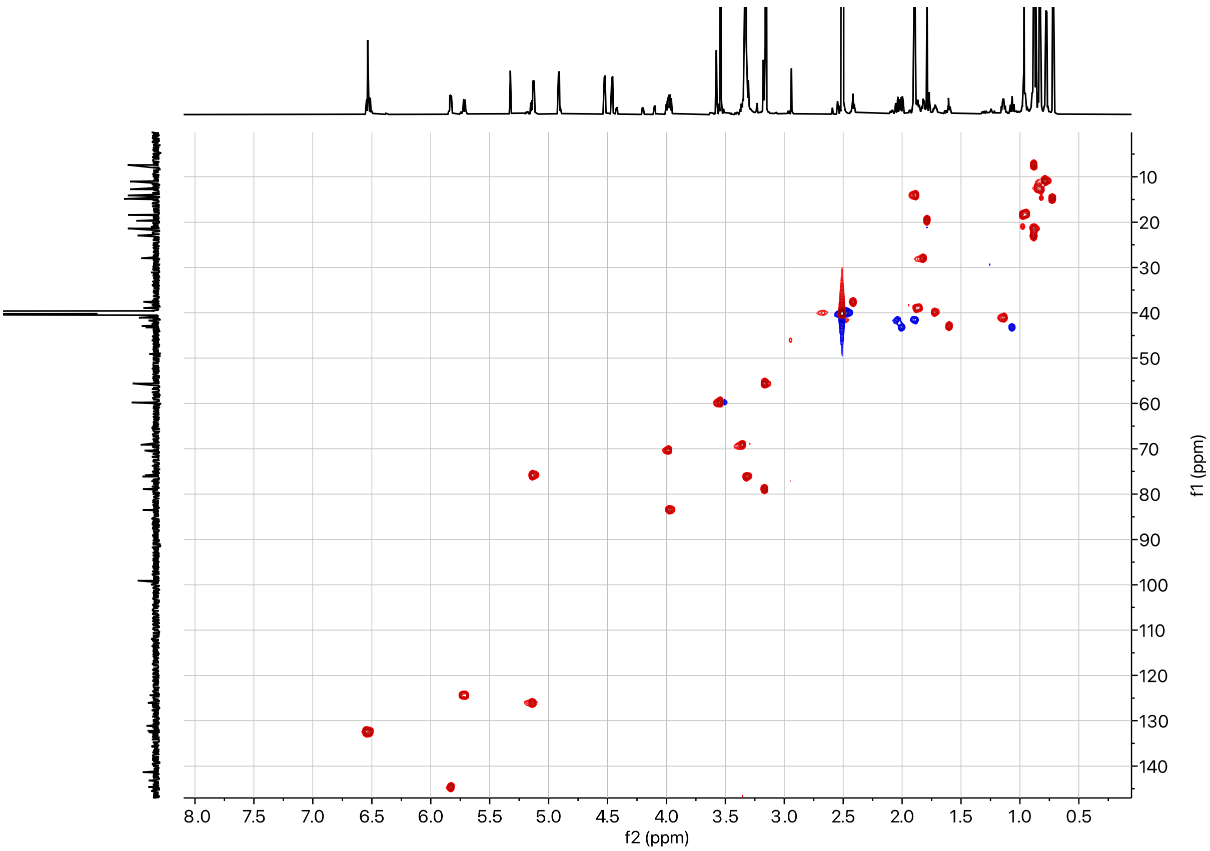

Figure S44. HSQC NMR spectrum of **8** (DMSO-d_6, 800 MHz).

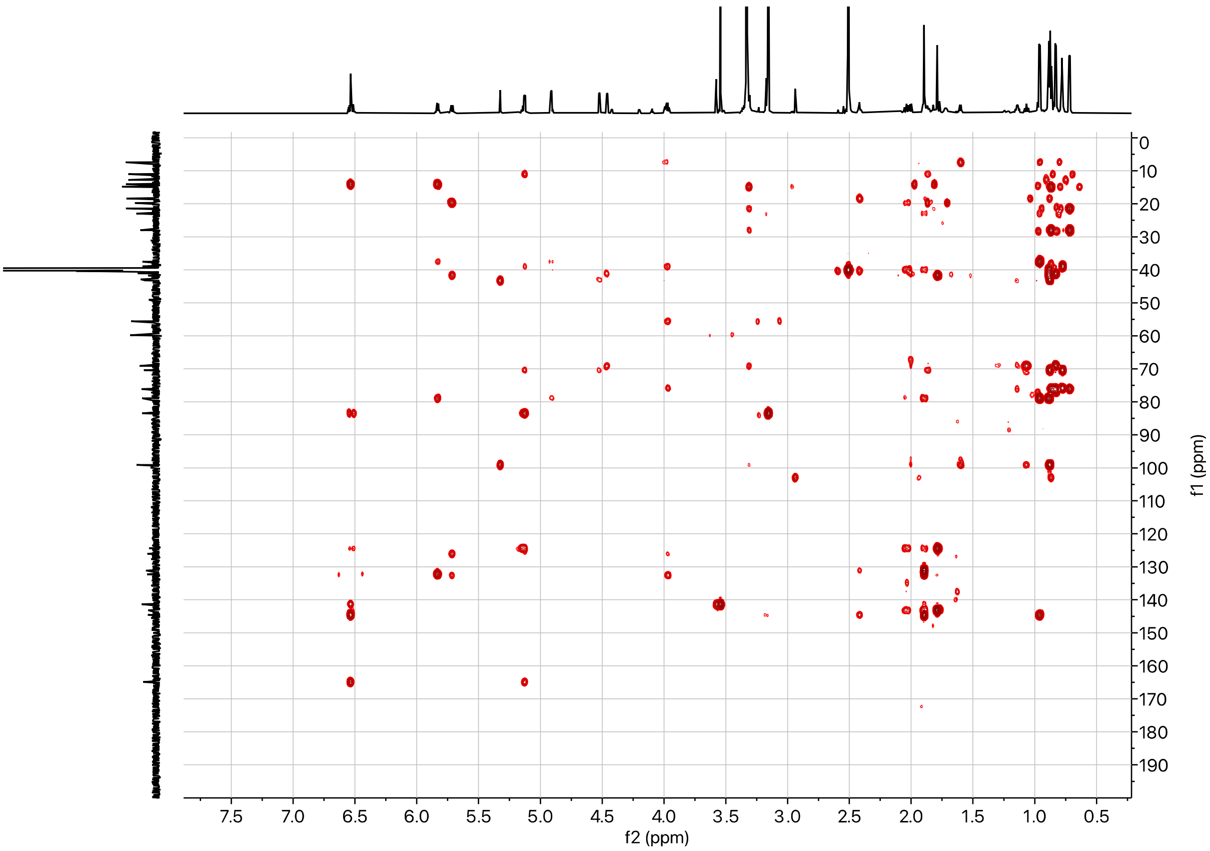

Figure S45. HMBC NMR spectrum of **8** (DMSO-d_6, 800 MHz).

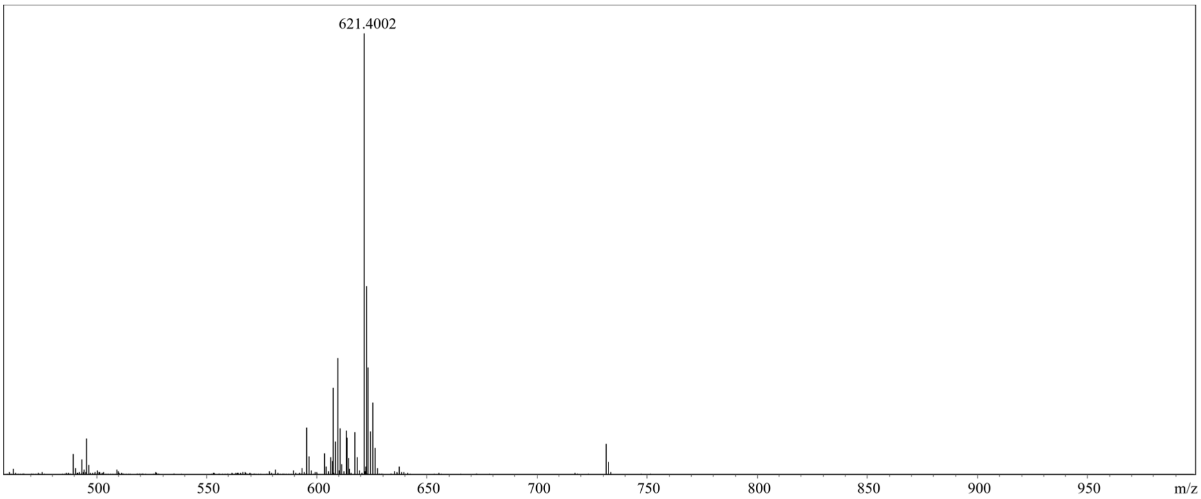

Figure S46. HRMS spectrum of **8**.

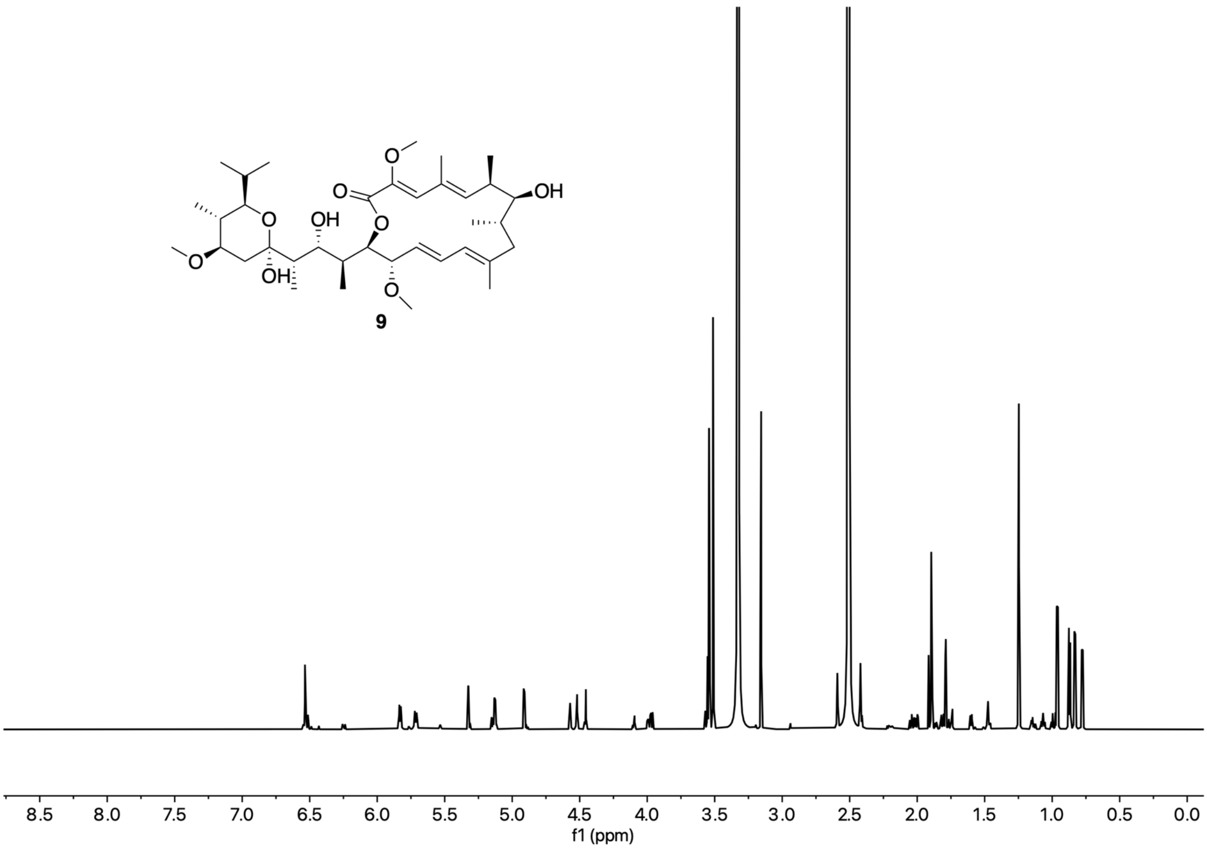

Figure S47. ^{1}H NMR spectrum of **9** (DMSO-d_6, 800 MHz).

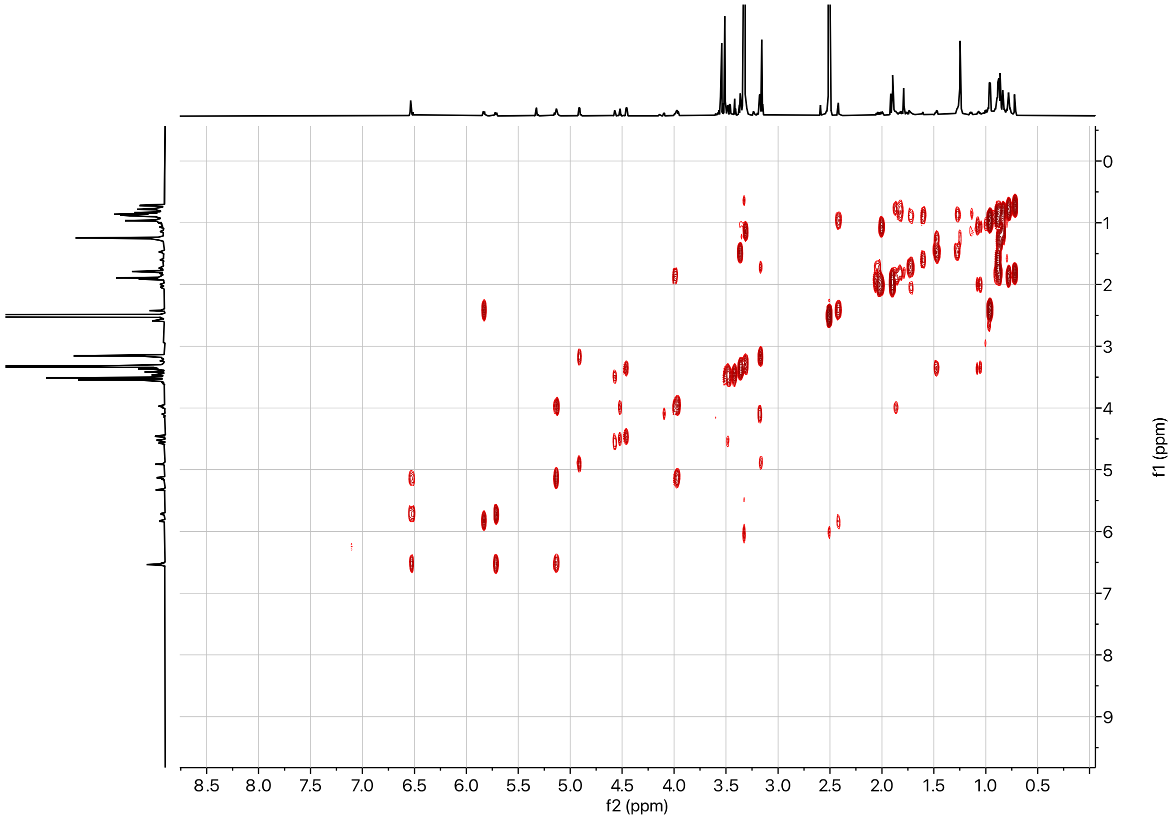

Figure S48. COSY NMR spectrum of **9** (DMSO-d_6, 800 MHz).

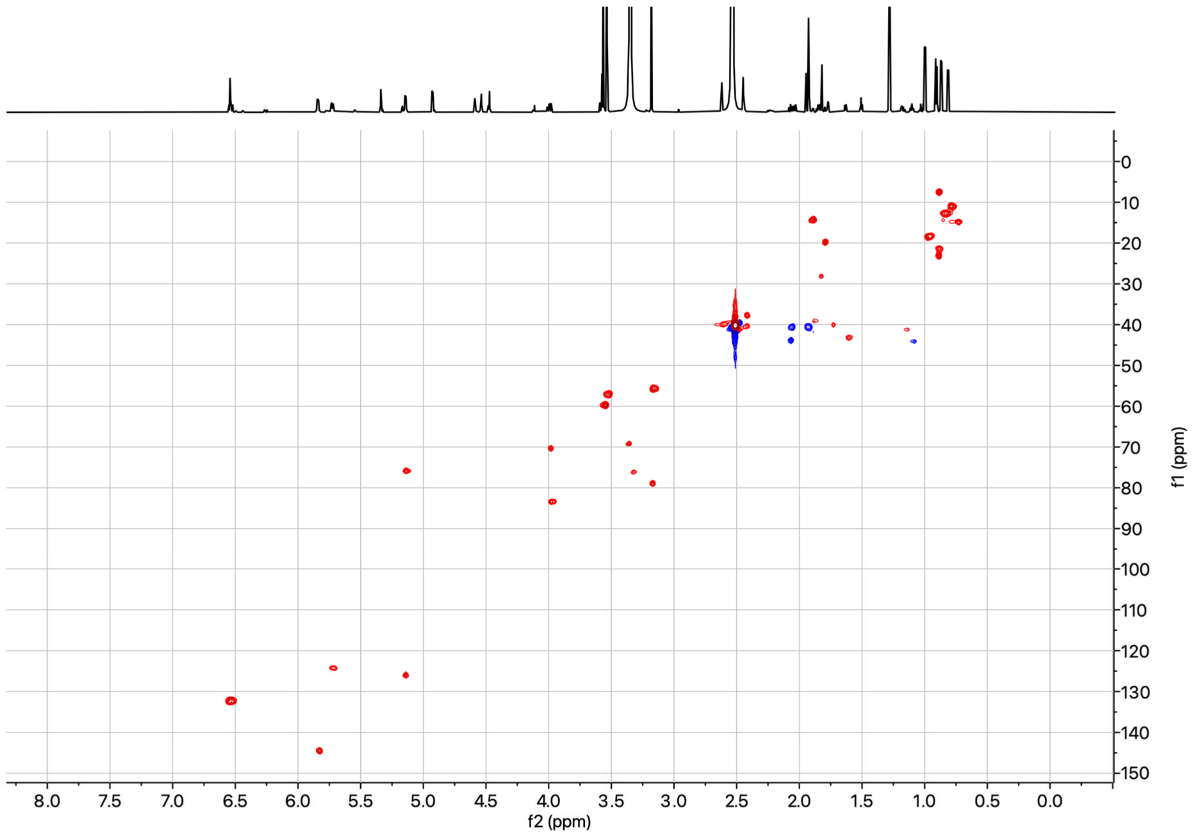

Figure S49. HSQC NMR spectrum of **9** (DMSO-d_6, 800 MHz).

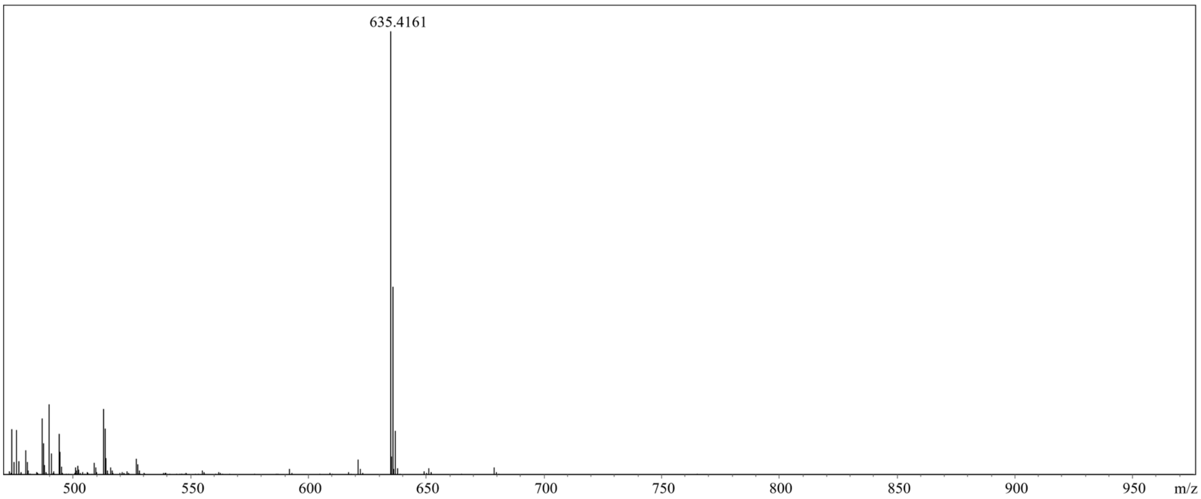

Figure S50. HRMS spectrum of **9**.

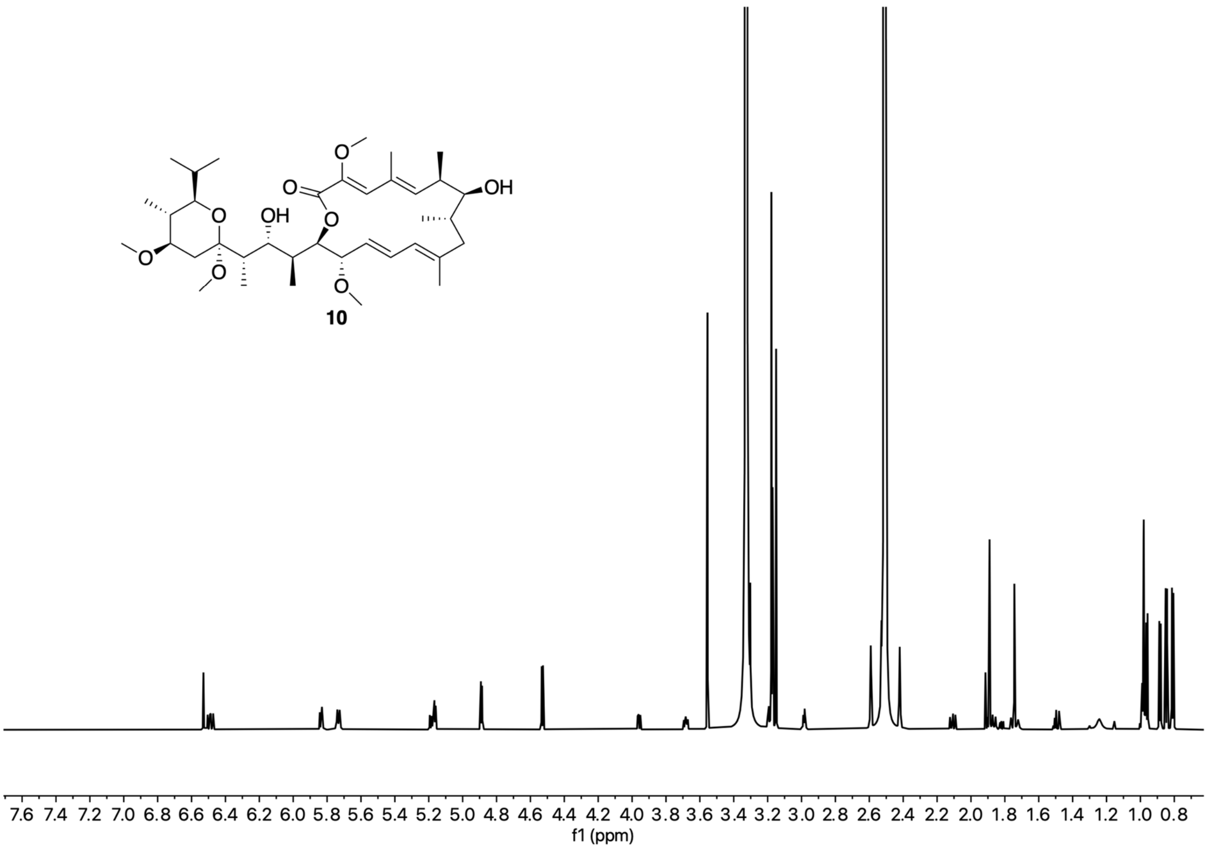

Figure S51. ^{1}H NMR spectrum of **10** (DMSO-d_6, 800 MHz).

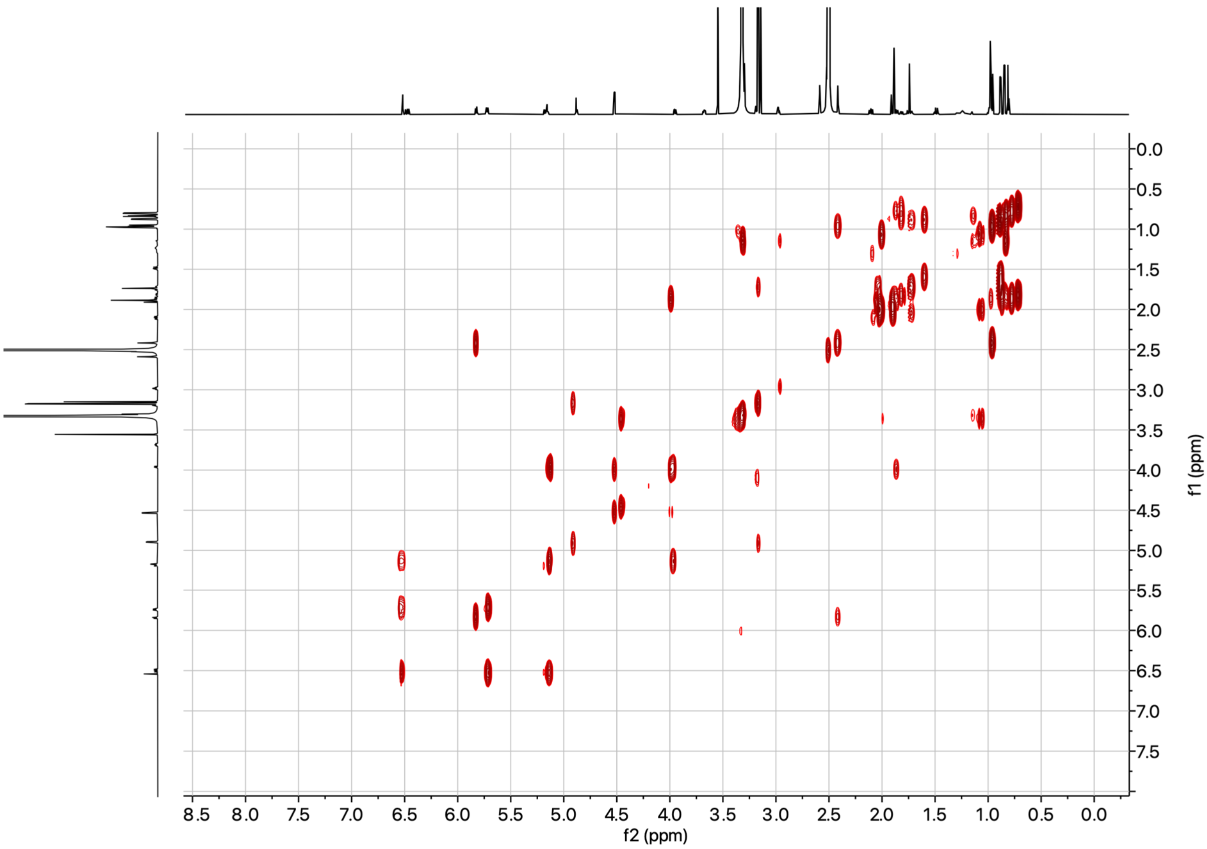

Figure S52. COSY NMR spectrum of **10** (DMSO-d_6, 800 MHz).

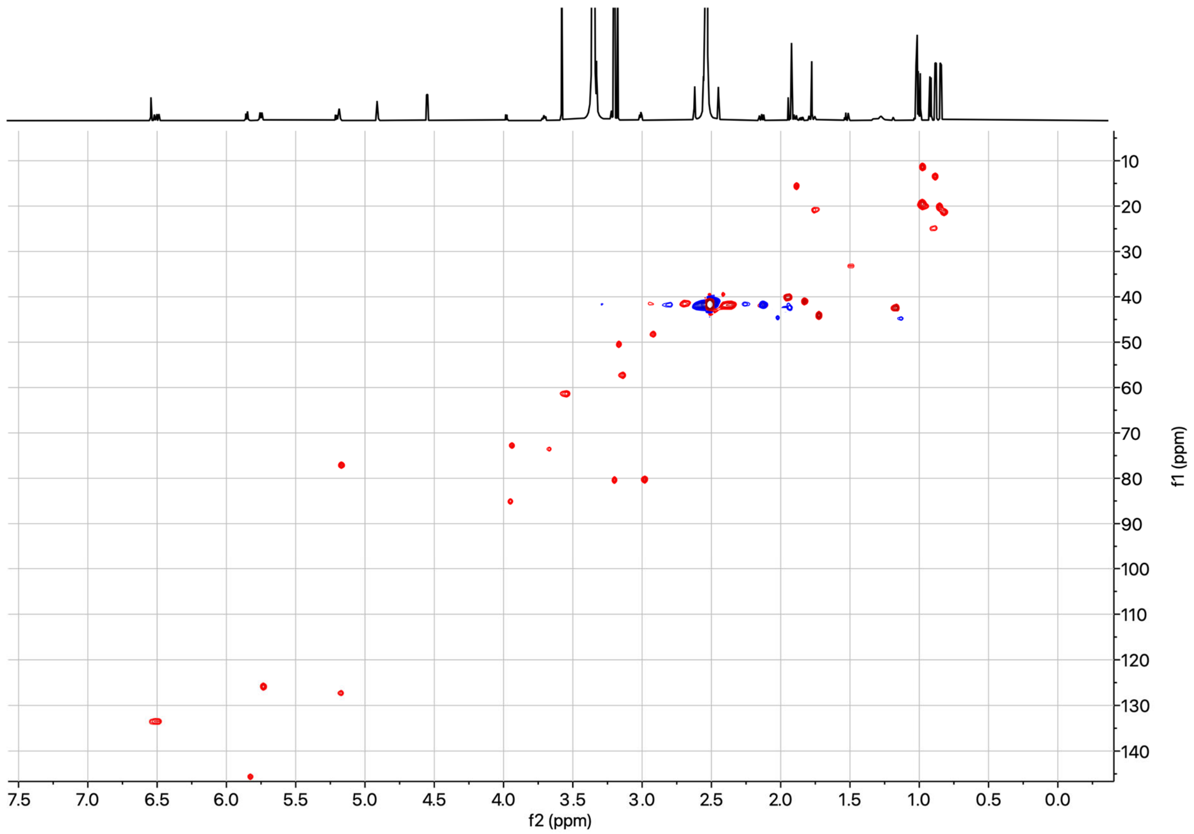

Figure S53. HSQC NMR spectrum of **10** (DMSO-d_6, 800 MHz).

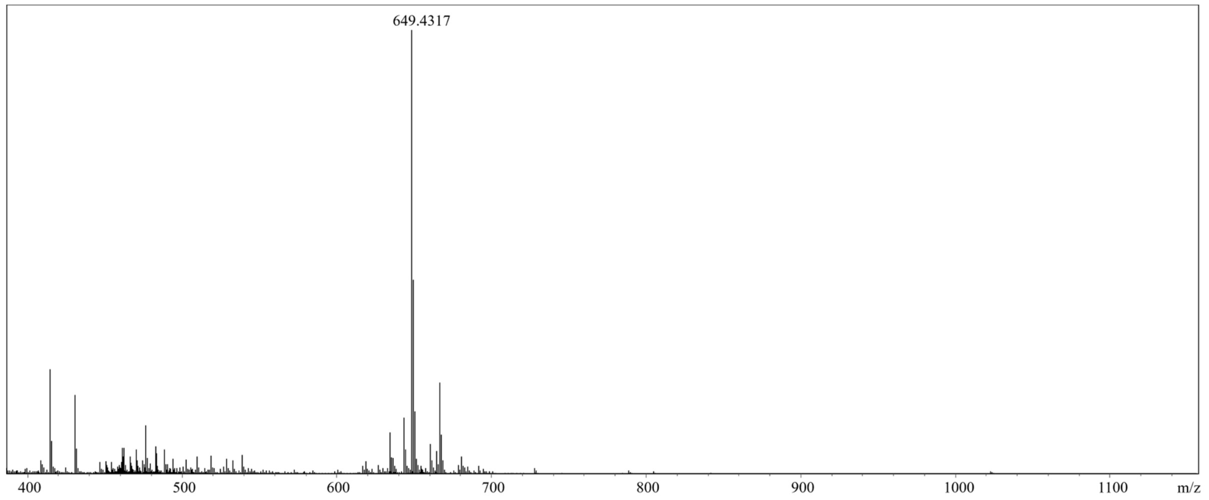

Figure S54. HRMS spectrum of **10**.

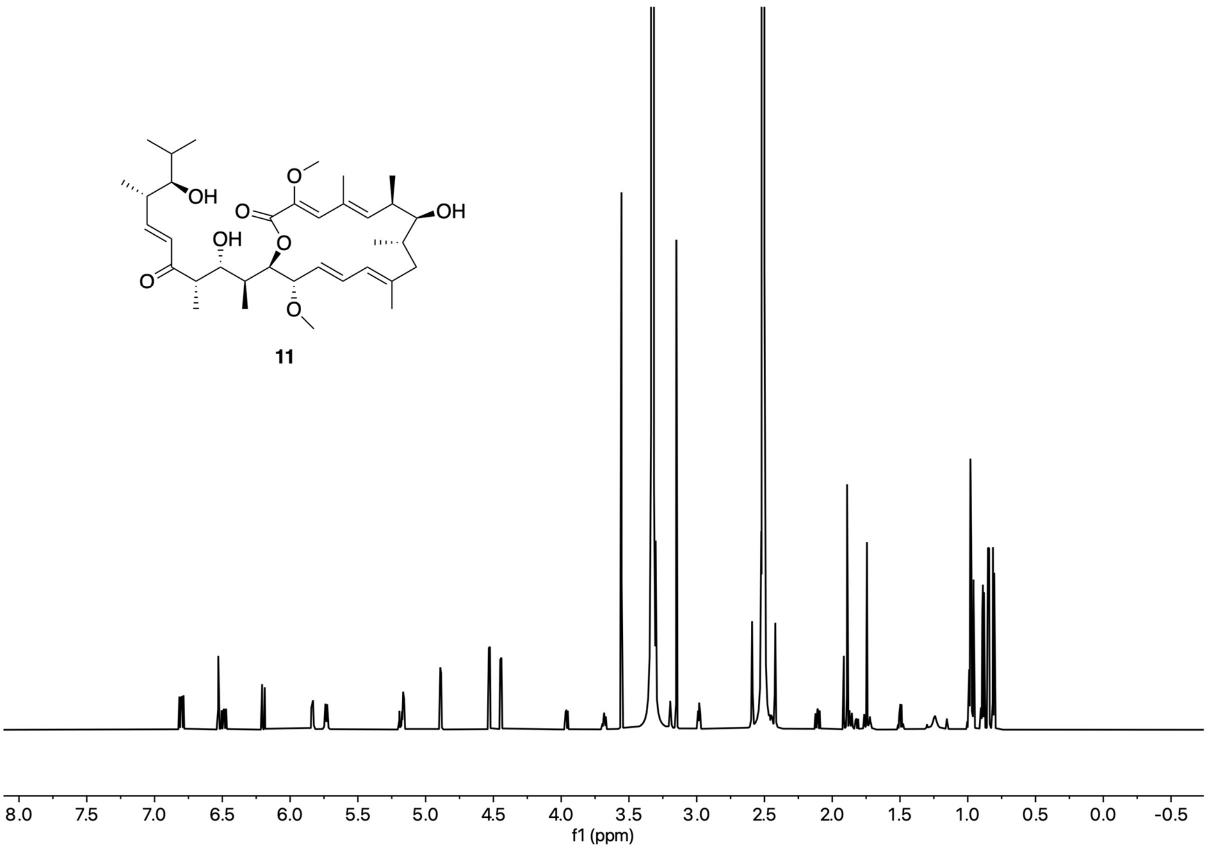

Figure S55. ^{1}H NMR spectrum of **11** (DMSO-*d*6, 800 MHz).

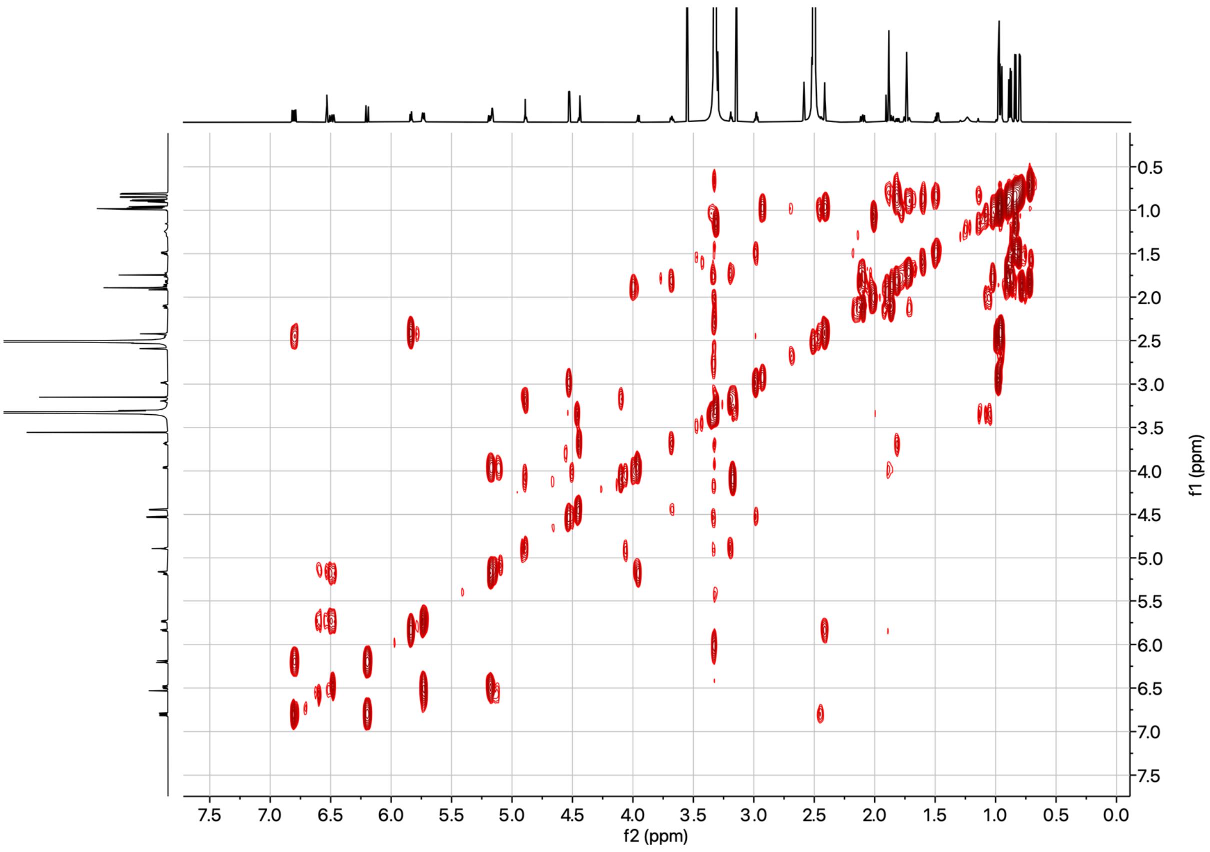

Figure S56. COSY NMR spectrum of **11** (DMSO-*d*6, 800 MHz).

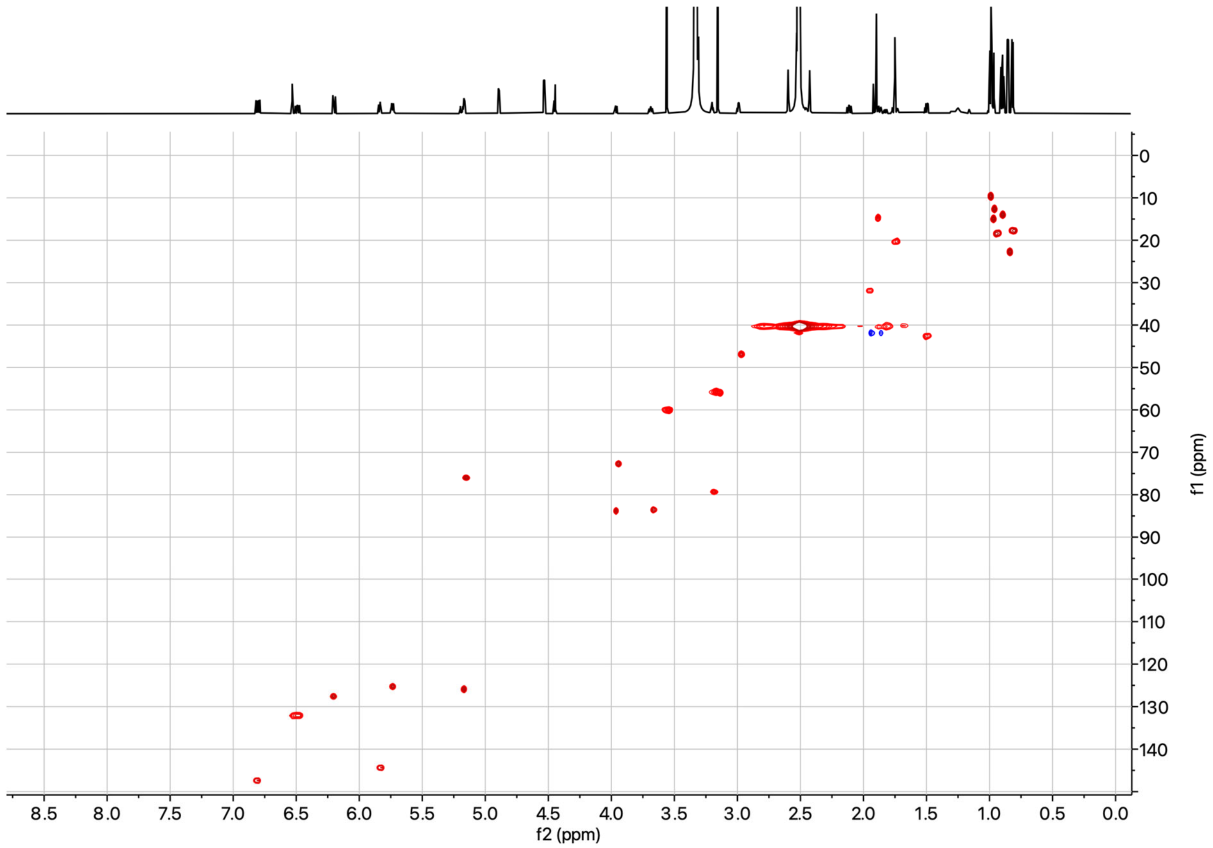

Figure S57. HSQC NMR spectrum of **11** (DMSO-d_6, 800 MHz).

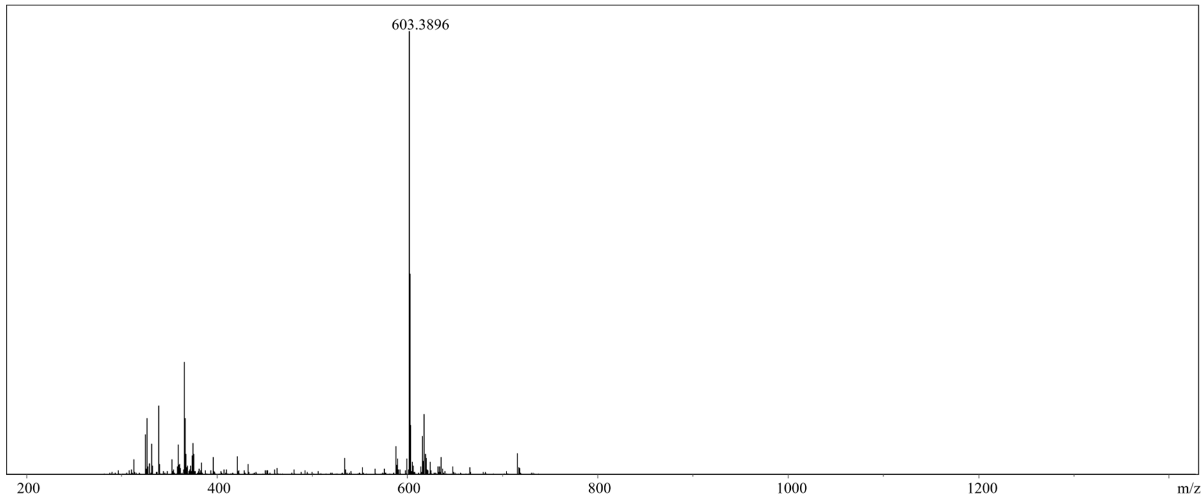

Figure S58. HRMS spectrum of **11**.
